# Kpna6 deficiency causes infertility in male mice by disrupting spermatogenesis

**DOI:** 10.1242/dev.198374

**Published:** 2021-10-04

**Authors:** Na Liu, Fatimunnisa Qadri, Hauke Busch, Stefanie Huegel, Gabin Sihn, Ilya Chuykin, Enno Hartmann, Michael Bader, Franziska Rother

**Affiliations:** 1 Max Delbrück Center for Molecular Medicine, Berlin 13125, Germany; 2 Medical Systems Biology Division, Lübeck Institute of Experimental Dermatology and Institute for Cardiogenetics, University of Lübeck, Lübeck 23562, Germany; 3 Institute for Biology, Center for Structural and Cellular Biology in Medicine, University of Lübeck, Lübeck 23562, Germany; 4 Department of Cell Developmental and Regenerative Biology, Icahn School of Medicine at Mount Sinai, New York, NY 10029-6574, USA

**Keywords:** Importin, Karyopherin, Spermatogenesis, Male fertility, Testis, Mouse

## Abstract

Spermatogenesis is driven by an ordered series of events, which rely on trafficking of specific proteins between nucleus and cytoplasm. The karyopherin α family of proteins mediates movement of specific cargo proteins when bound to karyopherin β. Karyopherin α genes have distinct expression patterns in mouse testis, implying they may have unique roles during mammalian spermatogenesis. Here, we use a loss-of-function approach to determine specifically the role of Kpna6 in spermatogenesis and male fertility. We show that ablation of Kpna6 in male mice leads to infertility and has multiple cumulative effects on both germ cells and Sertoli cells. Kpna6-deficient mice exhibit impaired Sertoli cell function, including loss of Sertoli cells and a compromised nuclear localization of the androgen receptor. Furthermore, our data demonstrate devastating defects on spermiogenesis, including incomplete sperm maturation and a massive reduction in sperm number, accompanied by disturbed histone-protamine exchange, differential localization of the transcriptional regulator BRWD1 and altered expression of RFX2 target genes. Our work uncovers an essential role of Kpna6 in spermatogenesis and, hence, in male fertility.

## Abstract (precis)

**Summary:** Two different mouse models delineate the morphological and functional impact of Kpna6 on spermatogenesis and Sertoli cell function and show that this protein is crucial for fertility in male mice.

## INTRODUCTION

The best-characterized mechanism of nuclear import involves karyopherin α and karyopherin β (also known as importin α and importin β). Karyopherin α proteins are composed of three main structural domains: an N-terminal region, which is the karyopherin (importin) β binding (IBB) domain; a central domain containing Armadillo motifs; and a weakly conserved C-terminal region. The central domain of karyopherin α binds nuclear localization signals that are present in the target cargo proteins. Upon cargo binding, karyopherin α binds to karyopherin β via its IBB domain forming a trimeric transport complex, which is translocated into the nucleus via karyopherin β interactions with nucleoporins lining the nuclear pore complex (Macara, 2001; Miyamoto et al., 2012). To date, three karyopherin α subtypes have been identified in *Caenorhabditis elegans* and *Drosophila melanogaster*, whereas up to seven karyopherin α isoforms have been found in mammals (Köhler et al., 1997; Tejomurtula et al., 2009; Tsuji et al., 1997).

Male reproductive function relies on normal spermatogenesis within the testis seminiferous epithelium. During spermatogenesis, spermatogonia undergo mitosis and differentiate into primary spermatocytes, which process through preleptotene, leptotene, zygotene, pachytene and diplotene stages of meiosis I to generate secondary spermatocytes. Subsequently, secondary spermatocytes enter the second meiotic division resulting in round spermatids. The haploid round spermatids undergo dramatic morphological changes, and finally differentiate into mature spermatozoa (Russell, 1990).

Sertoli cells are supporting somatic cells essential for the development of male germ cells. It has been shown that the number and function of Sertoli cells determine testicular size, germ cell number and spermatozoa output (Orth et al., 1988). Sertoli cell functions include provision of structural support and nutrition to developing germ cells, coordination of differentiation among several cohorts of germ cells, secretion of seminiferous fluid, phagocytosis of degenerating germ cells and release of spermatids at spermiation (Bellve and Zheng, 1989; Chihara et al., 2013; Clermont, 1993; Russell and Griswold, 1993).

A key feature of Sertoli cell structural support for developing germ cells is the blood-testis barrier (BTB), which consists of tight junctions (TJs) located between adjacent Sertoli cells (Johnson et al., 2008). At the beginning of meiosis, preleptotene spermatocytes ‘pass through’ the BTB. Once the BTB has reformed behind them, the germ cells no longer have access to serum factors and become totally dependent upon Sertoli cells to supply nutrients and growth factors (Walker, 2010). This structural arrangement creates an immunological barrier by isolating advanced germ cells from the immune system so that their antigens do not stimulate autoimmunity (Johnson et al., 2008; Orth et al., 1988).

The successful completion of spermatogenesis is dependent on successive division and differentiation steps, which require multiple changes in gene expression, coordinated by transcription and other factors expressed within the testis (Eddy and O'Brien, 1998; Hermo et al., 2010). Access of these factors to the nucleus is tightly regulated for these proteins, and it has been postulated that germ cell differentiation is controlled by nucleocytoplasmic transport events (Major et al., 2011). In fact, the mRNAs of different karyopherin α isoforms and of karyopherin β are all expressed in germ cells and Sertoli cells (Major et al., 2011; Shima et al., 2004), raising the possibility that the karyopherin α/β-mediated nuclear import pathway is involved in the regulation of spermatogenesis and Sertoli cell function. With respect to protein expression in murine testis, it is currently known that Kpna4 (also known as importin α3) localizes to nuclei of Sertoli cells, pachytene spermatocytes and round spermatids step 7-8, whereas Kpna3 (also known as importin α4) is expressed in the cytoplasm of Sertoli cells, mitotic and meiotic spermatocytes as well as round spermatids (Hogarth et al., 2007). Furthermore, our own unpublished data revealed a very distinct expression of Kpna2 (also known as importin α1) in meiotic germ cells of mouse testis. The protein expression of Kpna6 in the murine testis has not been evaluated so far and a specific role of a single karyopherin α isoform in spermatogenesis and male reproduction has not been determined.

We have previously shown that in Kpna6-deficient mothers, embryonic development stops at the two-cell stage owing to severely disturbed zygotic genome activation; therefore, Kpna6 is essential for early embryonic development in mice (Rother et al., 2011). Here, we show that ablation of *Kpna6* results in a critical defect in spermatogenesis in mice. We demonstrate that Kpna6 protein is expressed in the nuclei of round spermatids, elongating spermatids and Sertoli cells. Consistent with this pattern, Kpna6 deficiency results in multiple defects in both germ cells and Sertoli cells culminating in oligozoospermia. Our results demonstrate an essential role for Kpna6 in male fertility by regulating spermatogenesis and Sertoli cell function.

## RESULTS

### Kpna6 is essential for male fertility

**Fig. 1.**
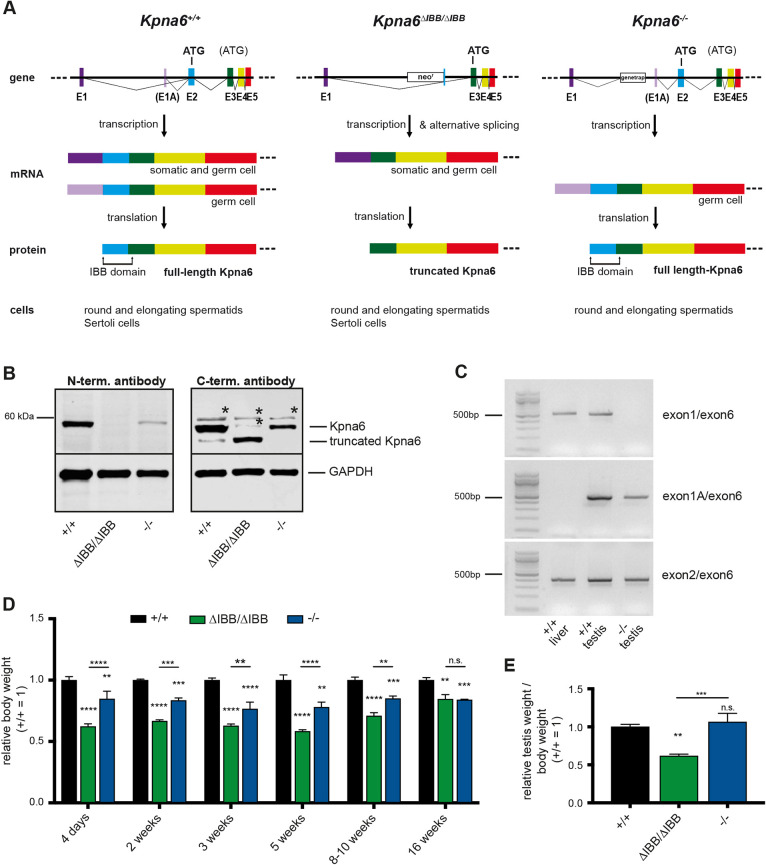
**Disruption of Kpna6 causes growth retardation and reduced testis size.** (A) Gene targeting strategy for *Kpna6*^ΔIBB/ΔIBB^ and *Kpna6*^−/−^ mice. In *Kpna6*^ΔIBB/ΔIBB^, exon 2 is replaced by a neomycin resistance (neo^r^) cassette with a polyadenylation site (pA). Because transcription does not always stop at pA, a splicing variant is generated, carrying an in-frame translational start site in exon 3, resulting in the formation of a truncated protein. In *Kpna6*^−/−^ mice, a gene trap is located in intron 1, leading to complete loss of the protein in most of the tissues. However, a testis-specific exon 1A (E1A) allows the generation of a germ cell-specific mRNA resulting in a full-length protein. (B) Western blot analysis of Kpna6 expression in testes. The 58 kDa protein is absent in *Kpna6*^ΔIBB/ΔIBB^ testes, but it can be detected in *Kpna6*^−/−^ and WT (+/+) testes (left). A Kpna6^ΔIBB^ protein that is about 10 kDa smaller than the full-length protein is found in the testis of *Kpna6*^ΔIBB/ΔIBB^ mice (right). Asterisks mark nonspecific cross-reactions of the antibody. (C) RT-PCR of WT liver, WT testis and *Kpna6*^−/−^ testis using primer pairs spanning different exons of *Kpna6*. In WT liver and testis, a transcript spanning exon 1 and exon 6 can be detected, but it is absent in *Kpna6*^−/−^ testis. WT testis, but not WT liver, expresses a specific transcript using exon 1A and this transcript can also be detected in *Kpna6*^−/−^ testis. All tested tissues express transcripts spanning exons 2-6. (D) Relative body weight at various ages (*n*=6 per group). (E) Relative testis weight at 8-10 weeks of age (*n*=6 per group).

We generated two mouse lines with targeted disruption of Kpna6. In *Kpna6*^ΔIBB/ΔIBB^ mice, as a result of unexpected alternative splicing, a shortened mRNA is generated, containing a cryptic translational start site that leads to synthesis of a truncated protein lacking the karyopherin β binding domain (Fig. 1A, Fig. S1A). In *Kpna6*^−/−^, a gene trap cassette in intron 1 of *Kpna6* results in a complete loss of the protein (Fig. 1A). Female mice of both lines are infertile (Rother et al., 2011).

Interestingly, male *Kpna6*^−/−^ mice are fertile, whereas *Kpna6*^ΔIBB/ΔIBB^ males were found to be sterile, although they were sexually active and produced vaginal plugs in female partners. We observed that, although Kpna6 protein is missing in all other organs of *Kpna6*^−/−^ males (Rother et al., 2011), full-length Kpna6 protein is still expressed in the testis, whereas it is completely absent from *Kpna6*^ΔIBB/ΔIBB^ testes (Fig. 1B). The reason for the exclusive expression in the testis of *Kpna6*^−/−^ males is that an alternative promoter and exon 1 (exon 1A) are used, which are located downstream of the gene trap cassette (Fig. 1A-C, Fig. S1A; confirmed by sequencing; identical to BY353738.1). This leads to synthesis of a full-length and fully functional Kpna6 protein (as the regular translational start site is located in exon 2). By contrast, only a truncated non-functional Kpna6 was found in the *Kpna6*^ΔIBB/ΔIBB^ testes (Fig. 1B), which resulted in male infertility, suggesting that Kpna6 is essential for male fertility. Sequencing of the *Kpna6* mRNA variants in testis revealed that in *Kpna6*^−/−^ testis only the shorter transcript was present at expression levels comparable to WT. In contrast, in *Kpna6*^ΔIBB/ΔIBB^ testes, the mRNA levels were strongly reduced as transcription from exon 1A does not occur (Fig. S1A). Analysis of fluorescence-activated cell sorting (FACS)-sorted round spermatids revealed that both transcript variants were present in WT (Fig. S1B).

### Disruption of *Kpna6* causes growth retardation, reduced testis size and severe oligozoospermia

The *Kpna6*^ΔIBB/ΔIBB^ and *Kpna6*^−/−^ mice were born at a lower frequency (*Kpna6*^ΔIBB/ΔIBB^: 18.8%, *n*=739; *P*<0.0001; *Kpna6*^−/−^: 16%, *n*=214, *P*=0.0015) than predicted by Mendelian laws. With regards to growth and development, the heterozygous males are indistinguishable from wild-type (WT) males (Fig. S2A). However, *Kpna6*^ΔIBB/ΔIBB^ pups displayed severe growth retardation in the postnatal phase, and this growth defect persisted until adult life (Fig. 1D). Male *Kpna6*^−/−^ mice also displayed a significant growth retardation, although the effect in young mice was not so strong. At the age of 16 weeks, males of both mutant lines displayed the same reduction in body weight compared with WT males (Fig. 1D). Testes of adult *Kpna6*^ΔIBB/ΔIBB^ mice exhibited a pronounced reduction both in size and weight (Fig. S2B,C) and the testicular weight to body weight ratio was reduced by 40% at the age of 8-10 weeks compared with WT and *Kpna6*^−/−^ mice, which displayed a normal relative testis weight and size (Fig. 1E). Serum testosterone levels were unchanged (Fig. S2D).

**Fig. 2.**
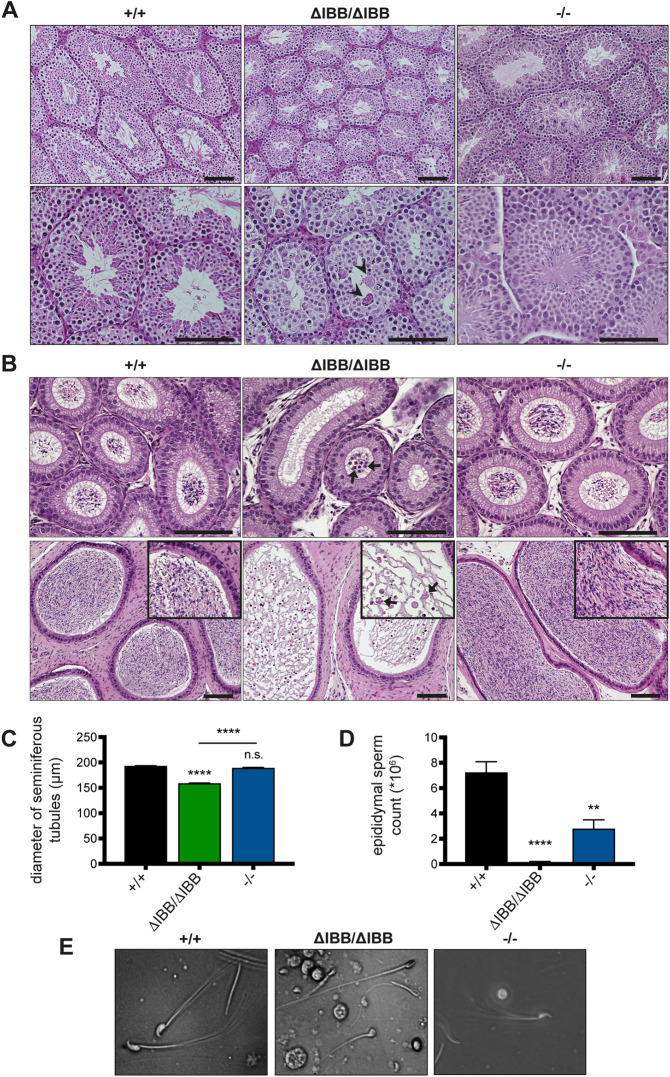
**Disruption of Kpna6 causes oligozoospermia.** (A,B) H&E staining of testis (A) and epididymis (B; top row: caput; bottom row: cauda) sections. Arrowheads in A indicate multinucleated spermatid giant cells; arrows in B indicate immature germ cells (top row) or sloughed immature germ cells (bottom row). Insets show details at higher magnifications. (C) Diameters of seminiferous tubules. (D) Epididymal sperm count (*Kpna6*^+/+^: *n*=6; *Kpna6*^ΔIBB/ΔIBB^: *n*=4; *Kpna6*^−/−^: *n*=8). (E) Representative images of epididymal sperms from WT, *Kpna6*^ΔIBB/ΔIBB^ and *Kpna6*^−/−^ mice. Age of mice: 12-16 weeks. Scale bars: 100 µm.

Histological analyses revealed that spermatogenesis was drastically altered in *Kpna6*^ΔIBB/ΔIBB^, whereas no major changes were detected in *Kpna6*^−/−^ compared with WT testes (Fig. 2A). Seminiferous tubules in the *Kpna6*^ΔIBB/ΔIBB^ testes were smaller in diameter than those in both other groups (Fig. 2A,C). The germ cell number was reduced, and the tubular epithelium was disorganized. Moreover, mature spermatozoa were rarely found in the lumen of *Kpna6*^ΔIBB/ΔIBB^ seminiferous tubules, but multinucleated spermatid giant cells were frequently observed (Fig. 2A). There were very few spermatozoa in the caput of *Kpna6*^ΔIBB/ΔIBB^ epididymides, and spermatozoa were hardly detectable in the caudal epididymides by Hematoxylin and Eosin (H&E) staining (Fig. 2B). Additionally, sloughed germ cells and germ cell debris were commonly observed in the epididymal lumen of *Kpna6*^ΔIBB/ΔIBB^ males (Fig. 2B). The total cauda epididymal sperm number in *Kpna6*^ΔIBB/ΔIBB^ was only 1.4% of that of WT males (Fig. 2D); moreover, almost all of the residual sperms found in the *Kpna6*^ΔIBB/ΔIBB^ epididymides displayed abnormal heads in contrast to *Kpna6*^−/−^ and WT sperms (Fig. 2E). Surprisingly, *Kpna6*^−/−^ sperm count was also significantly reduced, suggesting partially reduced fertility in these mice (Fig. 2D). In both lines, the epididymal sperm count of heterozygous mice was normal (Fig. S2E).

### Kpna6 expression pattern in mouse testis

**Fig. 3.**
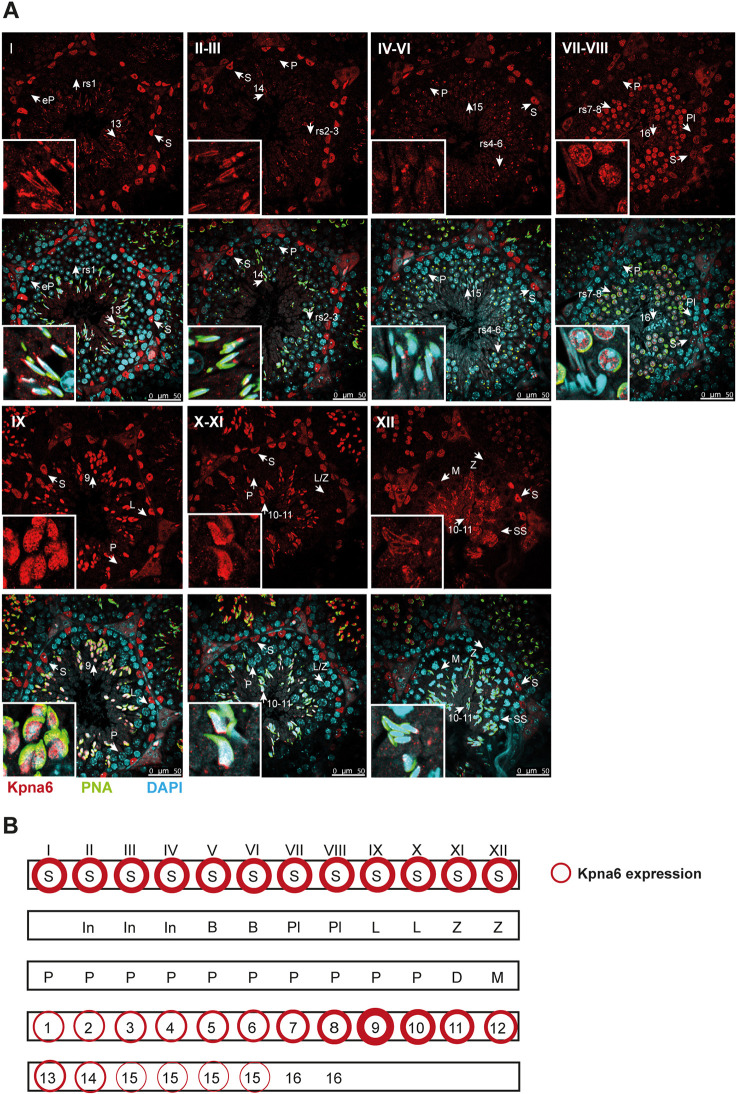
**Kpna6 is expressed in postmeiotic spermatids and Sertoli cells.** (A) Immunohistochemistry of testis sections of adult mice (12-16 weeks) using an antibody against the C terminus of Kpna6 (red), counterstained with DAPI (blue) and PNA, labeling the acrosome (green). Roman numbers mark tubular stages. Insets show details of germ cells at higher magnification. Scale bars: 50 µm. (B) Schematic of Kpna6-expressing cell types in mouse testis. B, type B spermatogonium; D, diakinesis spermatocyte; eP, early pachytene spermatocyte; In, intermediate spermatogonium; L, leptotene spermatocyte; L/Z, leptotene/zygotene spermatocyte; M, meiosis; P, pachytene spermatocyte; Pl, preleptotene spermatocyte; rs, round spermatid; S, Sertoli cell; SS, secondary spermatocyte; Z, zygotene spermatocyte. Arabic numbers mark developmental steps of spermatids. Thickness of the red circles indicates varying expression levels.

To assess the cell type-specific expression of Kpna6 in the testis, we performed immunohistochemistry in WT mice using an antibody that detects the C terminus of Kpna6 (Fig. 3). No Kpna6 could be detected in spermatogonia and meiotic spermatocytes. Early round spermatids showed very low levels of expression, which increased throughout their development (steps 1-8), reaching its highest expression in step 9 elongating spermatids (stage IX), where Kpna6 displayed a high nuclear and low cytoplasmic expression. With the onset of nuclear elongation, localization of Kpna6 shifted to the cytoplasm, and was no longer detectable after the residual bodies were removed in step 16 sperms (stage VII-VIII). Kpna6 was highly expressed in the nuclei of Sertoli cells in all stages of the seminiferous epithelium (Fig. 3). The massive increase of Kpna6 expression in step 9 elongating spermatids and the high expression level in the nuclei of Sertoli cells suggest an important role of Kpna6 in these cells.

**Fig. 4.**
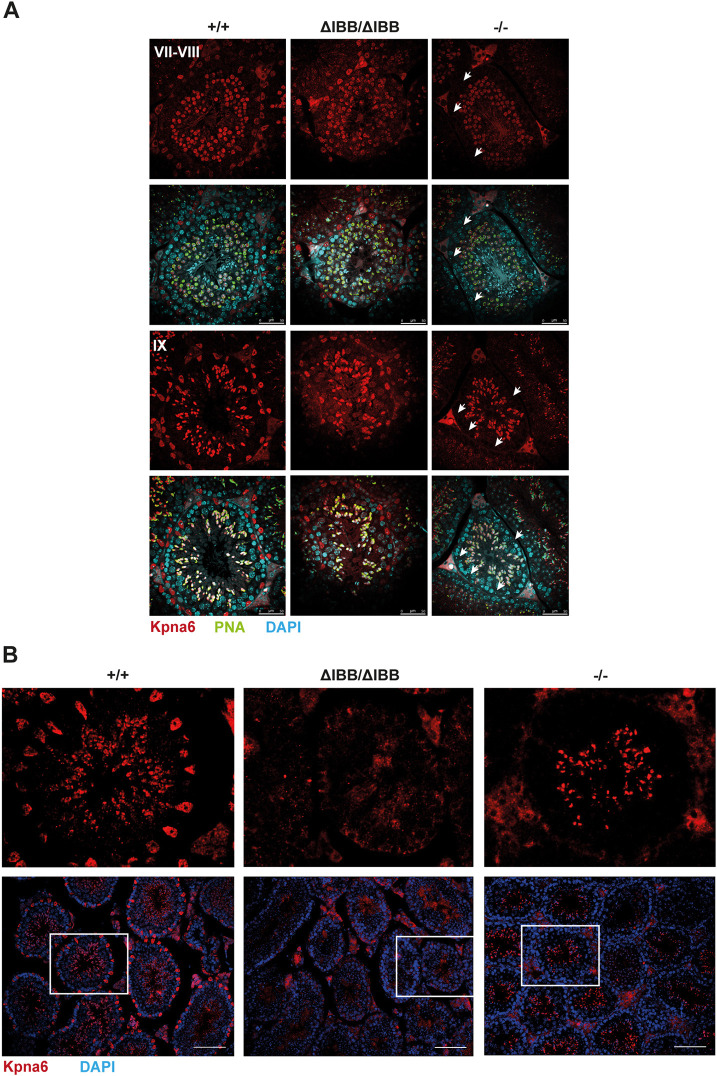
**Kpna6 is expressed in developing germ cells, but not in Sertoli cells, of *Kpna6*^−/−^ mice.** (A) Immunohistochemistry of testes using an antibody against the C terminus of Kpna6 (red); DAPI (blue); PNA (green). Note the absence of Kpna6 in nuclei of Sertoli cells in *Kpna6*^−/−^ (arrows). Scale bars: 50 µm. (B) Immunohistochemistry of testis sections using an antibody against the N terminus of Kpna6 (red); DAPI (blue). No specific staining is observed in *Kpna6*^ΔIBB/ΔIBB^ testis, as the antibody does not recognize the truncated protein. Kpna6 is expressed in round and elongating spermatids, but not in Sertoli cells of *Kpna6*^−/−^ mice, whereas WT mice show a robust immunostaining in Sertoli cells (top row shows higher magnification of the boxed areas in the bottom row). Age of mice: 12-16 weeks. Scale bars: 100 µm.

As the antibody against the C terminus of Kpna6 detects the full-length and the truncated form of the protein, it showed staining comparable to WT in *Kpna6*^ΔIBB/ΔIBB^ testes. Interestingly, testis sections of *Kpna6*^−/−^ mice revealed that the protein expression is rescued in germ cells, but not in Sertoli cells in this mouse line (Fig. 4A). Spatial and temporal expression of Kpna6 in *Kpna6*^−/−^ germ cells was similar to that in WT (Fig. 4A).

To verify these results, we generated an antibody against the N terminus of Kpna6 that could discriminate between the full-length and the truncated ΔIBB-protein, in which the N terminus is missing (Fig. 1A,B). Staining of testis sections of WT mice showed a robust signal in round and elongating spermatids as well as Sertoli cells. In contrast, no signals could be detected in *Kpna6*^ΔIBB/ΔIBB^ testis sections, confirming the truncation of Kpna6 in these mice. In *Kpna6*^−/−^ testes, the rescued expression in developing spermatids could be verified at the mRNA and protein levels, but no expression was found in Sertoli cells (Fig. 4B, Fig. S2B). The missing expression of Kpna6 in Sertoli cells could thus account for the reduced sperm cell number observed in *Kpna6*^−/−^ mice; however, only *Kpna6*^ΔIBB/ΔIBB^ mice are infertile, suggesting that expression of the protein in germ cells is indispensable for normal sperm development and fertility.

Thus, *Kpna6*^−/−^ mice express a mild Sertoli cell-related phenotype, whereas *Kpna6*^ΔIBB/ΔIBB^ mice express a mixed phenotype consisting of Sertoli cell- and germ cell-related defects. Although the infertile *Kpna6*^ΔIBB/ΔIBB^ mice express only truncated Kpna6 protein in Sertoli cells and in developing sperms, heterozygous *Kpna6*^ΔIBB/+^ mice express full-length plus truncated protein; however, sperm count in these mice turned out to be completely normal (Fig. S2E), excluding a dominant-negative effect of the truncated protein on sperm count. Moreover, a dominant-negative effect on growth defects could also be excluded (Fig. S2F). To discriminate between Sertoli cell- and germ cell-related phenotypes, we compared mice of both lines (*Kpna6*^ΔIBB/ΔIBB^ mice versus *Kpna6*^−/−^ mice). We rescued the germ cell phenotype without rescuing the Sertoli cell phenotype in *Kpna6*^ΔIBB/ΔIBB^ mice by crossing *Kpna6*^ΔIBB/ΔIBB^ and *Kpna6*^−/−^ mice. The resulting compound heterozygous *Kpna6*^ΔIBB/−^ mice expressed only the truncated Kpna6 in Sertoli cells and truncated plus full-length Kpna6 in developing sperms (Fig. S2G,H). Epididymal sperm count revealed a significant increase of sperm number in *Kpna6*^ΔIBB/−^ mice compared with *Kpna6*^ΔIBB/ΔIBB^ mice (Fig. S2E), suggesting that full-length Kpna6 in developing sperms can indeed partially rescue the oligozoospermia. Moreover, the sperm count in *Kpna6*^ΔIBB/−^ was markedly lower than in WT and comparable to *Kpna6*^−/−^ mice, confirming that the absence of full-length Kpna6 in Sertoli cells is the reason for the partial reduction in sperm count. Thus, we can conclude that Kpna6 expression in spermatocytes and round spermatids is essential for normal sperm development and fertility.

### Kpna6 deficiency leads to defects in Sertoli cells

**Fig. 5.**
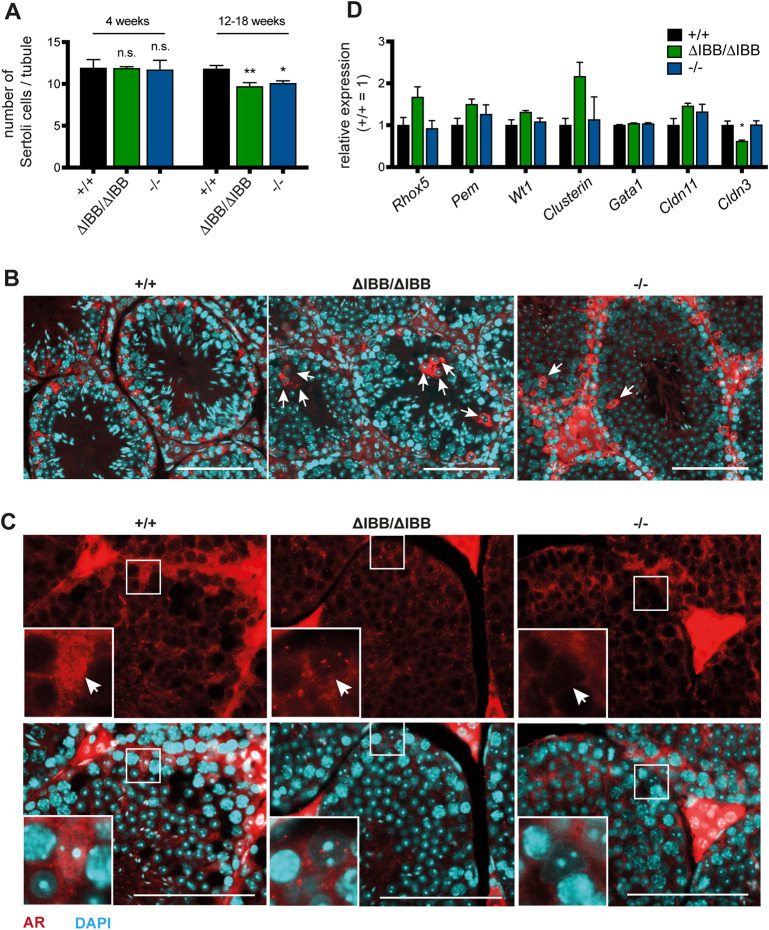
**Kpna6 deficiency decreases number of Sertoli cells and prevents nuclear import of AR.** (A) Number of Sertoli cells per tubule. (B) Aberrant localization of Sertoli cell nuclei in seminiferous tubules of *Kpna6*^ΔIBB/ΔIBB^ and *Kpna6*^−/−^ testes (arrows). (C) Immunofluorescence of AR (red) in testis sections; DAPI (blue). Note the nuclear localization of AR in WT and the empty nuclei in *Kpna6*^ΔIBB/ΔIBB^ and *Kpna6*^−/−^ Sertoli cells (arrows). Insets show higher magnification of the boxed areas. Scale bars: 100 µm. (D) Relative expression levels of selected genes in adult testes determined by quantitative real-time PCR (12-20 weeks; *n*=6 per group).

The intense expression of Kpna6 in the nuclei of WT Sertoli cells indicates that the protein may be essential for the function of these cells. We observed a reduced number of Sertoli cells in testes of adult *Kpna6*^ΔIBB/ΔIBB^ mice and *Kpna6*^−/−^ mice, suggesting that Kpna6 perturbation caused a loss of Sertoli cells (Fig. 5A). Moreover, in both mutant lines Sertoli cells were frequently observed becoming detached in the middle of seminiferous tubules (Fig. 5B). Interestingly, in young prepubertal mice, no differences in Sertoli cell numbers were detected (Fig. 5A), indicating that the proliferation phase of Sertoli cells was not affected.

The androgen receptor (AR), which is highly expressed in Sertoli cells, plays an important role in spermatogenesis. We observed a pronounced reduction of AR in Sertoli cell nuclei of both mutant lines (Fig. 5C). To test whether expression of AR-related or Sertoli cell-specific transcripts is affected in Kpna6-deficient mice, we analyzed expression levels of AR-regulated genes, such as *Rhox5*, *Pem*, *Wt1*, clusterin, *Gata1*, *Cldn3* and *Cldn11* by quantitative real-time PCR. No significant differences in mRNA levels were detected for most of these genes. Only *Cldn3* expression was markedly downregulated in *Kpna6*^ΔIBB/ΔIBB^, but not in *Kpna6*^−/−^, testes (Fig. 5D). To confirm these results, immunostaining of testis sections for CLDN3 was performed, revealing specific localization in basal TJs of late-stage VIII tubules in both mutant mouse lines (Fig. S3A). TJs are a major component of the BTB located between adjacent Sertoli cells. To analyze further the possible defects in TJ formation of Sertoli cells, we performed immunostaining for ZO-1 (Tjp1), a specific TJ protein, in testis sections. No differences were found in its expression and localization in basal TJs and apical ectoplasmic specializations of stage IV-VI tubules (Fig. S3B). We evaluated the functional integrity of the BTB by incubating testicular protein extracts from WT mice with sera taken from WT, *Kpna6*^ΔIBB/ΔIBB^ or *Kpna6*^−/−^ mice at 8, 16 and 20 weeks of age. Western blot analysis revealed, in some cases, differences in the protein band pattern with additional bands appearing in *Kpna6*^ΔIBB/ΔIBB^ and *Kpna6*^−/−^ mice sera. Thus, the immunological barrier is leaky and therefore antibodies against testicular antigens are occasionally present in both mutant lines (Fig. S3C). However, a subsequent analysis of the presence of immunoglobulins within testicular tissue in mice of different ages revealed no differences (Fig. S3D). Moreover, by injection of biotin into the testis we could not find a compromised BTB in *Kpna6*^ΔIBB/ΔIBB^ and *Kpna6*^−/−^ mice (Fig. S3E). These data suggest that the BTB is slightly, but not severely, impaired.

**Fig. 6.**
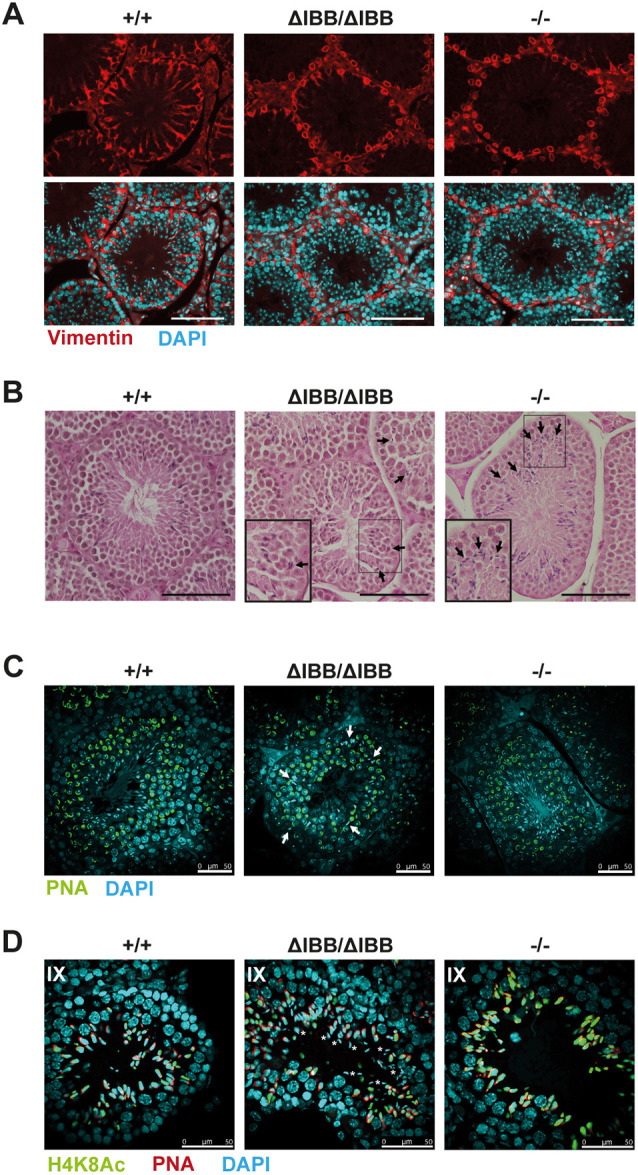
**Morphological and functional abnormalities in Kpna6-deficient Sertoli cells.** (A) Vimentin (red) and DAPI (blue) staining in testis paraffin sections. Scale bars: 100 µm. (B) H&E staining of testis sections showing aberrant sperm orientation in seminiferous tubules of *Kpna6*^ΔIBB/ΔIBB^ and *Kpna6*^−/−^ testes. Arrows mark misoriented sperms. Insets show higher magnification of the boxed areas. Scale bars: 100 µm. (C) DAPI (blue) and PNA (green) staining in testis sections showing defective sperm transport in *Kpna6*^ΔIBB/ΔIBB^ testes. Arrows mark mislocalized sperms. (D) Sperm retention in stage IX seminiferous tubules. Sperm cells were labeled with an antibody against acetyl-histone H4 (H4K8Ac, green), PNA (red) and DAPI (blue). Asterisks indicate sperm retention. Age: 12-16 weeks. Scale bars: 50 µm.

Further analysis of Sertoli cell cytoskeletal proteins revealed an abnormal localization of the intermediate filament vimentin in Sertoli cells of *Kpna6*^ΔIBB/ΔIBB^ and *Kpna6*^−/−^ mice. Vimentin-based filaments no longer stretched across the Sertoli cell cytosol, but instead retracted and were wrapped around the cell nuclei (Fig. 6A). In contrast, beta-III tubulin organization was not perturbed in Sertoli cells of *Kpna6*^ΔIBB/ΔIBB^ and *Kpna6*^−/−^ mice (Fig. S4), suggesting that there is no general effect on Sertoli cell morphology but rather a specific change in vimentin distribution.

The compromised Sertoli cells led to defects in sperm orientation in both *Kpna6*^ΔIBB/ΔIBB^ and *Kpna6*^−/−^ mice. (Fig. 6B). Interestingly, sperm transport through the seminiferous epithelium, which is also dependent on Sertoli cells, was severely disturbed in *Kpna6*^ΔIBB/ΔIBB^ mice, but was found to be unaffected in *Kpna6*^−/−^ mice (Fig. 6C). In WT testis, only one generation of spermatids was found during the transition from round into elongating spermatids. However, two generations of spermatids were often observed in stages IX-XII in *Kpna6*^ΔIBB/ΔIBB^ mice (Fig. 6D). The additional spermatids were more mature with condensed nuclei, implicating that they were not released properly. Although correct spermiation is dependent on Sertoli cells, we could not detect residual sperms in stage IX-XII tubules of *Kpna6*^−/−^ testes, showing that spermatid persistence is not caused by Sertoli cell defects only. Thus, we conclude that absence of Kpna6 leads to disturbed sperm organization in both mutant mouse lines, with more severe appearance in *Kpna6*^−/−^ testes.

### Kpna6 deficiency-related loss of spermatocytes starts with leptotene/zygotene transition

**Fig. 7.**
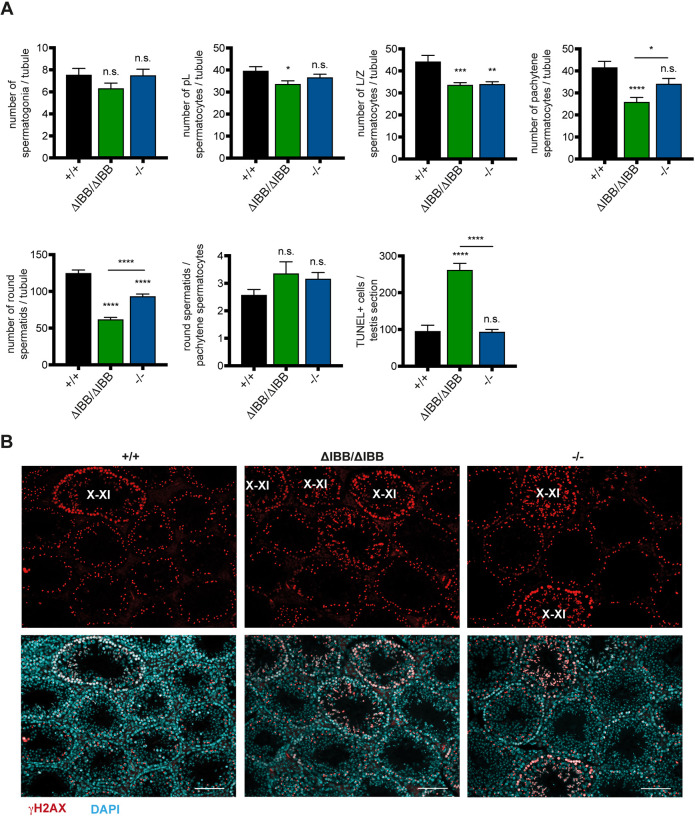
**Kpna6 deficiency results in loss of spermatocytes.** (A) Number of germ cells at different developmental steps per seminiferous tubule in testes of mice aged 12-16 weeks and number of TUNEL-positive cells per testis section (*Kpna6*^+/+^: *n*=6; *Kpna6*^ΔIBB/ΔIBB^: *n*=7; *Kpna6*^−/−^: *n*=8; 12-20 weeks). (B) γH2AX (red) and DAPI (blue) staining in adult testes. X-XI: stage X-XI tubules. Scale bars: 100 µm.

To elucidate the start of germ cell loss in Kpna6-deficient testes, we evaluated the developmental steps of spermatogenesis quantitatively. No differences were detected in germ cells labeled with the pluripotency marker Sall4, excluding a severe loss of spermatogonia (Fig. 7A). We observed that the number of bromodeoxyuridine (BrdU)-labeled preleptotene spermatocytes per tubule in *Kpna6*^ΔIBB/ΔIBB^ were slightly decreased compared with *Kpna6*^−/−^ and WT mice (Fig. 7A). We next tested for γH2AX, a phosphorylated form of histone 2AX, which exhibits an intense diffuse staining pattern in spermatocytes at the leptotene/zygotene transition in stages X-XI, and exclusively localizes to the sex chromosomes within pachytene spermatocytes (Blanco-Rodríguez, 2009; Celeste et al., 2002; Peters et al., 1997). In *Kpna6*^ΔIBB/ΔIBB^ and *Kpna6*^−/−^ testes, the numbers of leptotene/zygotene spermatocytes in stages X-XI decreased markedly compared with WT controls, but we did not observe differences in the stage-specific appearance of γH2AX-positive chromatin (Fig. 7A, B). Interestingly, we detected a further decrease in the number of stage I-VIII pachytene spermatocytes in *Kpna6*^ΔIBB/ΔIBB^ but not *Kpna6*^−/−^ testes, showing that *Kpna6*^ΔIBB/ΔIBB^ were more affected than *Kpna6*^−/−^ testes (Fig. 7A). The ratios of pachytene to leptotene spermatocytes were similar in WT and *Kpna6*^−/−^, whereas this ratio was markedly reduced in *Kpna6*^ΔIBB/ΔIBB^ testes (WT: 0.94; *Kpna6*^ΔIBB/ΔIBB^: 0.77; *Kpna6*^−/−^: 1.00), suggesting that development of pachytene spermatocytes is dependent on Kpna6. The reduced numbers of step 1-8 round spermatids in *Kpna6*^ΔIBB/ΔIBB^ and *Kpna6*^−/−^ testes were comparable with numbers of pachytene spermatocytes, but the ratios of round spermatids to pachytene spermatocytes were similar between all three groups (Fig. 7A). These observations suggest that deficiency of Kpna6 leads to a reduction in leptotene/zygotene spermatocytes, and to a further decrease in pachytene spermatocytes, but surviving spermatocytes can differentiate into round spermatids. The loss of pachytene spermatocytes was accompanied by a higher number of terminal deoxynucleotidyl transferase dUTP nick end labeling (TUNEL)-positive cells in *Kpna6*^ΔIBB/ΔIBB^ testes (Fig. 7A).

### Onset of spermatogenesis is delayed in *Kpna6*^ΔIBB/ΔIBB^ and *Kpna6*^−/−^ mice

**Fig. 8.**
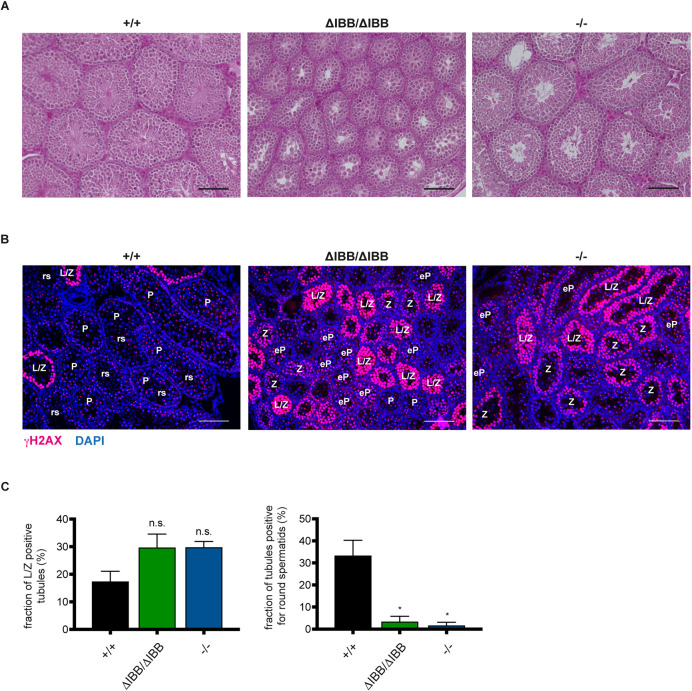
**Kpna6 deficiency causes a delay in the first spermatogenesis wave.** (A) H&E staining of testis sections of WT, *Kpna6*^ΔIBB/ΔIBB^ and *Kpna6*^−/−^ mice at 6 weeks of age showing severely delayed onset of spermatogenesis. Scale bars: 100 µm. (B) γH2AX (red) and DAPI (blue) staining on testis sections of mice aged 21 days. eP, early pachytene spermatocytes; L/Z, leptotene/zygotene spermatocytes; P, pachytene spermatocytes; rs, round spermatids; Z, zygotene spermatocytes. Scale bars: 100 µm. (C) Quantification of tubules containing L/Z spermatocytes and round spermatids, respectively, per section in mice aged 21 days (*Kpna6*^+/+^: *n*=5; *Kpna6*^ΔIBB/ΔIBB^: *n*=5; *Kpna6*^−/−^: *n*=3).

H&E staining of testes from 6-week-old mice revealed a retardation in testis development in *Kpna6*^ΔIBB/ΔIBB^ mice: whereas WT and *Kpna6*^−/−^ mice showed a regular histology with seminiferous tubules at various stages, in *Kpna6*^ΔIBB/ΔIBB^ mice all tubules displayed uniformly the same developmental stage and no round spermatids or later stages could be detected, indicating that the first meiotic wave had not been completed (Fig. 8A). Analysis of meiotic spermatocytes by γH2AX-labeling showed a typical pattern with a short wave of positive nuclei in stage VIII tubules (preleptotene spermotocytes) and a second wave starting in leptotene, which peaked in leptotene/zygotene and decreased into small foci in zygotene before γH2AX labeled the sex chromosomes in early pachytene spermatocytes (Blanco-Rodríguez, 2009; Hamer et al., 2003). Analysis of day 21 testes by γH2AX labeling revealed a high number of leptotene/zygotene spermatocytes in *Kpna6*^ΔIBB/ΔIBB^ mice, whereas in WT testes most of the spermatocytes had already reached pachytene stage and round spermatids started to be present (Fig. 8B,C). Interestingly, *Kpna6*^−/−^ testes also displayed a higher amount of strongly γH2AX-positive leptotene/zygotene spermatocytes and only few round spermatids were visible (Fig. 8B,C). Together, these data show that, compared with WT mice, the onset of the first wave of spermatogenesis is markedly delayed in both *Kpna6*^ΔIBB/ΔIBB^ and *Kpna6*^−/−^ mice, which is consistent with a dysfunction of Sertoli cells in both mouse lines.

### Gene expression changes in *Kpna6*^ΔIBB/ΔIBB^ testis

**Fig. 9.**
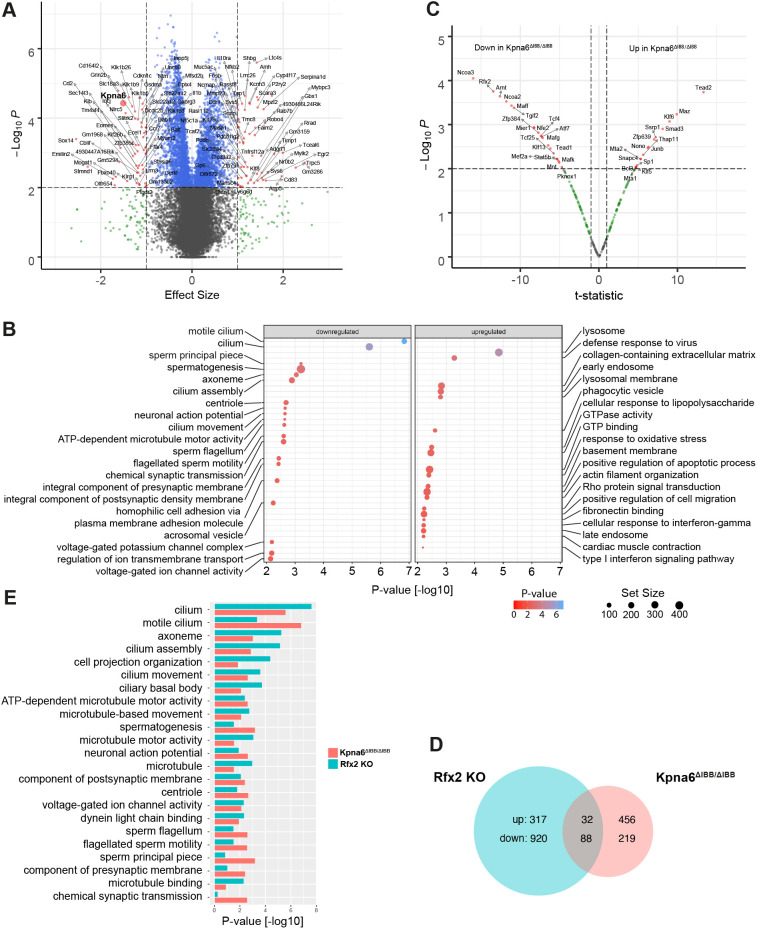
**Downregulated spermiogenesis-related pathways in Kpna6-deficient testis.** (A) Volcano plot depicting the differential gene regulation of the testes transcriptomes of WT and *Kpna6*^ΔIBB/ΔIBB^ mice. The *x*- and *y*-axes show the effect size according to a Wald test and the −log_10_ transformed *P*-value. Small red dots indicate significantly regulated genes according to *P*-value (*P*<0.01) and absolute effect size (>1). The *Kpna6* gene is indicated by an enlarged red circle. (B) Dot plot depicting the 20 most significantly up- and downregulated GO terms in the testes transcriptomes of *Kpna6*^ΔIBB/ΔIBB^ mice compared with WT. The locus and color of the dots indicate the −log_10_ *P*-value, and the dot size is related to the number of genes of the GO term. (C) Volcano plot depicting the predicted differential TF activity of the testis transcriptomes of *Kpna6*^ΔIBB/ΔIBB^ mice compared with WT. The *x*- and *y*-axes show the *t*-statistic of a *t*-test and the −log_10_ transformed *P*-value. Red dots indicate significant differential TF activity according to the *P*-value (*P*<0.01) and the absolute *t*-statistic. (D) Venn diagram showing the commonly and individually up- and downregulated genes from mouse testes in the *Rfx2* knockout (KO) and *Kpna6*^ΔIBB/ΔIBB^ models in comparison with their respective WT controls (*P*-value cutoff<0.01, absolute effect size>0.5). (E) Bar plot depicting the significance of 23 most downregulated GO terms in testes transcriptomes after Kpna6 (red) or RFX2 (green; see Gene Expression Omnibus, accession number GSE68283; Wu et al., 2016) depletion according to GSEA. The bars denote the −log_10_ transformed *P*-values.

To obtain a transcriptome-wide insight into the affected transcripts, pathways and upstream regulators after Kpna6 depletion, we performed RNAseq analysis on whole testes of WT, *Kpna6*^ΔIBB/ΔIBB^ and *Kpna6*^−/−^ mice in triplicate. Principal component analysis (PCA) on the gene-aggregated transcript per million values depicts the transcriptome of *Kpna6*^ΔIBB/ΔIBB^ relative to WT and *Kpna6*^−/−^ mice (Fig. S5A). According to the PCA, the transcriptomes of the former differ the most from the latter two along the first principal component (PC1), which is in accordance with the much more severe phenotype observed in *Kpna6*^ΔIBB/ΔIBB^ compared with *Kpna6*^−/−^ mice. A log likelihood test between the *Kpna6*^ΔIBB/ΔIBB^ and the WT transcriptome revealed 112 significantly regulated genes, with *Kpna6* significantly downregulated in the former (*P*-value: 3×10^−5^; effect size: −1.51; Fig. 9A; Table S1). Contrary to this and in line with the mild phenotype and the PCA, we found only 20 differentially regulated genes between WT and *Kpna6*^−/−^ mice, with *Kpna6* showing no significance in differential expression (Table S1). Therefore, we concentrated our analyses on the comparison of *Kpna6*^ΔIBB/ΔIBB^ and WT testes. We next performed a gene set enrichment analysis (GSEA) based on the gene effect size differences between the WT and *Kpna6*^ΔIBB/ΔIBB^ condition using Gene Ontology (GO) biological processes. Upregulated GO terms in *Kpna6*^ΔIBB/ΔIBB^ were related to cell migration, extracellular matrix and development processes, whereas cilia-, flagellum- and sperm-related terms were downregulated as a result of the dysfunctional *Kpna6* gene (Fig. 9B). To obtain an insight into the upstream transcription factors (TFs), we tested the mouse regulon data from the DoRothEA library against the WT and *Kpna6*^ΔIBB/ΔIBB^ condition. In total, there were 16 and 20 TFs for which putative activity was significantly up- or downregulated, respectively, in *Kpna6*^ΔIBB/ΔIBB^ mice (Fig. 9C). Although the upregulated TF were related to TNFα signaling via NF-κB (adj. *P*-value=0.0008) and TGFβ signaling (adj. *P*-value=0.02), RFX2, a key regulator of mouse spermiogenesis, was downregulated. To test the prediction further, we compared the differential gene regulation from *Kpna6*^ΔIBB/ΔIBB^ mice with testicular transcriptomes of *Rfx2* knockout mice (Wu et al., 2016). The effect size and the direction of differential gene regulation for *Rfx2* knockout and *Kpna6*^ΔIBB/ΔIBB^ mice relative to their wild-type conditions were significantly correlated according to Spearman's ρ statistic (*P*-value<2.2×10^−16^). Although both mouse lines share few upregulated genes (10% of *Rfx2* knockout; 7% of *Kpna6*^ΔIBB/ΔIBB)^, 40% of the genes that were downregulated in *Kpna6*^ΔIBB/ΔIBB^, were also downregulated in the *Rfx2* knockout (Fig. 9D; Table S2). GSEA of the downregulated pathways in *Rfx2* knockout and *Kpna6*^ΔIBB/ΔIBB^ mice revealed similar effects on cilia, assembly of cilia, and microtubule- and sperm motility-related processes (Fig. 9E; Table S3). However, RFX2 localization in the testis did not show abnormalities in *Kpna6*^ΔIBB/ΔIBB^, being strongly expressed in nuclei of step 2-3 round spermatids, whereas the protein localized to a distinct spot in the nuclei of step 4-8 round spermatids before disappearing at the start of elongation (Fig. S5B). These data indicate that RFX2 is normally expressed in *Kpna6*^ΔIBB/ΔIBB^ mice, but its activity on target genes seems to be impaired in the absence of Kpna6.

### Downregulation of protamines and transition proteins in round spermatids and impaired histone-protamine exchange in *Kpna6*^ΔIBB/ΔIBB^ mice

**Fig. 10.**
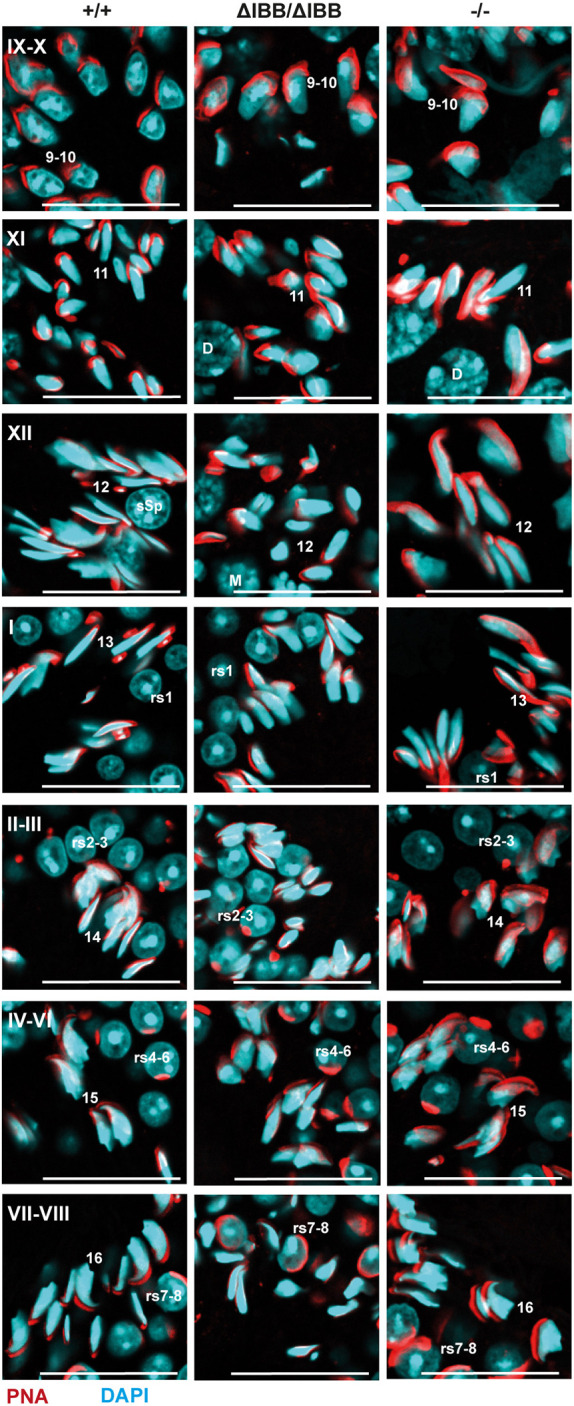
**Kpna6-deficient spermatids show abnormal elongation and nuclear shaping.** Morphology of DAPI- (blue) and PNA-labeled (red) spermatids throughout their development. The deficiency/delay in elongation can be seen already in stage XII tubules. Roman numbers mark tubular stages, Arabic numbers indicate the developmental step of spermatids. Note the abnormal configuration of sperm heads in *Kpna6*^ΔIBB/ΔIBB^ spermatids. Age of mice: 12 weeks. Scale bars: 25 µm.

**Fig. 11.**
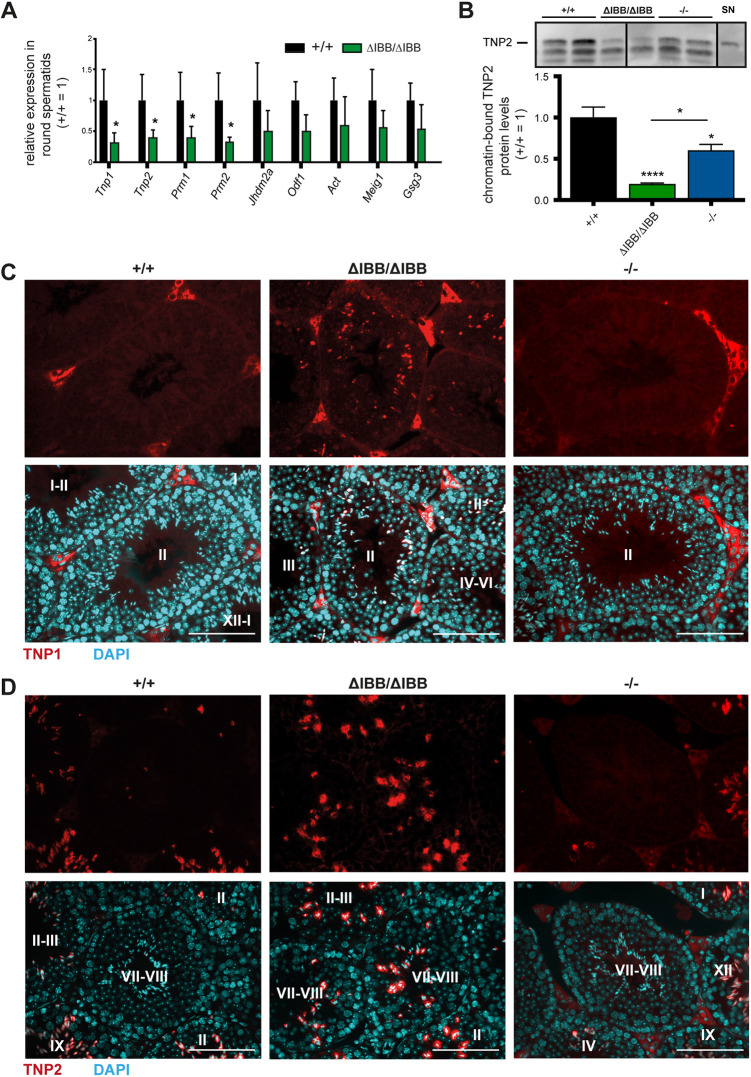
**Reduced expression of transition nuclear proteins and protamines in Kpna6-deficient testes.** (A) Quantitative real-time PCR analysis of various postmeiotic genes in isolated round spermatids of adult (12-20 weeks) WT and *Kpna6*^ΔIBB/ΔIBB^ testes (*Kpna6*^+/+^: *n*=6; *Kpna6*^ΔIBB/ΔIBB^: *n*=9). (B) Western blot analysis and quantification of TNP2 in WT *Kpna6*^ΔIBB/ΔIBB^ and *Kpna6*^−/−^ testis chromatin extracts. SN, non-chromatin-associated TNP2 served as control. (C,D) TNP1 (C, red), TNP2 (D, red) and DAPI (blue) staining on sections of adult WT, *Kpna6*^ΔIBB/ΔIBB^ and *Kpna6*^−/−^ testes. Roman numbers mark the stage of seminiferous tubules. Scale bars: 100 µm.

Because analysis of the altered gene expression in *Kpna6*^ΔIBB/ΔIBB^ mice suggested the strongest impact on postmeiotic events, we performed a detailed microscopic study of sperm maturation in the testis. Although spermatid elongation started regularly in stage IX seminiferous tubules of *Kpna6*^ΔIBB/ΔIBB^ mice, the subsequent steps were characterized by abnormal nuclear shaping of elongating spermatids and mature step 15-16 sperms were absent in *Kpna6*^ΔIBB/ΔIBB^ mice (Fig. 10). Together with the reduced number of spermatozoa and abnormal morphology of epididymal sperms in *Kpna6*^ΔIBB/ΔIBB^ mice, this suggested that spermiogenesis was severely affected by Kpna6 deficiency, confirming the results of gene expression analysis. We next tested whether the regulation of postmeiotic factors, which are necessary for sperm maturation, is mediated by Kpna6. The analysis of gene expression in whole testis had already shown a slight downregulation of *Tnp1*, *Tnp2*, *Prm1* and *Prm2* (respective adj. *P*-values: 0.007, 0.06, 0.06, 0.02; Table S1). Real-time PCR analysis demonstrated that the mRNAs of these genes were significantly reduced in FACS-sorted isolated round spermatids of *Kpna6*^ΔIBB/ΔIBB^ mice compared with WT and *Kpna6*^−/−^ (Fig. 11A; Fig. S6A,B). In contrast, other postmeiotic key genes were not significantly affected in *Kpna6*^ΔIBB/ΔIBB^ mice (Fig. 11A). Moreover, western blotting of chromatin-bound proteins showed that TNP2 was drastically decreased in the testes of *Kpna6*^ΔIBB/ΔIBB^ mice (Fig. 11B). Although TNP1 and TNP2 were correctly localized in elongating spermatids starting at step 9 (Fig. S6C,D), we found a prolonged persistence of these two proteins in *Kpna6*^ΔIBB/ΔIBB^ spermatids. In WT and *Kpna6*^−/−^ testes, TNP1 could not be found in tubules later than stage I, and TNP2 was not expressed past stage III; however, we consistently found TNP1-positive sperms until stage III tubules und expression of TNP2 was even found in sperms of stage VIII (and IX, residual sperms) tubules (Fig. 11C,D). Taken together, these data show that the expression of protamines and transition proteins is markedly reduced and that protamine-histone exchange is severely disturbed in mice lacking Kpna6.

### Analysis of chromatin remodeling and presence of transcription factors in *Kpna6*^ΔIBB/ΔIBB^ mice

Next, we investigated the tremendous remodeling of chromatin that takes place during spermatid development, as this process is dependent on proteins that enter the nucleus of round and elongating spermatids. Analysis of histone H3 2- and 3-methylation as well as hyperacetylation of histone H3 (K9 and K14) and H4 (K8 and K12) did not reveal any differences of *Kpna6*^ΔIBB/ΔIBB^ and WT spermatids. Moreover, we observed regular appearance of DNA double-strand breaks detected by γH2AX labeling (Fig. 7B).

The significant reduction in gene expression of *Tnp1*, *Tnp2*, *Prm1* and *Prm2* may result from impaired expression or nuclear translocation of TFs. In mice, the transcriptional activation of these genes is mainly regulated by CREM (Mali et al., 1989; Nantel et al., 1996). WT testis showed a normal expression and localization of CREM in round spermatids step 2-8 (Fig. S7) and with onset of elongation the CREM expression started to decline. However, no major changes in CREM expression and localization could be observed in *Kpna6*^ΔIBB/ΔIBB^ testis (Fig. S6), suggesting that nuclear import of CREM is unaffected.

**Fig. 12.**
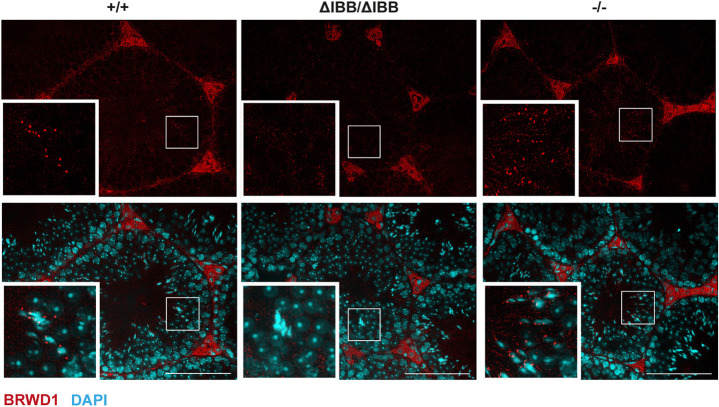
**Disturbed localization of BRWD1 in *Kpna6*^−/−^ testes.** Immunofluorescence for BRWD1 (red) in WT, *Kpna6*^ΔIBB/ΔIBB^ and *Kpna6*^−/−^ testis on paraffin sections, counterstained with DAPI (blue). Age of mice: 12-16 weeks. Insets show higher magnifications of the boxed regions. Scale bars: 100 µm.

The transcriptional regulator BRWD1 has been shown to be essential for spermiogenesis (Pattabiraman et al., 2015). Being part of a postmeiotic transcriptional activator complex, BRWD1 binds to acetylated lysine residues of histones, causing transcriptional activation. Immunofluorescence of BRWD1 revealed striking differences in its expression in *Kpna6*^ΔIBB/ΔIBB^ testes compared with WT and *Kpna6*^−/−^ testes. Whereas in WT and *Kpna6*^−/−^ testes, positive signals localized to spots in the cytoplasm could be found in paraffin-embedded testis sections in step 9 elongating spermatids and in stage IV-VI tubules, no BRWD1 signal was found in *Kpna6*^ΔIBB/ΔIBB^ testis (Fig. 12). As the signals tended to be very subtle in paraffin sections, immunostaining was repeated in snap-frozen sections, confirming very clearly the spotty pattern of expression of BRWD1 in WT and *Kpna6*^−/−^, but not in *Kpna6*^ΔIBB/ΔIBB^, testes (Fig. S8A). As quantification of BRWD1 by western blotting did not show a significant difference in whole-testis extracts (Fig. S8B), we suggest that the intracellular localization of the protein is affected by the absence of Kpna6.

## DISCUSSION

We used two different knockout mouse models either lacking functional Kpna6 in all cells of the testis (*Kpna6*^ΔIBB/ΔIBB^) or expressing it only in germ cells (*Kpna6*^−/−^) to analyze the biological function of Kpna6 during spermatogenesis and showed for the first time that Kpna6 in germ cells is essential for mammalian male fertility. These findings are in accordance with the phenotype of fruit flies lacking karyopherin α1, which is one (of three) karyopherin α paralogs in *Drosophila melanogaster* with the highest similarity to mouse Kpna6. *D. melanogaster* karyopherin α1 is essential for spermatogenesis and its depletion leads to full arrest with spermatocytes exhibiting abnormal nuclear shape (Ratan et al., 2008). However, the molecular mechanisms by which *D. melanogaster* karyopherin α1 exerts its effects on male fertility have not been assessed yet.

Mouse *Kpna6* mRNA is expressed in isolated pachytene spermatocytes and round spermatids (Holt et al., 2007; Major et al., 2011; Shima et al., 2004), and it is upregulated during the first spermatogenesis wave: a first increase takes place from postnatal day 10 to day 20, when spermatocytes progress into late pachytene stage; and a second increase occurs from postnatal day 20 to day 30, when round spermatids become enriched (Major et al., 2011; Namekawa et al., 2006). The Kpna6 protein expression pattern during spermatogenesis detected by immunohistochemistry in our current study is consistent with these data. Additionally, microarray data had previously shown that *Kpna6* mRNA is moderately expressed in cultured Sertoli cells isolated from postnatal day 16 to day 18 testes (Holt et al., 2007; Shima et al., 2004), whereas in adult testis, *Kpna6* mRNA was not detected in Sertoli cells by *in situ* hybridization (Hogarth et al., 2006). However, our present study shows that Kpna6 is also highly expressed in the nuclei of Sertoli cells throughout all stages of the seminiferous epithelium in the adult testis.

Studies of other karyopherin α isoforms in adult mouse testis have shown stage- and cell type-specific expression of Kpna2, Kpna3 and Kpna4 (Miyamoto et al., 2012). Our own comprehensive studies revealed that Kpna6 is the only karyopherin α isoform expressed in elongating spermatids. Moreover, we detected a massive increase in Kpna6 expression in late round and early elongating spermatids that has not been seen for any other karyopherin α subtype. The infertility phenotype of Kpna6-deficient mice underlines the hypothesis that this protein has unique functions in spermatogenesis, and that other karyopherin α subtypes cannot compensate for its absence.

One of the main findings of our present work is that Kpna6 has two different functions during spermatogenesis depending on its two localizations in Sertoli cells and in developing sperms. The use of two different Kpna6-deficient mouse lines with distinct expression patterns in these cell types allowed us to discriminate between these two functions as both lines display distinct phenotypes. The *Kpna6*^ΔIBB/ΔIBB^ mice are infertile and are characterized by the absence of the full-length protein in Sertoli cells and developing sperms. The *Kpna6*^−/−^ mice are fertile and produce sperms that can be clearly attributed to a rescued expression of the protein in developing sperms based on a germ cell-specific promoter in the *Kpna6* gene. The utilization of a germ cell-specific promoter is a well-known mechanism and has been shown for a variety of transcripts encoding for proteins such as angiotensin converting enzyme, c-abl (Abl1), pro-opiomelanocortin and β-galactosyltransferase (Hecht, 1998). In WT and *Kpna6*^−/−^ mice, germ cells produce *Kpna6* mRNA by using an alternative exon 1A with a germ cell-specific 5′-untranslated region, suggesting differential transcriptional control mechanisms for *Kpna6* in these cells. However, the absence of Kpna6 in Sertoli cells seems to account for a partially reduced sperm count. This finding was confirmed by intercrossing of both lines, producing compound heterozygous offspring, which also displayed a reduced sperm count but were fertile. This provides clear evidence that the partial sperm count reduction can be attributed to the Sertoli cell-related phenotype, as the compound heterozygous mice represent a rescue of the infertile *Kpna6*^ΔIBB/ΔIBB^ mice with expression of the protein in germ cells, but not in Sertoli cells.

We revealed several alterations in Kpna6-deficient Sertoli cells that may explain this effect. First, we observed reduced nuclear AR in Sertoli cells of both mutant mouse lines. It has been shown that ligand-dependent nuclear import is crucial for the function of AR (Becker et al., 2000; Kawate et al., 2005; Nakauchi et al., 2007; Thomas et al., 2004), and its nuclear import is karyopherin α/β dependent (Cutress et al., 2008; Kaku et al., 2008). Mice with Sertoli cell-specific ablation of the AR (SCARKO) exhibit defective Sertoli cell polarization and nuclear position in the tubules, and progress through meiosis is disturbed with increased rate of apoptosis (Meng et al., 2011; Meng et al., 2005; Willems et al., 2010). Increased permeability of the BTB and downregulation of *Cldn3* are typically found in SCARKO mice. Similar alterations were found in *Kpna6*^ΔIBB/ΔIBB^ mice and partly in *Kpna6*^−/−^ animals, but the effects were less pronounced. Most AR-dependent genes were normally expressed and the observed decrease in *Cldn3* mRNA levels in *Kpna6*^ΔIBB/ΔIBB^ mice may be at least partially due to the loss of germ cells, in which this gene is also expressed (Chihara et al., 2013). Second, we found that Sertoli cells of both mutant lines display abnormal organization of the intermediate filament vimentin. Whereas vimentin is stretched across the Sertoli cell cytosol in WT testis, both mutant mouse lines showed a concentration around the Sertoli cell nucleus with no extensions. Similar findings have been found in mice with a defective cytoplasmic dynein 1 heavy chain and in mice that have been depleted of RAPTOR, a key component of mTORC1 (Wen et al., 2018; Xiong et al., 2018). However, in both of these mouse lines, the phenotype included a severe disorganization of actin and microtubules, which we could not detect, excluding major morphological changes of Sertoli cell cytoplasm, which still stretched out into the tubular lumen.

Despite these relatively minor alterations in Sertoli cells lacking Kpna6, the dynamic remodeling of the BTB during stages VII and VIII, which allows preleptotene spermatocytes passage through the barrier (Bremner et al., 1994; Dym and Fawcett, 1970), seems to be impaired in *Kpna6*^ΔIBB/ΔIBB^ and *Kpna6*^−/−^ testes. We observed a relative reduction in leptotene/zygotene spermatocytes of about 25% in both lines, which would be consistent with a meiotic delay due to a prolonged leptotene/zygotene phase as it has been observed in *Cldn3* knockdown mice (Chihara et al., 2013). Although both mutant mouse lines exhibit a loss of leptotene/zygotene spermatocytes, only *Kpna6*^ΔIBB/ΔIBB^ mice show an even more pronounced loss of pachytene spermatocytes and round spermatids. Because in pachytene spermatocytes, in contrast to leptotene/zygotene spermatocytes, *Kpna6* mRNA is already detectable (Holt et al., 2007; Major et al., 2011; Shima et al., 2004), low levels of protein might be crucial from this developmental stage on, explaining the loss of these postmeiotic cell types.

The retardation in the first wave of spermatogenesis was confirmed by detailed analyses in 21-day-old testes. This delay fits with previous reports of Kpna6 being upregulated from postnatal day 10, suggesting a very specific role at this time point (Major et al., 2011; Namekawa et al., 2006). Given that both mouse lines are affected, we conclude that the defects in Sertoli cell function are responsible for this phenotype.

Multiple molecular events have to occur for a round spermatid to become a mature sperm. These events include chromatin condensation, reorganization of the spermatid nucleus, formation of an acrosome and assembly of a sperm tail (Sassone-Corsi, 2002). It has been shown that a number of postmeiotic proteins, including TNP1 and TNP2, PRM1 and PRM2, MEiG1, ODF1, JHDM2A (KDM3A), ACT and CAPZA3 (also known as GSG3), are important for spermiogenesis (Geyer et al., 2009; Kotaja et al., 2004; Liu et al., 2010; Okada et al., 2007; Salzberg et al., 2010; Yang et al., 2012; Zhang et al., 2009). The transition from round spermatids to mature spermatozoa was severely affected in *Kpna6*^ΔIBB/ΔIBB^ mice. Spermiogenesis requires extensive chromatin condensation, which is achieved by replacement of histones by TNP1 and TNP2 and, subsequently, by PRM1 and PRM2. Accordingly, the genetic ablation of transition proteins or protamines causes defective spermiogenesis (Cho et al., 2001; Yu et al., 2000; Zhao et al., 2004; Zhao et al., 2001) comparable to the phenotype of *Kpna6*^ΔIBB/ΔIBB^ males. By RNAseq, we found that Kpna6 deficiency reduced *Tnp1*, *Tnp2*, *Prm1* and *Prm2* expression in the testis. As the analysis of whole-testis RNA bears the risk of different cellularity between the three mouse lines, we extended our study using FACS-sorted round spermatids and found that the reduced expression of *Tnp1*, *Tnp2*, *Prm1* and *Prm2* was even more pronounced. Moreover, total chromatin-bound TNP2 was markedly reduced in testes of *Kpna6*^ΔIBB/ΔIBB^ mice as assessed by western blotting. In addition, we detected a prolonged presence of transition proteins in spermatids of *Kpna6*^ΔIBB/ΔIBB^ mice, suggesting that Kpna6 is not only essential for the expression of TNPs and PRMs but also for histone/protamine exchange.

The transcriptional regulator BRWD1 has been shown to be crucial for spermiogenesis (Pattabiraman et al., 2015). Although a distinct pathway has not been found yet, the reduced transcription of postmeiotic genes, including *Tnp1*, *Tnp2*, *Prm1* and *Prm2*, in *Brwd1*-defective testes has been suggested to cause male infertility. By immunohistochemistry, we found a spotty pattern of BRWD1 localization in WT and *Kpna6*^−/−^ testes, which was absent in *Kpna6*^ΔIBB/ΔIBB^ testes, although the amount of the protein was indistinguishable between the strains. These data render BRWD1 a possible mediator of the observed impairments in transition protein and protamine expression in *Kpna6*^ΔIBB/ΔIBB^ testes.

GSEA of the RNAseq data clearly indicated a downregulation of cilium- and sperm motility-related processes in *Kpna6*^ΔIBB/ΔIBB^ mice, corroborating the finding of defective spermiogenesis. Analysis of putative TFs singled out a downregulation of RFX2 gene targets. Although *Rfx2* itself was not differentially regulated, its targets related to cilium, axoneme, microtubule and sperm motility were, which was evident from a comparison of our RNAseq data to a testis transcriptome of *Rfx2* knockout mice (Wu et al., 2016). Although immunohistochemical analysis of RFX2 in testis did not reveal a differential localization of the protein, it might be possible that its post-translational modification is disturbed or that a co-factor that has yet to be determined is needed for effective transcription.

In the present study, we have shown for the first time that Kpna6 is essential for mammalian male fertility. We revealed two cell types in which the protein is functionally important in the testis. In Sertoli cells, its deficiency causes alterations in AR and vimentin localization, and in BTB dynamics, leading to a delay in spermiogenesis. At the level of round spermatids Kpna6 is the main karyopherin α isoform expressed and its deficiency severely affects the activity of RFX2, the localization of BRWD1, and the expression of transition proteins and protamines as well as the histone/protamine exchange. These effects contribute to the massive loss of elongating spermatids and the complete male infertility observed in *Kpna6*^ΔIBB/ΔIBB^ mice. However, further investigations will need to be performed to identify all the cellular and molecular mechanisms involved in the complex phenotype of infertility observed in Kpna6-deficient mice.

## MATERIALS AND METHODS

### Animals

*Kpna6*^−/−^ and *Kpna6*^ΔIBB/ΔIBB^ mice were generated as described previously (Rother et al., 2011). Animals were backcrossed for ten generations to C57Bl6/N background. All experiments were performed according to national and institutional guidelines and were approved by the relevant authority (Landesamt für Gesundheit und Soziales Berlin, Germany).

### Western blot

Mouse testes were collected and homogenized in RIPA-Buffer supplemented with protease inhibitor cocktail (Sigma-Aldrich). Following sonication and centrifugation (10 min at 16,000 ***g***), protein concentrations in the tissue extracts were measured using bicinchoninic acid solution/copper sulfate solution 50:1 (Sigma-Aldrich) and 40 µg of total protein was separated by SDS-PAGE. After the transfer of proteins, the PVDF membrane was blocked with Odyssey blocking solution (LI-COR) and subsequently incubated with primary antibodies at 4°C overnight. The following day, the membrane was incubated with an IRDye-coupled secondary antibody for 1 h at room temperature and detection was performed using an Odyssey Infrared Scanner (LI-COR). Signals were quantified using Odyssey Infrared Scanner software (LI-COR). The generation of C-terminal and N-terminal antibodies against Kpna6 was accomplished using standard protocols and has been described previously (Köhler et al., 1999). A complete list of antibodies and conditions is provided in Table S4. For quantifications, at least three independent experiments were performed.

### RNA isolation, reverse transcription and PCR

Tissue was homogenized in Trizol and extracted with chloroform, then precipitated in isopropanol and washed with 70% ethanol. The pellet was dried and resuspended in DEPC-treated water. RNA was then digested with DNAse I, and 2 µg of digested RNA was subjected to reverse transcription using a standard protocol. PCR was performed using 10 ng of cDNA; primers are listed in Table S5.

### Testosterone measurement

Blood samples were collected from 10-week-old mice by cardiac puncture. Serum samples were prepared as described previously (Jeyaraj et al., 2005). The concentrations of testosterone in the serum samples were measured by using a Testosterone EIA kit (Cayman Chemical Company).

### Histological analysis

Testes and epididymides were fixed in 4% neutral-buffered formalin. After fixation, tissues were dehydrated in increasing concentrations of ethanol, embedded in paraffin wax, and sectioned at a thickness of 5 µm. Sections were deparaffinized, rehydrated and stained with H&E according to standard protocols. For quantification of diameters of seminiferous tubules, images of H&E-stained testis sections (five animals per group) were taken using a Keyence microscope (Keyence, Bioreva BZ-9000) and analyzed; 30 tubules per animal were measured using ImageJ software.

### Epididymal sperm count

Sperm count was performed as described previously (Liu et al., 2010; Wu et al., 2000). Briefly, one caudal epididymis was used for histological examination, and the other was minced in 1 ml of PBS. Sperms were allowed to disperse into solution by incubating for 5 min at room temperature. An aliquot of the sperm/saline mixture was then counted in a hemocytometer. The hemocytometer count was multiplied by appropriate volume and dilution factors to give a total cauda epididymal sperm count.

### Immunohistochemical analysis

For IF staining, sections underwent deparaffination followed by rehydration and antigen retrieval using either citrate buffer pH 6 or Tris-EDTA buffer pH 9 for 20 min, as appropriate. The sections were then treated with 10% normal donkey serum for 1 h at room temperature and subsequently incubated with primary antibody overnight at 4°C. The following day, sections were washed with PBS, incubated with secondary antibody for 2 h at room temperature, washed again with PBS and incubated for 1 h at room temperature with peanut agglutinin (PNA), subsequently washed again and embedded in mounting medium containing DAPI (Vectashield, Vector Laboratories/Biozol). Whenever needed, the immunostaining was performed on 10 µm-thick frozen sections of testis fixed in 4% neutral-buffered formalin. A complete list of antibodies and conditions is provided in Table S4. Images of stained tissue sections were taken using a fluorescence microscope (Keyence, Bioreva BZ-9000) or a confocal fluorescence microscope (Leica TCS SPE). For cell counts, at least 100 seminiferous tubules of three to eight mice per group were analyzed using ImageJ software.

### Counting of developmental steps of germ cells

Spermatogonia were counted based on their positive staining for Sall4; the histogram shows the counts for BrdU-labeled S-phase preleptotene spermatocytes (only stage VII and VIII tubuli were used for calculation). For counting of leptotene/zygotene spermatocytes, intense expression of γH2AX was used as a marker. Pachytene spermatocytes were counted based on their nuclear shape and chromatin (DAPI) staining and their position in the tubuli.

### Determination of apoptosis by TUNEL staining

Apoptosis in tissue sections of mouse testis was analyzed by TUNEL staining using an *in situ* cell death detection kit-Fluorescein (11684795910, Merck). In brief, paraffin-embedded testis sections were deparaffinized and rehydrated, and antigen retrieval was performed using citrate buffer pH 6.0 for 10 min. Nonspecific binding was blocked using 10% normal donkey serum for 30 min. The sections were then incubated for 90 min at 37°C with TUNEL reaction mixture prepared according to the manufacturer's protocol. The sections were washed in 1× PBS and incubated with PNA for 1 h at room temperature, then washed and coverslipped with mounting medium containing DAPI. The slides were visualized under a Keyence microscope (Keyence, Bioreva BZ-9000). The number of apoptotic cells was counted per entire section using ImageJ software.

### Quantitative real-time PCR

Total RNA was extracted from WT, *Kpna6*^−/−^ and *Kpna6*^ΔIBB/ΔIBB^ testes and FACS-sorted germ cells using RNeasy Mini Kits (Qiagen). First-strand DNA synthesis was performed using M-MLV Reverse Transcriptase (Invitrogen) and random primers according to the manufacturer's instructions. Quantitative PCR was performed using GoTaq (Promega) on an IQ 5 Multicolour Realtime PCR Detection System (Bio-Rad). Relative gene expression was calculated using the ΔΔCt method with *Gapdh* as normalizing gene. Primer sequences are listed in Table S5.

### Autoantibody detection

Autoantibodies against sperm proteins were detected as described previously (Meng et al., 2011). Briefly, blots with testicular proteins of 2-month-old WT mice were incubated with a 1:50 dilution of either WT, *Kpna6*^ΔIBB/ΔIBB^ or *Kpna6*^−/−^ mutant sera overnight at 4°C. Primary antibodies were detected with an IRDye coupled secondary anti-mouse antibody for 1 h at room temperature and detection was performed using the Odyssey Infrared Scanner (LI-COR).

### Biotin-labeling of the BTB

Mice were sacrificed by cervical dislocation and testes were carefully pulled out of the body without extracting them, then 50 µl of 1 mM CaCl_2_ containing 10 mg/ml biotin (EZ-Link Sulfo-NHS-LC-Biotin, Pierce) were injected with a 0.4 mm needle into one testis. As a control, the second testis was injected with 50 µl of 1 mM CaCl_2_ only. After 30 min of distribution of the injected solution via diffusion, the testes were dissected and snap-frozen in Tissue-Tek OCT compound (Sakura Finetek). Cryoslices were cut at 15 µm thickness, mounted on glass slides and fixed with 4% paraformaldehyde for 15 min. After washing, the sections were incubated with streptavidin-Cy5 directly, coverslipped and observed under a fluorescence microscope (Keyence, Bioreva BZ-9000).

### BrdU injection

To analyze proliferation of germ cells, animals received two intraperitoneal injections of BrdU (50 mg/kg body weight dissolved in 0.9% NaCl; Sigma-Aldrich) 2 h apart and were sacrificed 2 h after the second injection.

### RNA sequencing

A total amount of 1 μg RNA per sample was used as input material for the RNA sample preparations. Sequencing libraries were generated using NEBNext UltraTM RNA Library Prep Kit for Illumina (NEB) following the manufacturer's recommendations and index codes were added to attribute sequences to each sample. Briefly, mRNA was purified from total RNA using poly-T oligo-attached magnetic beads. Fragmentation was carried out using divalent cations under elevated temperature in NEBNext First Strand Synthesis Reaction Buffer (5×). First strand cDNA was synthesized using random hexamer primer and M-MuLV Reverse Transcriptase (RNase H). Second strand cDNA synthesis was subsequently performed using DNA Polymerase I and RNase H. Remaining overhangs were converted into blunt ends by exonuclease/polymerase activities. After adenylation of 3′ ends of DNA fragments, NEBNext Adaptors with hairpin loop structure were ligated to prepare for hybridization. In order to select cDNA fragments of preferentially ∼150-200 bp in length, the library fragments were purified with the AMPure XP system (Beckman Coulter). Then, 3 μl USER Enzyme (NEB) was used with size-selected, adaptor-ligated cDNA at 37°C for 15 min followed by 5 min at 95°C before PCR. PCR was performed with Phusion High-Fidelity DNA polymerase, Universal PCR primers and Index (X) Primer. Finally, PCR products were purified (AMPure XP system) and library quality was assessed on the Agilent Bioanalyzer 2100 system. Fastq reads were pseudo-aligned to the mm10 genome assembly using kallisto (version 0.46) and transcript read counts were aggregated to Ensembl Gene IDs for further analysis. Differential gene expression analysis was performed using the R library sleuth (Pimentel et al., 2017). Significance and effect sizes of differential gene regulation were calculated from the likelihood ratio and the Wald test, respectively, as implemented in the sleuth package. GO term and pathway enrichment analyses were performed based on the effect size between the WT and knockdown strains using the generally applicable GSEA GAGE, which determines whether a set of genes is systematically up- or downregulated as a whole (Luo et al., 2009). For gene set definitions, we used the Molecular Signatures Database (MSigDB) from the R msigdf package (Version 7.1) (Liberzon et al., 2015). Gene sets with fewer than three or those with more than 500 members were discarded for statistical robustness and biological interpretation. Putative TF activity from RNAseq data was assessed per pseudo time point against healthy controls using the mouse gene set resource DoRothEA v1, which provides a curated collection of TF and target gene interactions (the regulon) from different sources (Garcia-Alonso et al., 2019). Only interactions with high, likely and medium confidence (levels A, B, C) were considered. Regulons were statistically evaluated using the R package viper (v1.22.0; row-wise *t*-tests) (Alvarez et al., 2016). Only regulons having at least 15 expressed gene targets were considered.

### Testicular single-cell suspensions

Cells were isolated according to the protocol of Getun et al. (2011) with slight modifications. Briefly, the tunica albuginea was removed, and the seminiferous tubules were fragmented with scissors. The fragments were dissociated in dispase (BD Biosciences) with 10 U/ml DNAse I for 40 min at 37°C. After centrifugation for 3 min at 2500 ***g***, the pelleted tubules were resuspended in TrypLE Express Enzyme (Life Technologies) with 10 U/ml DNAse I and incubated at 32°C for 20 min. The resulting whole-cell suspension was successively washed with Gey's balanced salt solution (GBSS, Sigma-Aldrich). Then, the cell pellet was resuspended in GBSS supplemented with 10% fetal calf serum and 10 U/ml DNAse I. The dissociated testis sample was then passed through a 40 µm GBSS pre-wetted disposable cell strainer. Final staining was performed by adding Hoechst 33342 (5 µg/ml; Thermo Fisher Scientific) to the dissociated testis sample and incubating at 32°C for 1 h. Before analysis, propidium iodide (PI; 2 µg/ml; Thermo Fisher Scientific) was added to exclude dead cells.

### FACS sorting

FACS sorting was performed according to a slightly modified protocol of Bastos et al. (2005). Briefly, the enrichment of round spermatids was performed on a FACSAria III cell sorter from BD Biosciences. Live stained testicular cells were excited with a near UV laser (375 nm), the two parameters Hoechst blue (450/40 BP) and Hoechst red (670 LP; used on Hoechst 33342-stained cells) were used to identify and sort. A sample of every sorting event was assessed for purity of round spermatids under a fluorescence microscope.

### Extraction of chromatin-bound proteins

Extraction of basic nuclear proteins from mouse testis was performed according to Eckhardt and Wang-Eckhardt (2015). Briefly, one testis was homogenized in ice-cold NETN buffer, centrifuged at 12,000 ***g*** for 10 min, resuspended in NETN buffer and centrifuged again. Then, the pellet was resuspended in 0.2 N HCl and incubated overnight at 4°C. After centrifugation at 12,000 ***g*** for 10 min, the supernatant containing basic nuclear proteins was neutralized with 1 M Tris-HCl (pH 8.5) and protein concentration was determined.

### Statistics

Statistical analysis was performed with Prism7 (GraphPad). Results are presented as mean±s.e.m. Significance was determined by using ANOVA (where three groups were compared) or the unpaired two-tailed Student's *t*-test. For distribution of the genotype after heterozygous mating, the binominal test was used. Given *n* numbers in figure legends represent biological replicates. Significance was assumed for *P*<0.05 (*P<0.05; **P<0.01; ***P<0.001; ****P<0.0001; n.s., not significant).

## Supplementary Material

Supplementary information

Reviewer comments
