# Supplementary material for: Kpna6 deficiency causes infertility in male mice by disrupting spermatogenesis
Source: Development. 2021 Oct 4;148(19):dev198374. doi: 10.1242/dev.198374 (PMC8513612; doi:10.1242/dev.198374)
Supplement: Supplementary information [file develop-148-198374-s1.pdf]

Fig. S1

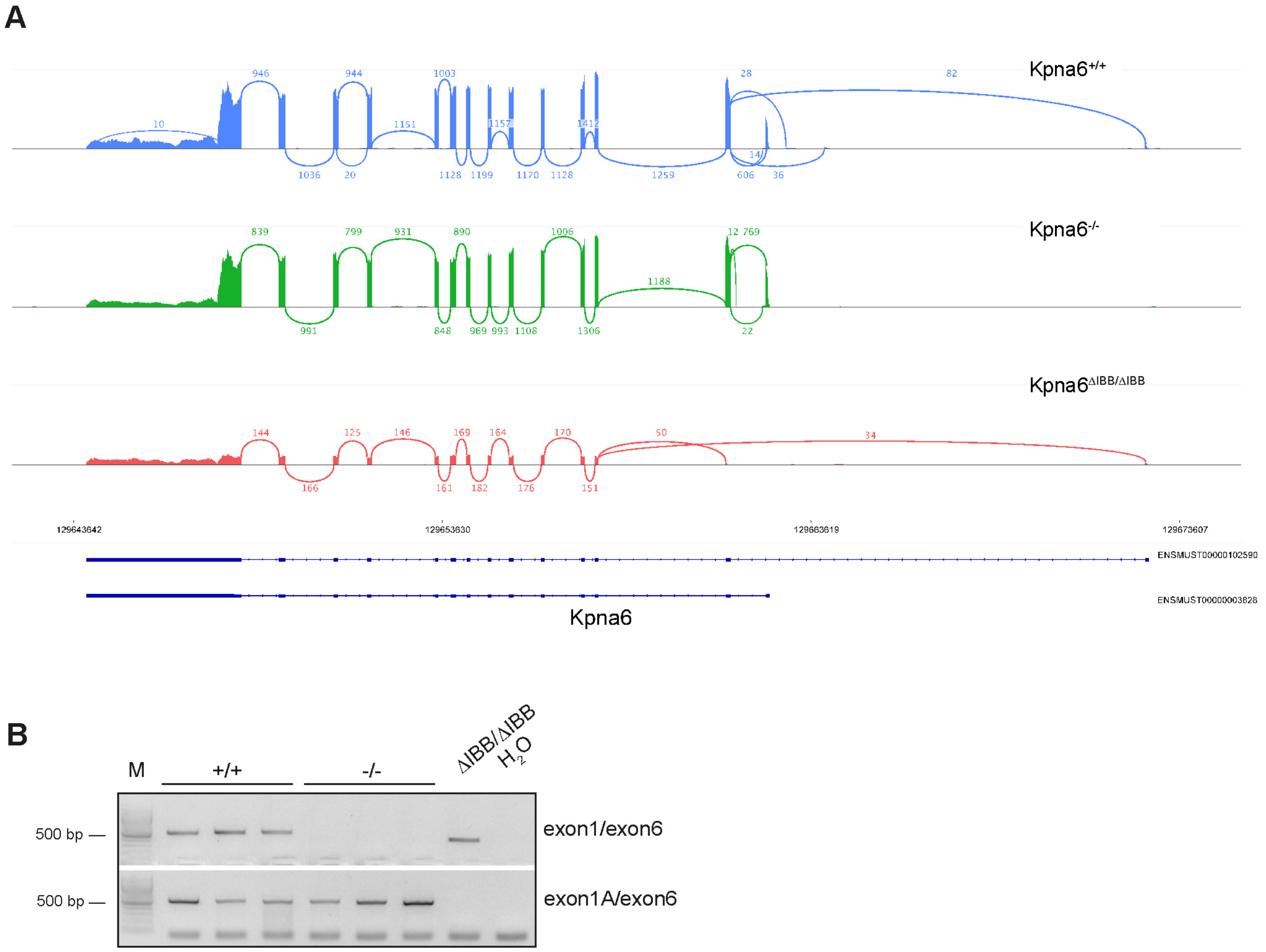

Fig. S1. (A) The Sashmi plot depicts a histogram of the number of reads covering the exons on Chr4 of the murine *Kpna6* genes in WT (top) and under the two knockout conditions (*Kpna6*$^{-/-}$ and *Kpna6*$^{\Delta IBB/\Delta IBB}$, middle and bottom). The numbers above or below the connecting arcs denote the number of reads spanning across the respective exons. The two *Kpna6* transcripts are depicted below in dark blue showing the exon/intron structure. Black numbers above the transcripts mark the position on the chromosome. Note how in the *Kpna6*$^{-/-}$ condition the first exon is missing (corresponding to the longer transcript; Ensembl database ENSMUST00000102590), while the shorter transcript (ENSMUST00000003828; starting from exon 1A) is fully expressed. Contrary to this, *Kpna6*$^{\Delta IBB/\Delta IBB}$ shows an approximately five-fold reduced number of reads across all exons. Here, no expression from exon1A is detected (which is deleted), and a low number of reads is detected from exon2, which is partly deleted by conventional knockout technology. (B) RT-PCR of *Kpna6* transcripts in FACS-sorted round spermatids. In WT germ cells, both promoters are used, while in *Kpna6*$^{-/-}$ germ cells, only transcription from the intronic promoter can be found. Note, how in *Kpna6*$^{\Delta IBB/\Delta IBB}$ the transcript from exon 1A is shorter, as part of the exon2 is deleted. M: 100bp marker.

Development • Supplementary information

Fig. S2

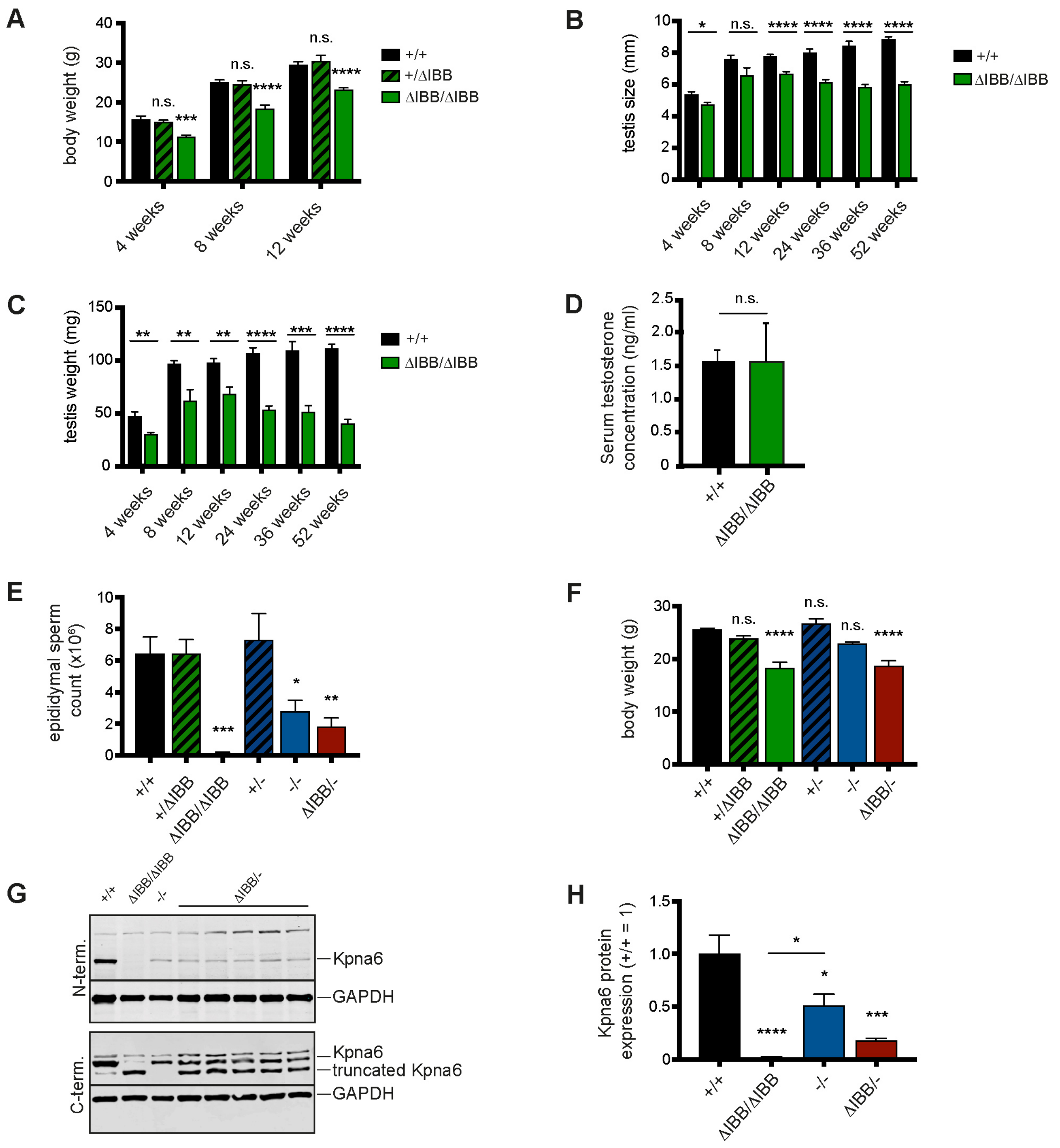

Development • Supplementary information

Fig. S2. (A) Body weight of WT (+/+), heterozygous *Kpna6*$^{+/\Delta IBB}$ and *Kpna6*$^{\Delta IBB/\Delta IBB}$ mice at various time points (n=5-18), showing that heterozygous mice do not display growth retardation. (B) Testis size at various time points (n=6-18). (C) Testis weight at various time points (n=6-18). (D) Serum testosterone concentration of WT (+/+) and *Kpna6*$^{\Delta IBB/\Delta IBB}$ mice. (E) Epididymal sperm count of all mutant lines (aged 9 weeks) including compound heterozygous mice (*Kpna6*$^{\Delta IBB/-}$) compared to WT (+/+) mice (n=4-6). (F) Body weight of all mutant mouse lines (aged 9 weeks). (G) Western blot analysis of testis protein extracts with anti-Kpna6 antibodies show, that *Kpna6*$^{\Delta IBB/-}$ testes express the full-length and the truncated protein. Part of this Western blot is shown in Fig. 1B. (H) Quantification of Western blots using the N-terminal antibody reveals a further reduction of Kpna6 in *Kpna6*$^{\Delta IBB/-}$ testes (n=4-5).

Fig. S3

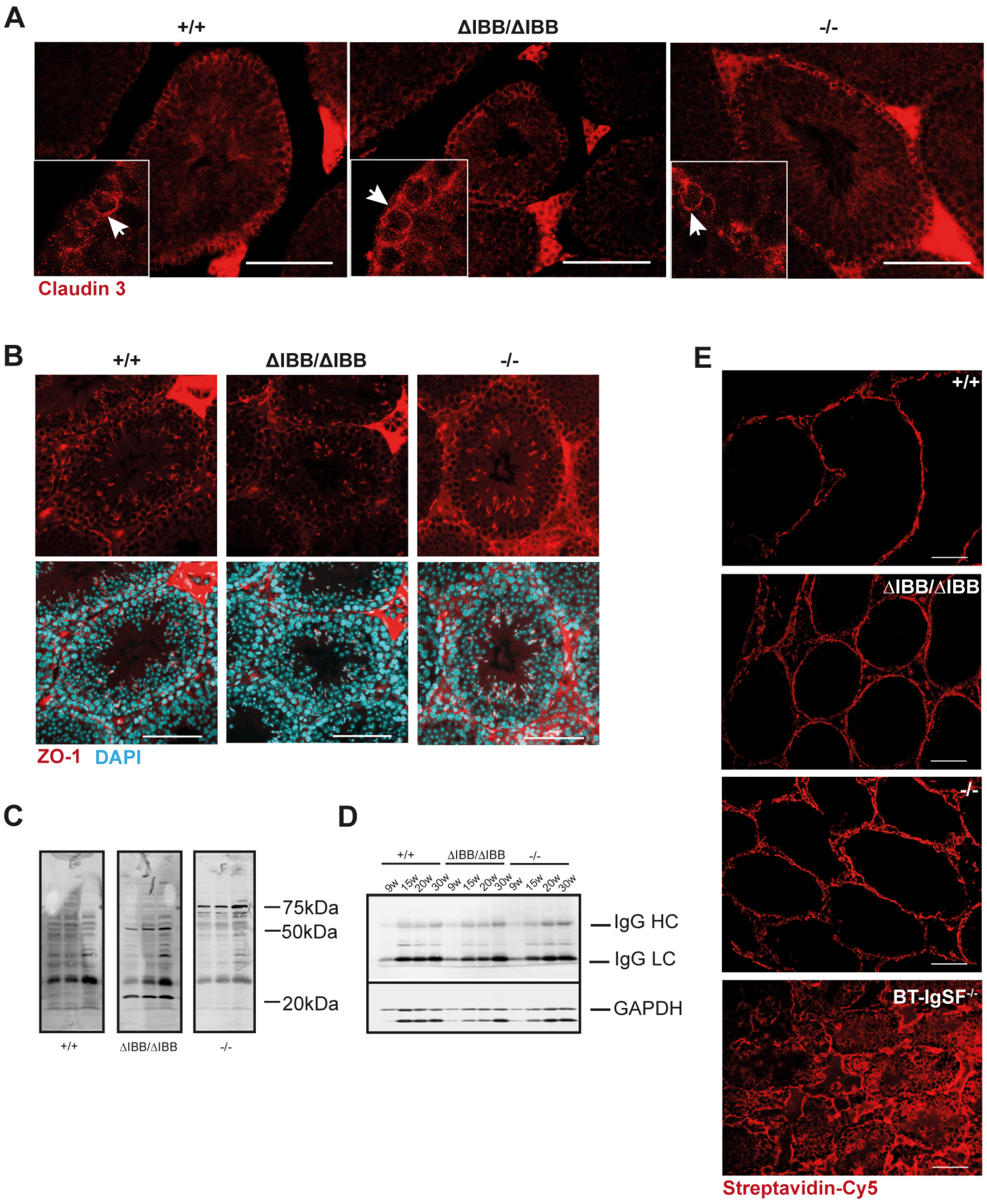

Development • Supplementary information

Fig. S3. (A) Cldn3 (red) staining of adult WT, *Kpna6*$^{\Delta IBB/\Delta IBB}$ and *Kpna6*$^{-/-}$ testes. Arrows mark Cldn3 staining of stage VIII seminiferous tubules. Scale bars: 100 µm. (B) ZO-1 (red) and DAPI (blue) staining of testis sections of WT, *Kpna6*$^{\Delta IBB/\Delta IBB}$ and *Kpna6*$^{-/-}$ mice. Scale bars: 100 µm. (C) Western blot analysis of WT testis protein extracts probed with sera from WT, *Kpna6*$^{\Delta IBB/\Delta IBB}$ and *Kpna6*$^{-/-}$ mice at 16 weeks of age. Lines mark 20-, 50- and 75-kDa testicular antigens recognized by antibodies (autoantibodies) present in serum samples of different *Kpna6*$^{\Delta IBB/\Delta IBB}$ and *Kpna6*$^{-/-}$ but not WT males. (D) Representative Western blot of immunoglobulins in testis extracts of WT, *Kpna6*$^{\Delta IBB/\Delta IBB}$ and *Kpna6*$^{-/-}$ mice at different ages. (E) Biotin diffusion assay marking the integrity of the BTB. Biotin is visualized by streptavidin-Cy5 staining (red). The *Kpna6*$^{\Delta IBB/\Delta IBB}$ and *Kpna6*$^{-/-}$ tubules show no major changes of biotin distribution compared to WT seminiferous tubules. As a control, a BT-IgSF knockout mouse was analyzed, which has recently been published to show a severe disruption of the BTB (Pelz et al., 2017). Scale bars: 100 µm.

Development • Supplementary information

Fig. S4

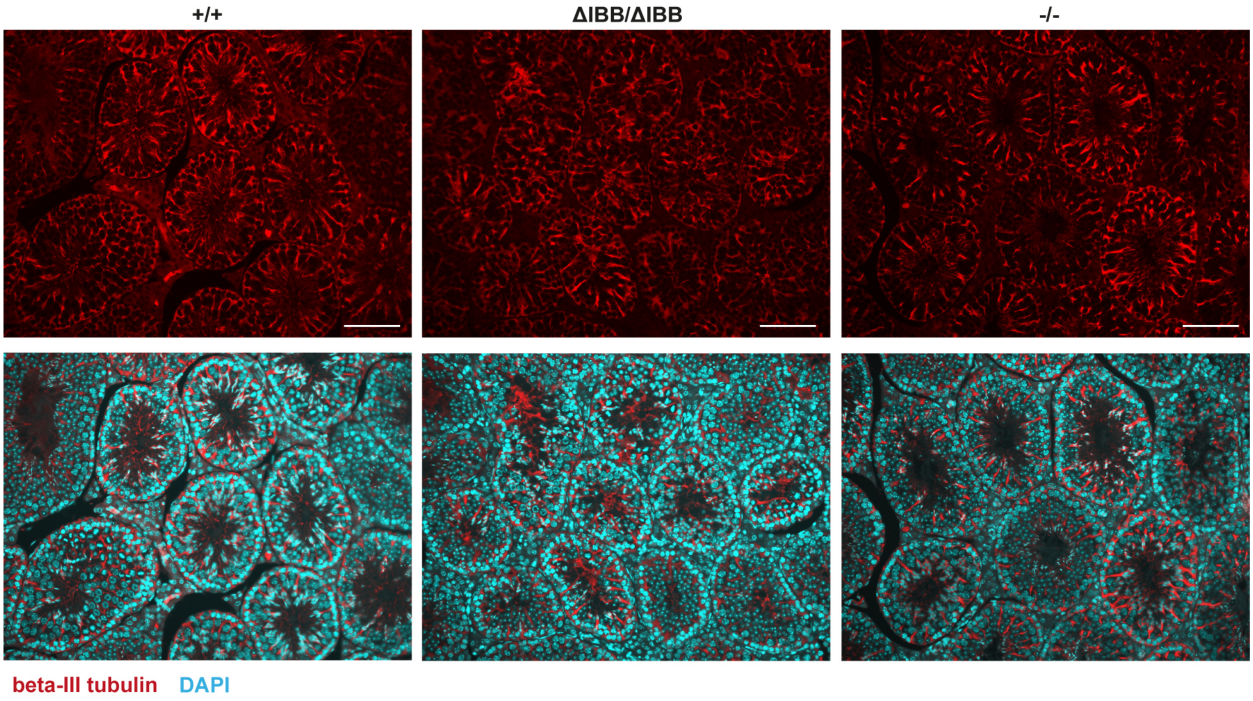

Fig. S4. Beta-III tubulin (red) and DAPI (blue) staining of adult WT, *Kpna6*$^{\Delta IBB/\Delta IBB}$ and *Kpna6*$^{-/-}$ testes. No differences can be found in the expression and distribution of beta-III tubulin in the seminiferous tubules of all three lines. Scale bars: 100 µm.

Fig. S5

A

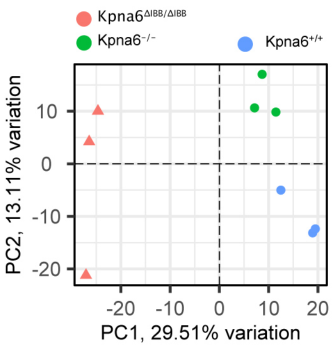

B

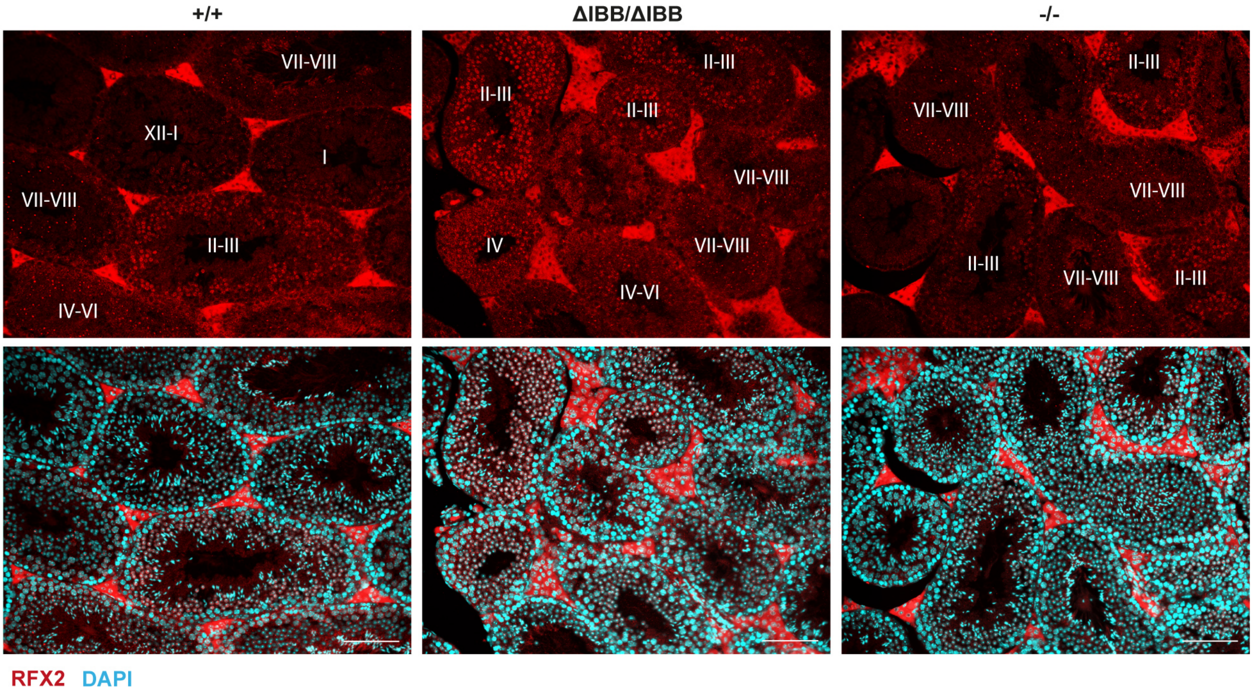

Fig. S5. (A) Principal component analysis of the gene-aggregated expression values as measured in transcripts per million of the 50% most variable genes across all samples. (B) Immunofluorescence for Rfx2 in WT, *Kpna6*$^{\Delta IBB/\Delta IBB}$ and *Kpna6*$^{-/-}$ testis on paraffin sections. Rfx2 is strongly expressed in nuclei of step 2-3 round spermatids. In step 4-8 round spermatids the protein localizes to a distinct spot in the nucleus, and when elongation of spermatids starts, the protein cannot be detected anymore. No abnormal staining pattern could be observed in *Kpna6*$^{\Delta IBB/\Delta IBB}$ and *Kpna6*$^{-/-}$ testes. Roman numbers mark the tubular stages. Scale bars: 100 µm.

Development • Supplementary information

Fig. S6

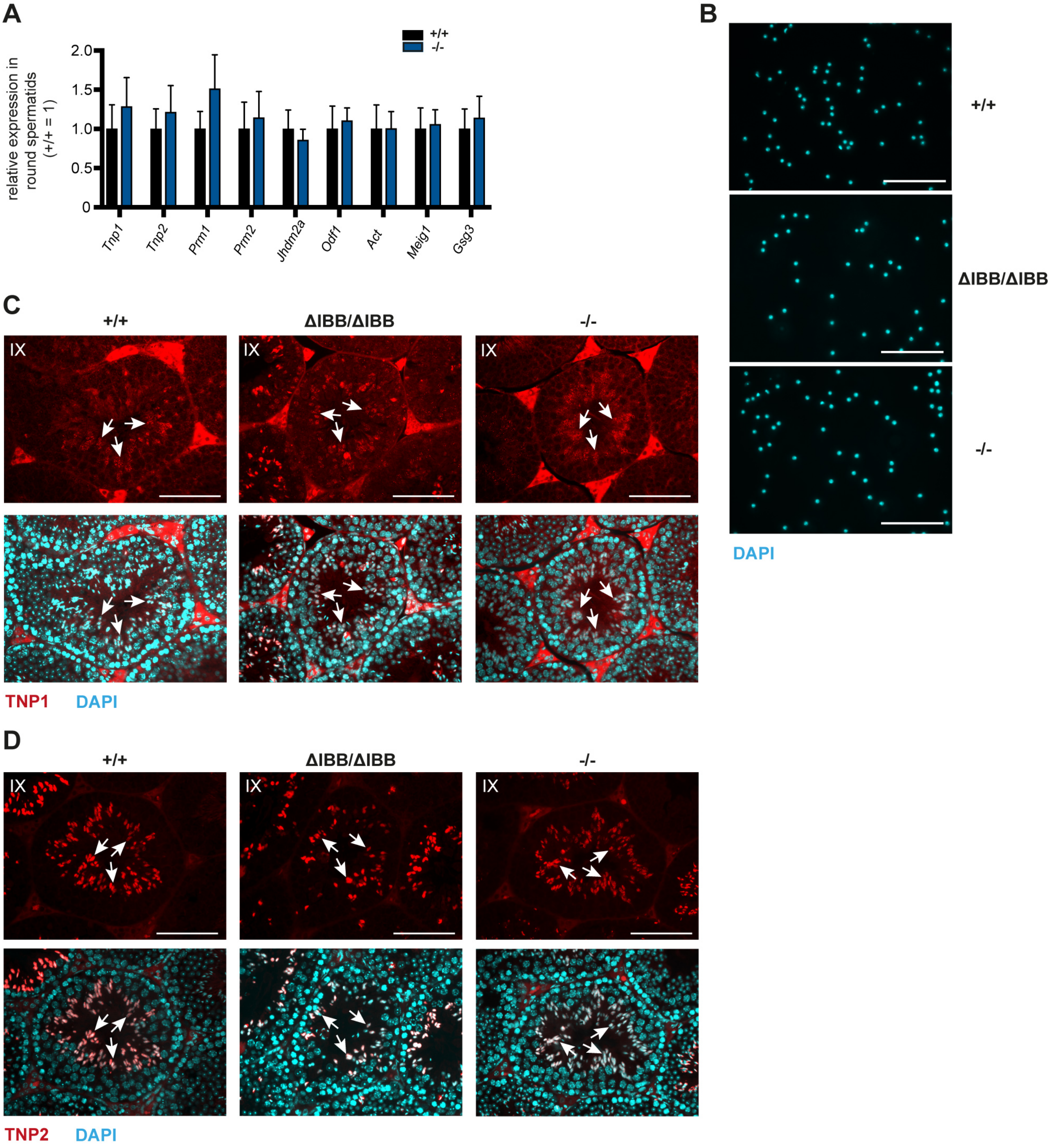
A
relative expression in round spermatids (+/+ = 1)
2.0
1.5
1.0
0.5
0
+/+
-/-
Tnp1
Tnp2
Prm1
Prm2
Jhdm2a
Odf1
Act
Meig1
Gsg3
B
+/+
ΔIBB/ΔIBB
-/-
DAPI
C
+/+
ΔIBB/ΔIBB
-/-
IX
TNP1
DAPI
D
+/+
ΔIBB/ΔIBB
-/-
IX
TNP2
DAPI

Fig. S6. (A) Quantitative realtime PCR analysis of various postmeiotic genes in isolated round spermatids of adult (12-20 weeks of age) WT and *Kpna6*$^{-/-}$ testes (n=4-6). (B) Following FACS-sorting, every sample was assessed with regard to purity of sorted round spermatids (average ± SD: WT 95.4% ± 0.02, α7$^{\Delta IBB/\Delta IBB}$ 93.2% ± 0.03, α7$^{-/-}$ 90.9% ± 0.03, no cells other than round spermatids or elongating sperms were found in the FACS-sorted samples). (C, D) TNP1 (red, C) and TNP2 (red, D) and DAPI (blue) staining of stage IX seminiferous tubules of WT, *Kpna6*$^{\Delta IBB/\Delta IBB}$ and *Kpna6*$^{-/-}$ testes showing a regular expression and localization of both proteins in early elongating spermatids (arrows). Scale bars: 100 µm.

Development • Supplementary information

Fig. S7

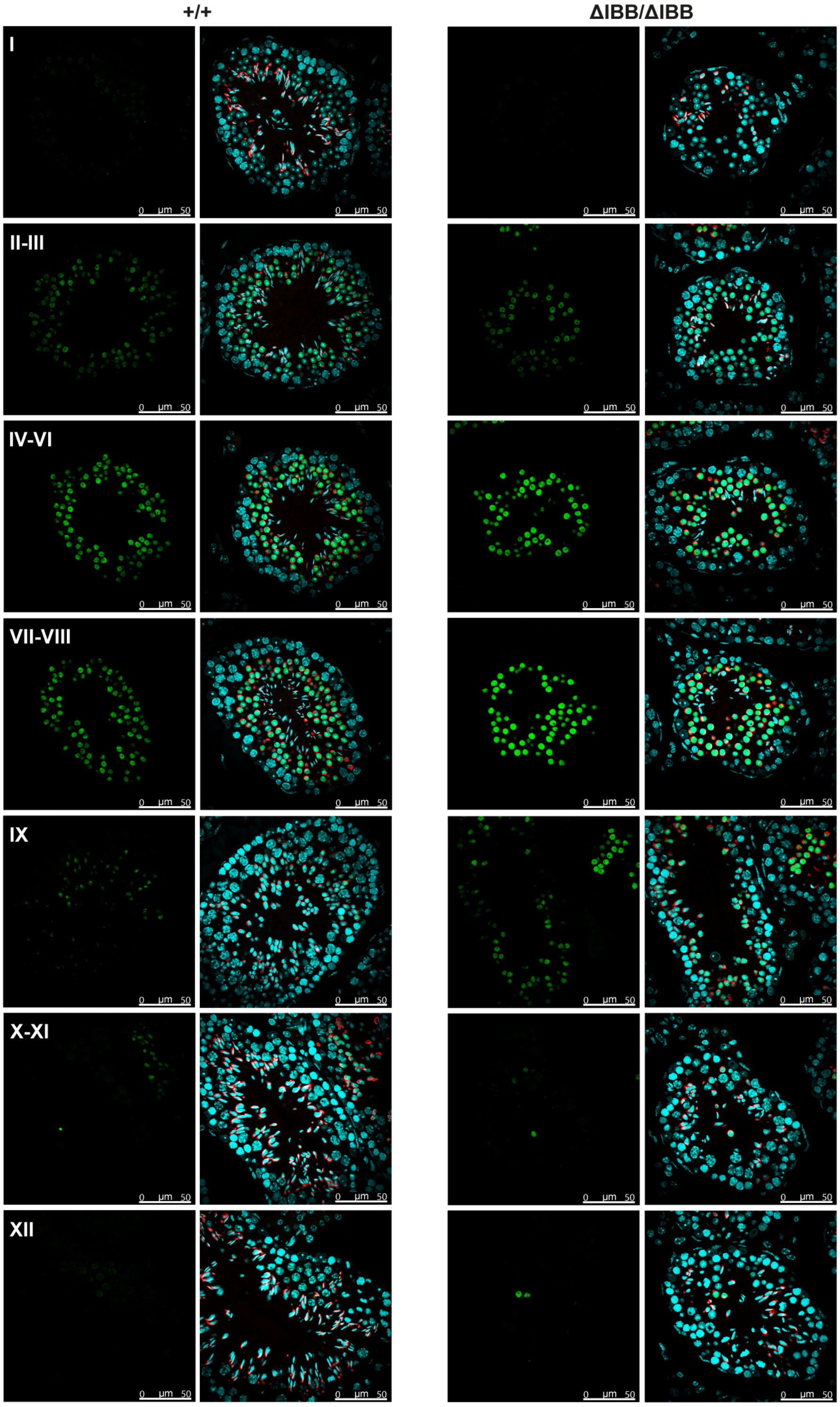

Fig. S7. Immunofluorescence for Crem (green) in WT and α7$^{\Delta IBB/\Delta IBB}$ testis, counterstained with DAPI (blue) and PNA (red, merge right panel). Roman numbers mark the tubular stages. Scale bars: 50 µm.

Development • Supplementary information

Fig. S8

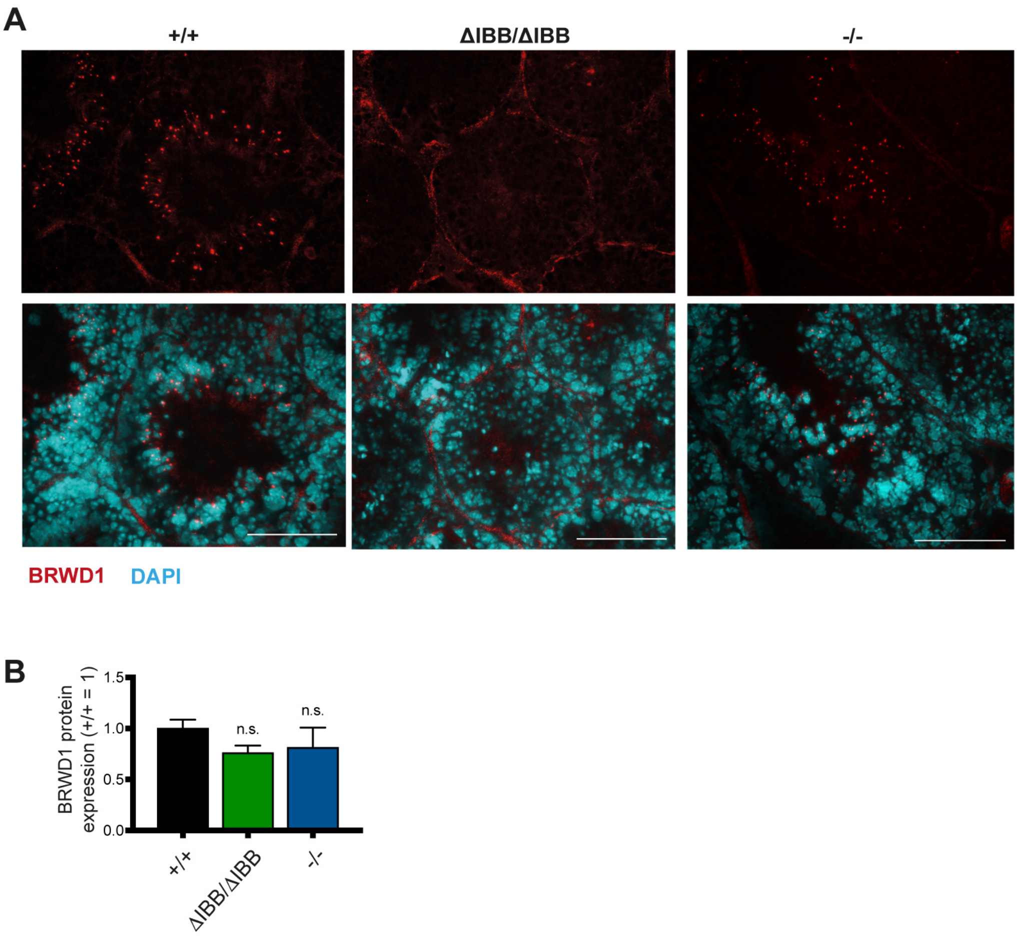

Fig. S8. (A) Immunofluorescence for BRWD1 (red) in WT, *Kpna6*$^{\Delta IBB/\Delta IBB}$ and *Kpna6*$^{-/-}$ testis on snap-frozen cryosections; counterstained with DAPI (blue), scale bars: 100 µm. (B) Quantification of BRWD1 Western blot signals normalized to ß-tubulin (α7$^{+/+}$: n=4, α7$^{\Delta IBB/\Delta IBB}$: n=4, α7$^{-/-}$: n=6). Age of mice: 12-16 weeks.

Development • Supplementary information

Table S1. List of genes, which are differentially expressed in *Kpna6*$^{\Delta IBB/\Delta IBB}$ (named *Kpna6*$^{\Delta IBB}$ in the list) and *Kpna6*$^{-/-}$ (named *Kpna6*$^{-}$ in the list) testes compared to WT and to each other. Significantly regulated genes (p-value < 0.01; absolute effect size > 1) are highlighted.

Click here to download Table S1

Table S2. List of genes, which are differentially expressed in testis of Rfx2 KO or in *Kpna6*$^{\Delta IBB/\Delta IBB}$ (named *Kpna6*$^{\Delta IBB}$ in the list) compared to their respective WT controls (P-value cutoff < 0.01, absolute effect size > 0.5).

Click here to download Table S2

Table S3. GSEA of *Kpna6*$^{\Delta IBB/\Delta IBB}$ (named *Kpna6*$^{\Delta IBB}$ in the list) and Rfx2 knockout testes.

Click here to download Table S3

Development • Supplementary information

Table S4. List of primary and secondary antibodies

Name	Company	Conditions
Kpna6 (C-terminal)	selfmade (peptide QPEAPMEGFQL) (Kohler et al. 1999)	1:10,000 (Western blot) 1:1,000 (Immunofluorescence)
Kpna6 (N-terminal)	selfmade (peptide MASPGKDNYR) (Kohler et al. 1999)	1:2,000 (Western blot) 1:200 (Immunofluorescence)
WT1	Abcam (#15249)	1:100 (Immunofluorescence)
Androgen receptor	Santa Cruz Biotechnology (10310, #sc816G)	1: 1000 (Western blot) 1:100 (Immunofluorescence)
Claudin 3	Acris Antibodies (# AP15488PU)	1:100 (Immunofluorescence)
ZO-1	Invitrogen (#339100)	1:100 (Immunofluorescence)
Vimentin	Cell signalling (#5741)	1:100 (Immunofluorescence)
ß3 Tubulin	Abcam (#ab52901)	1:100 (Immunofluorescence)
acetyl-Histone H4 (Lys8)	Upstate (#06-760)	1:200 (Immunofluorescence)
SALL4	Santa Cruz Biotechnology (EE-30, #sc101147)	1:100 (Immunofluorescence)
anti-BrdU	Biozol (#OBT0030)	1:500 (Immunofluorescence)
γH2Ax (Ser 139)	Cell Signaling (#9718)	1:200 (Immunofluorescence)
TNP1	Invitrogen (#PA5-44078)	1:200 (Immunofluorescence)
TNP2	Santa Cruz Biotechnology (K18, #sc21106)	1:1,000 (Western blot) 1:100 (Immunofluorescence)
ß-Actin	Cell Signaling (#4967)	1:1,000 (Western blot)
GAPDH	Cell Signaling (#2118)	1:1,000 (Western blot)
dimethyl-Histone H3 (Lys9)	Upstate (#07-441)	1:100 (Immunofluorescence)
trimethyl-Histone H3 (Lys9)	Millipore (#05-1242)	1:100 (Immunofluorescence)
acetyl-Histone H3 (Lys9)	Sigma-Aldrich (#06-942)	1:100 (Immunofluorescence)
acetyl-Histone H3 (Lys14)	Millipore (#06-911)	1:100 (Immunofluorescence)
acetyl-Histone H4 (Lys12)	Upstate (#07-595)	1:100 (Immunofluorescence)
RFX2	Novus (#NBP2-13224)	1:100 (Immunofluorescence)
CREM	Novus (#NBP1-81760)	1:500 (Immunofluorescence)
BRWD1	Biorbyt (#orb255836)	1:100 (Immunofluorescence)
Lectin-PNA Alexa 488	life technologies (#L21409)	1:500 (Immunofluorescence)
Lectin-PNA Alexa 594	life technologies (#L32459)	1:500 (Immunofluorescence)
IRDye 800 donkey anti-mouse	LiCor (#926-32212)	1:10,000 (Western blot)
IRDye 800 donkey anti-rabbit	LiCor (#926-32213)	1:10,000 (Western blot)
streptavidin-Cy5	Thermo Fisher Scientific (#434316)	1:600 (Immunofluorescence)
goat anti-mouse Cy3	Abcam (#ab97035)	1:500 (Immunofluorescence)
goat anti-mouse Alexa 488	Invitrogen (#A110019)	1:500 (Immunofluorescence)
donkey anti-rabbit Cy3	Jackson ImmunoResearch (#711-165-152)	1:500 (Immunofluorescence)
donkey anti-rabbit Alexa 488	Invitrogen (#A21206)	1:500 (Immunofluorescence)
donkey anti-rat Cy3	Jackson ImmunoResearch (#711-165-153)	1:500 (Immunofluorescence)

Development • Supplementary information

Table S5. List of primers of target genes for PCR and real-time PCR

gene	Forward sequence (5'->3')	Reverse sequence (5'->3')
Kpna6	Ex1: AGG CTA CCG CTG AAG CTA CC<br>Ex1A: GGG ACA GCA CAG GCT CAA TC<br>Ex2: GCC TTA AAC CCT GAG GAA ATG	Ex6: GAC GTT CCA GAG GCA ATG TT
Rhox5	CAAGGAAGACTCGGAAGAACAG	CATAGGACCAGGAGCACCAG
Pem	CAAAATCTCGGTGTCGCAAA	GCAACACCAGTCCCTGAACA
Wt1	CCG CAA CCA AGG ATA CAG CAC	GGG GTC CTC GTG TTT GAA GG
Clusterin	GGTCCGCAGCCTCATGTC	CATCTCAAAGAAAGGCTGGAACA
Gata1	CAT CAG CAC TGG CCT ACT AC	GTA GAG TGC CGT CTT GCC ATA G
Cldn11	CGT CAT GGC CAC TGG TCT CT	GGC TCT ACA AGC CTG CAC GTA
Cldn3	GCGCCTTGCTGTGTTGCT	AGAGGATCTTGGTGGGTGCAT
Tnp1	GAGAGGTGGAAGCAAGAGAAAA	CCCACTCTGATAGGATCTTTGG
Tnp2	CTGCCCCAAGAACAGGAAGA	CCGTTTCCGCCTCCTGA
Prm1	AGGTGTAAAAAATACTAGATGCACAGAAT AG	TTCAAGATGTGGCGAGATGCT
Prm2	GAATAGTCACCTGCCCAAGCA	GCAGCTCAGGGCTCAGACA
Jhdm2a	TGAAGGAAAAGAGAAGCCAGG	CTGATCGTGGATAGGGTCATG
Odf1	CCATCGCTCCGCAGTTTAG	AGACCTTCCCATCTTTCACG
Act	ACAGACTGCTATTCCAACGAG	TTGGTTCCTATTGGCTGTCG
Meig1	AACAAGCAGGATATCGGGATG	AAAGTATTGTCCCTCCGCTG
Gsg3	TGAGTAACACCTTGAATGGGC	CTCTTTCCTACTCAAGACGCTG
Gapdh	CTTTGTCAAGCTCATTTCCTGG	TCTTGCTCAGTGTCCTTGC
