# Supplementary material for: Kpna6 deficiency causes infertility in male mice by disrupting spermatogenesis
Source: Development. 2021 Oct 4;148(19):dev198374. doi: 10.1242/dev.198374 (PMC8513612; doi:10.1242/dev.198374)
Supplement: Reviewer comments [file develop-148-198374_review_history.pdf]

Kpna6 deficiency causes infertility in male mice by disrupting spermatogenesis

Na Liu, Fatimunnisa Qadri, Hauke Busch, Stefanie Huegel, Gabin Sihn, Ilya Chuykin, Enno Hartmann, Michael Bader and Franziska Rother
DOI: 10.1242/dev.198374

Editor: Patrick Tam

Review timeline

Original submission:	17 November 2020
Editorial decision:	6 January 2021
First revision received:	11 June 2021
Editorial decision:	2 July 2021
Second revision received:	16 August 2021
Accepted:	18 August 2021

Original submission

First decision letter

MS ID#: DEVELOP/2020/198374

MS TITLE: Importin α7 deficiency causes infertility in male mice by disrupting spermatogenesis

AUTHORS: Na Liu, Fatimunnisa Qadri, Hauke Busch, Stefanie Huegel, Gabin Sihn, Ilya Chuykin, Enno Hartmann, Michael Bader, and Franziska Rother

I have now received all the referees' reports on the above manuscript, and have reached a decision. The referees' comments are appended below, or you can access them online: please go to BenchPress and click on the 'Manuscripts with Decisions' queue in the Author Area.

As you will see, the referees express considerable interest in your work, but have some significant criticisms and recommend a substantial revision of your manuscript before we can consider publication. If you are able to revise the manuscript along the lines suggested, which may involve further experiments, I will be happy receive a revised version of the manuscript. Your revised paper will be re-reviewed by one or more of the original referees, and acceptance of your manuscript will depend on your addressing satisfactorily the reviewers' major concerns. Please also note that Development will normally permit only one round of major revision.

We are aware that you may be experiencing disruption to the normal running of your lab that make experimental revisions challenging. If it would be helpful, we encourage you to contact us to discuss your revision in greater detail. Please send us a point-by-point response indicating where you are able to address concerns raised (either experimentally or by changes to the text) and where you will not be able to do so within the normal timeframe of a revision. We will then provide further guidance. Please also note that we are happy to extend revision timeframes as necessary.

Please attend to all of the reviewers' comments and ensure that you clearly highlight all changes made in the revised manuscript. Please avoid using 'Tracked changes' in Word files as these are lost in PDF conversion. I should be grateful if you would also provide a point-by-point response detailing how you have dealt with the points raised by the reviewers in the 'Response to Reviewers' box. If you do not agree with any of their criticisms or suggestions please explain clearly why this is so.

© 2021. Published by The Company of Biologists under the terms of the Creative Commons Attribution License (https://creativecommons.org/licenses/by/4.0/).

Editor's note:
Given that importin alpha7/KPNA6 is likely to mediate nucleocytoplasmic trafficking, an appropriate mechanistic understanding of the elemental attribute, such as the identity and function of the interacting partners – cargo proteins – of KPNA6 and its testis-specific variant would enhance the merit of this study.

Reviewer 1

Advance summary and potential significance to field

Liu et al. reported loss of function analyses of Importin arpha7 and found that this protein is required for normal sperm formation. The authors generated two kinds of mutant mouse lines; one produces a truncated non-functional form of arpha7 and the other produces full-length of arpha7 only in germ cells but not in Sertoli cells in testes. Using these two lines, the authors asked possible function and the mechanism leading to the abnormal spermatogenesis due to the lack of arpha7 in germ cells and/or Sertoli cells. Many analyses were conducted and data is well presented based on those results. I evaluate information provided by the authors that is valuable for researchers in the Developmental Biology field. However, there are many redundant descriptions and some results are not clearly concluded. I hope that the authors should improve the way of description and make more concise manuscripts.

Comments for the author

Specific comments:
1. The most frustrating part in reading this manuscript was that I could not get a clear answer for the phenotypes due to the defect in Sertoli cells. The expression of arpha7 in Sertoli cells is lost in both mutants. Therefore, a common defect would be caused by the defect in Sertoli cells. I expected to see an earlier defect since arpha7 expression is missing up to meiotic spermatocyte and the expression starts after round spermatid stages. However, the authors showed earlier phenotype only using dIBB mutant but not arpha7(-/-) in Fig. 8. I could not understand why the authors did not examine or describe phenotype in arpha7(-/-) that also lack expression in Sertoli cells.

2. The expression pattern of other arpha importins should be described in the introduction part and give us the answer why the authors say that importin arpha genes have distinct expression patterns in mouse testis. Is the reason why the major defect is observed mainly only in spermatogenesis that the importin arpha7 is the major or only importin arpha expressed in testes?

3. The authors found many defects and phenotypes both in germ cells and Sertoli cells but those causal relationships are not well connected. Gene expression analyses led them to conclude that histone-protamine exchange is defective in the absence of arpha7. Unfortunately, the expression pattern of Crem1, a possible upstream transcription factor, was not altered. The authors showed that Brwd1, a transcriptional regulator was downregulated in the mutant without showing protein expression pattern and discussion regarding the possible involvement to the phenotype. I understand that it would be difficult to find the real target of importin arpha7, but it is not appropriate to show a list of results and let the readers judge the significance.

4. The authors showed that the number of Sertoli cells are decreased due to the lack of arpha7. Is the reduction observed only in the adult stage? The age information is missing in many figures. I finally found in the method section telling that if not stated the male mice aged 10-20 weeks were used for the analyses. It is better to indicate the age of mice used for each figure legend.
In case of Sertoli cell number, I like to know when this reduction is induced.
Is this caused by detachment of Sertoli cells as shown? So, is the number gradually reduced with age?

5. The phenotype of vimentin is very clear and interesting. I think that this phenotype can be connected with sperm organization. The author also tried to explain but they gave up because some phenotype was not observed in arpha7(-/-) mice and ended by describing that spermatid persistence is not caused by Sertoli cell defects only. I think that it is true, but they can emphasize

© 2021. Published by The Company of Biologists under the terms of the Creative Commons Attribution License (https://creativecommons.org/licenses/by/4.0/).

the Sertoli phenotype more clearly since dBB mouse lacks arpha7 expression in germ cells, it is not surprising to find additional defects can be observed in dBB mouse.

6. The authors speculated that Rfx2 could be responsible for the defective spermiogenesis. However, based on the phenotype of Rfx2-KO, it is unlikely. The phenotype of Rfx2 is observed earlier than arpha7-KO and major genes affected in arpha7-KO such as Tnp, Prm genes are not downregulated in Rfx2-KO. If the antibody is available, they should check the protein localization change.

Reviewer 2

Advance summary and potential significance to field

Summary of Advance: This manuscript presents comprehensive and deep phenotyping of testis structure and function in mice lacking importin a7, a protein correctly referred to as KPNA6 by both MGI and HUGO (the human gene is KPNA6, italicized, and the mouse gene is Kpna6, italicized). Overall, the authors show that lack of KPNA6 in testes leads to multiple spermatogenesis defects and abnormalities of the Sertoli cells; excellent figures support these conclusions. Nonetheless, impact is limited because primary and downstream effects cannot be resolved.

Comments for the author

Commentary on Results and Suggestions: To investigate the role of KPNA6, the authors used two mutant mousselines, one with a truncated KPNA6 due to targeted deletion of exon 2 (Kpna6del) leading to testes without KPNA6, and one with a gene trap mutation that leads to absence of full-length KPNA6 (Kpna6-) in most tissues, but presence of the full-length protein in testes due to a testis-specific exon. As shown in Fig. 3, with an informative diagram in Fig. 3B KPNA6 is expressed in both spermatids and nuclei of Sertoli cells, although in the testis-expressing Kpna6- males, the protein is expressed normally in the germ cells but not in the Sertoli cells. Together these results predict pleiotropic germ-cell and testis effects of absence of the KPNA6 protein.
Overall, the Kpna6- mice express a mild Sertoli defect and the Kpna6del males express mixed germ-cell and Sertoli-cell defects, but keeping track of the genotypes, these differences and integrating them in analyses of double mutants is a challenge for the reader that would be helped with more emphasis on the diagrams of Fig. 1A.

Spermatogenesis was dramatically impaired in mice lacking KPNA6, as shown by the nice histology in Fig. 2; this convincingly demonstrates the direct or indirect importance of the protein for normal spermatogenesis and testis function. In spite of the fact that KPNA6 is not expressed in spermatogonia or early spermatocytes, there is delay in the onset of the first wave of spermatogenesis in the Kpna6del mutant males, but the precise cause or mechanism is not known, implicating complex pathways downstream of KPNA6 expression, likely in both germ cells and Sertoli cells. Indeed there is loss of Sertoli cells in both mutants, with deficiency in nuclear import of the androgen receptor (AR), crucial for normal Sertoli cell function. Here it would be useful for the authors to more fully compare the phenotype of KPNA6 deficiency (seemingly no specific arrest of meiosis) to that of SCARKO males lacking AR specifically in Sertoli cells, where there is abrupt arrest at the end of the first meiotic prophase. In an interesting further complication of the phenotype, the authors find no specific down-regulation of AR-responsive Sertoli cell-expressed genes nor is there severe impairment of the blood-testis barrier, which is more adversely affected in SCARKO males.

The detailed histological and cytological phenotype analyses were extended into molecular phenotyping. Analysis of overall transcriptome revealed the spermatid cytodifferentiation pathways (e.g., cilia, flagella and other sperm-related GO terms) were affected, as predicted by histological analyses. There are technical concerns about the molecular analyses. First, since the RNA-seq analyses were performed on whole testes, it is important to consider the extent to which genotype differences reflect differences in cellularity; what compensation was made for this? Second, although PCR analysis of spermatids (Fig. 11) is interesting, there is no information on the purity of the isolated cells, particularly from infertile mutants; this information is needed for full interpretation of the results.

© 2021. Published by The Company of Biologists under the terms of the Creative Commons Attribution License (https://creativecommons.org/licenses/by/4.0/).

The Discussion is overly long, covering many aspects of spermatogenesis and testis function, but not integrating them into a pathway. This lack reflects the major concern about this work, namely overall impact. There is generally no concern about what was done or how it was done (both excellent). However, in spite of dedicated effort, the considerable pleiotropism of gene effects has frustrated determination of the precise mechanisms and pathways involved. As the authors point out in the final sentence, much remains to be resolved to fully understand the role of KPNA6. For example, given that the mutant males suffer growth restriction, to what extent are the testis phenotypes due to this? Moreover, because effects of lack of KPNA6 are pervasive, affecting gene transcription in both germ cells and Sertoli cells as well as chromatin structure and proteins in germ cells, the routes and mechanisms to the final phenotypes must be diverse. Perhaps it is premature for a diagram of pathways from gene to multiple phenotypes, but the reader is left hungry for the kind of mechanistic understanding that might derive from knowledge of the most elemental step, namely identity of the interacting partners – cargo proteins – of KPNA6 and its testis-specific variant.

Minor Comment: In order to have these results incorporated into various curated databases, authors should use correct gene and protein nomenclature.

Reviewer 3

Advance summary and potential significance to field

Importin α family of proteins are involved in the nucleocytoplasmic transport, an essential process in cell physiology.
Several importin α are expressed in mammalian testis and differentially expressed as germ cells progress through mitotic, meiotic and haploid maturation. However, little is known about their function.
In their manuscript Liu and collaborators analyzed the testicular phenotype of importin α7 knockout taking advantage of two different animal models previously generated in their laboratory. In α7ΔIBB/ΔIBB mouse line the genomic modification of the Kpna6 gene induces the production of a truncated protein while in the α7−/− knockout mouse line, importin α7 is completely deleted in all tissues.
The homozygous females of both mice lines are infertile and this prompt the authors to further scrutinize the reproductive phenotype in males. In this manuscript the authors reported that male *a*7−/− male were found to be fertile while α7ΔIBB/ΔIBB were sterile. Interestingly, in *a*7−/− male even though the expression of the full-length importin α7 was abrogated in all tissues, in the testis it was partially restored due to an intronic alternative promoter driving the expression of the full-length importin *a*7 in germ cells.
The authors showed that importin *a*7 protein is expressed in the nuclei of round spermatids, elongating spermatids and Sertoli cells. Importantly importin α7 deficiency results in several defects during spermiogenesis including incomplete sperm maturation, germ cell loss and oligozoospermia in both mutant mouse lines.
The authors main claim is that importin α7 is essential for Sertoli cell function and regulation of spermatogenesis. This is the first manuscript that reveal an essential function for an importin α member in mammalian spermatogenesis, underscoring the importance of the nucleocytoplasmic transport during germ cell maturation.

Comments for the author

Overall, the manuscript is potentially interesting and suitable for publication in Development, but there are some points that requires additional clarification.

Major comments
1) While the data described are solid and of high quality, I have some concerns about the animal models employed in this study. In the α7ΔIBB/ΔIBB mouse line some of the observed defects could be due to dominant negative effect of the truncated protein. Additionally, in α7−/− mouse line the

© 2021. Published by The Company of Biologists under the terms of the Creative Commons Attribution License (https://creativecommons.org/licenses/by/4.0/).

expression level from the intronic promoter seems to generate a lower level of the full-length protein resulting in an incomplete rescue of function in germ cells. To further support data obtained, the impact of importin a7 KO in Sertoli or in germ cells can be addressed by germ cell transplantation and the analysis of donor-derived spermatogenesis.

To address the cell-autonomous functions of importin a7 in Sertoli cells germ cells from WT mice can be transplanted into busulfan-treated importin α7−/− recipient testis while the germ cell-autonomous functions can be addressed by transplanting germ cells from a7ΔIBB/ΔIBB mice into busulfan-treated recipient WT testis.

2) Sertoli cell population size is set during puberty and, importantly, the number of germ cells and Leydig cells that can be maintained in the adult testis depends on the size of the adult Sertoli cell population (PMID: 3125042, 28911170). The author suggested that in the adult mutant testis the reduction in Sertoli cell number is caused by their degeneration and shedding in the lumen of seminiferous tubules. However, given the defects found in mutant mice, the reduction in Sertoli cell is an important finding and should be further investigate (see below in comments to figure 5).
Additional comments (figure by figure)

Figure 1 The novel finding of the germ cell-specific alternative intronic promoter upstream of E1A should be better illustrate. Are the alternative 5'-end been sequenced and compared to BY353738.1 sequence? Data in C suggest that both Kpna6 promoters are active in WT testis (amplification of both exon1/exon6 and exon1A/exon6 amplicons). Is the promoter upstream of E1A driving importin α7 expression in germ cells also in WT testis? Are the two promoters (upstream of E1 and E1A) both active in WT germ cells? This could be easily assessed using total RNA of FACS-sorted germ cells from WT and importin α7-/- mice.

In C exon1/exon6 amplicons from liver and testis show different electrophoretic mobility. Are they different mRNA splicing variants?

Figure S1 In B, the amount of importin α7 full length in a7ΔIBB/- testis is very similar to α7 -/- testis. This is surprisingly considering that the levels of importin α7 expression in α7-/- testis should be higher (if not double) respect to α7ΔIBB/- testis.

Figure 2 C and D: is not clear why the diameter of the seminiferous tubules and epididymal sperm count are expressed as relative values to WT. The actual numerical values will be more informative for the readers.

Figure 5 A: how is Sertoli cell population size in mutant vs WT during early testis postnatal development? Is the Sertoli proliferation phase in immature testis affected by importin α7 loss?
C: AR is also expressed in peritubular cells. Is the nuclear AR expression also lost in peritubular cells of mutant mice?

Figure 6 The misalignment of sperm head α7ΔIBB/ΔIBB and α7-/- mice suggests a defect in the ectoplasmic specialization. How is the distribution of F-actin at stage VIII in the three mice lines?
Figure 7 There is no clear indication about germ cells were recognized and counted.
As for preleptotene spermatocytes, is the histogram showing the counts for BrdU-labelled S-phase preleptotene? What about BrdU-negative preleptotene?

Are the pachytene spermatocytes recognized and counted based solely on γH2AX staining?
At page 8, what is "the stage-specific appearance of γH2AX-positive chromatin"? are the authors referring to the γH2AX-staining pattern in the different germ cell types in a given stage? or are they indirectly referring to the absence of γH2AX-positive apoptotic cells? In mutant mice, is the reduction in leptotene/zygotene spermatocytes due to increased apoptosis?

Figure 8 In the mouse testis γH2AX staining decorates all spermatogonial type not only B spermatogonia (Blanco- Rodriguez, 2009).

Figure 9 and related supplementary tables The transcriptome-wide analysis revealed significant downregulation of Kpna6 expression in α7ΔIBB/ΔIBB compared WT mice, as expected. However, the observation that in these experiments Kpna6 show no significant differential expression between

© 2021. Published by The Company of Biologists under the terms of the Creative Commons Attribution License (https://creativecommons.org/licenses/by/4.0/).

WT and α7 -/- is puzzling considering the data in Figure 1B, C showing a clear downregulation of Kpn6a at both mRNA and protein level.

Figure 10 The micrographs do not clearly show the findings described in the text. How the abnormal nuclear condensation was assessed? Is it referred to chromatin condensation, nuclear reshaping or both? I suggest to show less cells along with a clear annotation of the different spermatid steps being compared between different mice lines for each stage of the cycle.

Figure 11 The data presented are from α7ΔIBB/ΔIBB and WT mice lines. Why the α7 -/- mice line is not included in the analysis? How is the level of Tpn1 and Tpn2 and protamines in round spermatids isolated from α7 -/- testis? is their expression levels restored by importin α7 full-length? B: it is not clear what the histogram stands for. Does it show the results from western blot quantification? why the western blot is not shown? how the levels of chromatin-bound Tnp2 were normalized?

Figure 12 Brwd1 staining pattern in WT testis is not convincing. Published data suggest that Brdwd1 should be detected in spermatocyte and round spermatids (PMID: 25547156). Moreover, I assume, Brdw1 should have a nuclear localization. In the micrographs, it’s no clear if Brwd1 is expressed in spermatocytes and round spermatids and whether the dotted Brwd1 immunoreactivity is localized in the nuclei. What about Brdw1 expression in α7 -/- mice?

First revision

Author response to reviewers' comments

Dear Dr. Tam,

thank you for considering our manuscript for a major revision thereby giving us the chance to further improve the manuscript. We have worked on the text and experiments and added new information.

Editor’s note: Given that importin alpha7/KPNA6 is likely to mediate nucleocytoplasmic trafficking, an appropriate mechanistic understanding of the elemental attribute, such as the identity and function of the interacting partners - cargo proteins - of KPNA6 and its testis-specific variant would enhance the merit of this study.

We stated more clearly in the text, that the protein structure of the two KPNA6 transcripts is the same, there is no testis-specific protein variant. Out of a list of importin α7 binders previously published by us using Co-IP in transfected cultured cells and GST pull-down of mouse tissue (Hügel et al., 2014; DOI: 10.1074/mcp.M112.026856), we selected several candidates and analyzed their level and localization of expression in testis of our knockout mice by immunofluorescence. An interesting candidate is BRG1, wich has been shown to be essential for the completion of meiosis (Wang, et al., 2012, DOI: 10.1095/biolreprod.111.097097). Furthermore, BRG1 had shown strong binding to importin α7 in cultured cells and mouse ovary (Hügel et al., 2014; DOI: 10.1074/mcp.M112.026856). In the testis, BRG1 is strongly expressed in pachytene spermatocytes of importin $\alpha7^{+/+}$, $\alpha7^{dIBB/dIBB}$ and $\alpha7^{-/-}$ mice and no differences in Brg1 staining was observed between the groups.

© 2021. Published by The Company of Biologists under the terms of the Creative Commons Attribution License (https://creativecommons.org/licenses/by/4.0/).

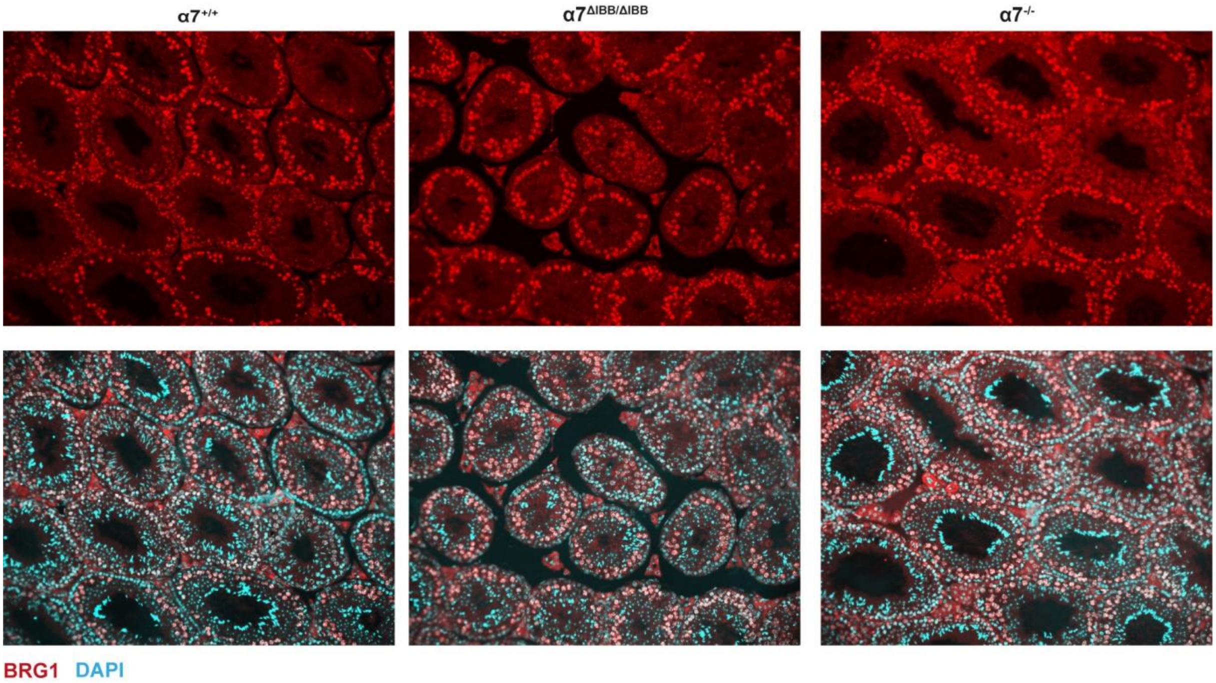

A second interesting candidate, MCM3, had shown strong binding of importin α7 in cultured cells and mouse ovary and additionally we have previously shown that nuclear import of MCM3 is disturbed in importin α7$^{-/-}$ cells (Hügel et al., 2014; DOI: 10.1074/mcp.M112.026856). However, analysis of MCM3 in the testis by immunofluorescence revealed a regular expression and localization in all three groups, excluding a major role in the infertility phenotype seen in importin α7$^{dIBB/dIBB}$ mice.

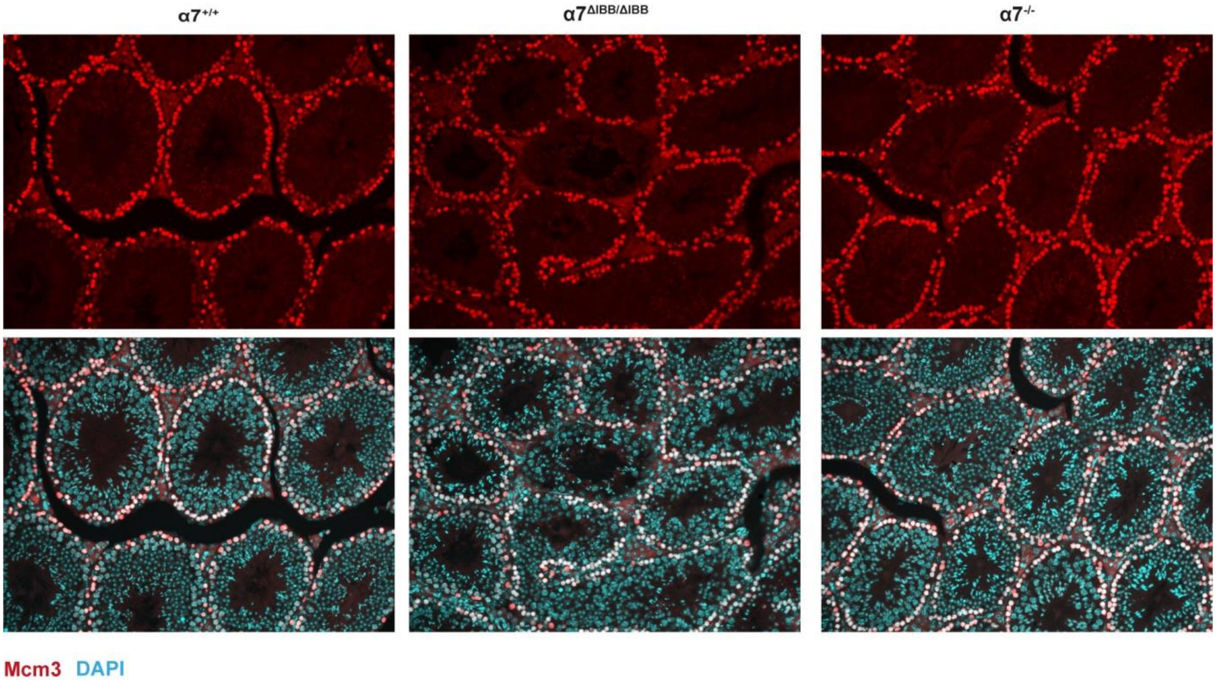

Reviewer 1

Liu et al. reported loss of function analyses of Importin arpha7 and found that this protein is required for normal sperm formation. The authors generated two kinds of mutant mouse lines; one produces a truncated non-functional form of arpha7 and the other produces full-length of arpha7 only in germ cells but not in Sertoli cells in testes. Using these two lines, the

© 2021. Published by The Company of Biologists under the terms of the Creative Commons Attribution License (https://creativecommons.org/licenses/by/4.0/).

authors asked possible function and the mechanism leading to the abnormal spermatogenesis due to the lack of arpha7 in germ cells and/or Sertoli cells. Many analyses were conducted and data is well presented based on those results. I evaluate information provided by the authors that is valuable for researchers in the Developmental Biology field. However, there are many redundant descriptions and some results are not clearly concluded. I hope that the authors should improve the way of description and make more concise manuscripts.

1. The most frustrating part in reading this manuscript was that I could not get a clear answer for the phenotypes due to the defect in Sertoli cells. The expression of arpha7 in Sertoli cells is lost in both mutants. Therefore, a common defect would be caused by the defect in Sertoli cells. I expected to see an earlier defect since arpha7 expression is missing up to meiotic spermatocyte and the expression starts after round spermatid stages. However, the authors showed earlier phenotype only using dIBB mutant but not arpha7(-/-) in Fig. 8. I could not understand why the authors did not examine or describe phenotype in arpha7(-/-) that also lack expression in Sertoli cells.

Analysis of γH2ax staining in 21d old importin $\alpha7^{-/-}$ mice were performed to complete the Fig. 8. However, for injection of BrdU a new animal license would have been needed that takes 3-4 months to get. So, we excluded this picture from the figures as it doesn't add new information.

2. The expression pattern of other arpha importins should be described in the introduction part and give us the answer why the authors say that importin arpha genes have distinct expression patterns in mouse testis. Is the reason why the major defect is observed mainly only in spermatogenesis that the importin arpha7 is the major or only importin arpha expressed in testes?

Changes in the text were performed.

3. The authors found many defects and phenotypes both in germ cells and Sertoli cells but those causal relationships are not well connected. Gene expression analyses led them to conclude that histone-protamine exchange is defective in the absence of arpha7. Unfortunately, the expression pattern of Crem1, a possible upstream transcription factor, was not altered. The authors showed that Brwd1, a transcriptional regulator was downregulated in the mutant without showing protein expression pattern and discussion regarding the possible involvement to the phenotype. I understand that it would be difficult to find the real target of importin arpha7, but it is not appropriate to show a list of results and let the readers judge the significance.

Analysis of BRWD1 protein expression by SDS page and Western blot was performed and added to the manuscript. The immunofluorescence staining for BRWD1 protein in paraffin embedded testis sections revealed a different staining pattern in importin $\alpha7^{dIBB/dIBB}$ testis compared with wildtype and $\alpha7^{-/-}$. This staining pattern was confirmed in cryosections. Quantitative determination of the BRWD1 protein levels by Western blot showed no differential regulation between the groups. We conclude that the staining pattern seen in both paraffin-and cryosectioned testis represents rather a differential localization than a difference in absolute protein level. To confirm our results, we ordered a third antibody against BRWD1. Unfortunately, any available antibodies are directed against the human protein and the newly bought antibody did neither function in histology of mouse testis nor in Western blots mouse tissue.
Changes in text have been performed.

4. The authors showed that the number of Sertoli cells are decreased due to the lack of arpha7. Is the reduction observed only in the adult stage? The age information is missing in many figures. I finally found in the method section telling that if not stated the male mice aged 10-20 weeks were used for the analyses. It is better to indicate the age of mice used for each figure legend. In case of Sertoli cell number, I like to know when this reduction is induced. Is this caused by detachment of Sertoli cells as shown? So, is the number gradually reduced with age?

The age information was included in the figures.

© 2021. Published by The Company of Biologists under the terms of the Creative Commons Attribution License (https://creativecommons.org/licenses/by/4.0/).

Young animals were analysed regarding Sertoli cell number. At the age of 4 weeks no difference in Sertoli cell numbers could be observed between the three groups (Fig. 5A), which leads to the conclusion that proliferation phase of the Sertoli cells is not affected. As the reduced Sertoli cell numbers are accompanied with occurrence of detached cells, we conclude, that the detachment of cells leads to the reduced Sertoli counts per tubule. Unfortunately, we did not have enough mice to analyse Sertoli cell numbers in old animals.

5. The phenotype of vimentin is very clear and interesting. I think that this phenotype can be connected with sperm organization. The author also tried to explain but they gave up because some phenotype was not observed in arpha7(-/-) mice and ended by describing that spermatid persistence is not caused by Sertoli cell defects only. I think that it is true, but they can emphasize the Sertoli phenotype more clearly since dBB mouse lacks arpha7 expression in germ cells, it is not surprising to find additional defects can be observed in dBB mouse.

Changes have been made in the text.

6. The authors speculated that Rfx2 could be responsible for the defective spermiogenesis. However, based on the phenotype of Rfx2-KO, it is unlikely. The phenotype of Rfx2 is observed earlier than arpha7-KO and major genes affected in arpha7-KO such as Tnp, Prm genes are not downregulated in Rfx2-KO. If the antibody is available, they should check the protein localization change.

An earlier phenotype of Rfx2 KO mice does not argue against a role in importin $\alpha7^{dlBB/dlBB}$ mice, as in Rfx2 KO its expression is substantially reduced while this is obviously not the case in importin $\alpha7^{dlBB/dlBB}$ mice (RNAseq data). Here, we proposed a defect in proper localization of Rfx2 protein due to a possible transport defect in the discussion. Accordingly, an Rfx2 antibody was ordered and immunohistochemistry was performed. We found, that Rfx2 is localized strongly in nuclei of step 2-3 round spermatids. From step 4 until step 8 of round spermatids the protein localizes to a distinct spot in the nucleus, and before elongation of spermatids start, it disappears (Fig. S4). We could not find a differing staining pattern in importin $\alpha7^{dlBB/dlBB}$ or $\alpha7^{-/-}$ testes. Nevertheless, the computational data show that 40% of the genes that were downregulated in importin $\alpha7^{dlBB/dlBB}$ testis, were also downregulated in Rfx2 KO, which we believe is not accidental. Although we currently do not understand how Rfx2 fits into the phenotype of importin $\alpha7^{dlBB/dlBB}$ mice, we will continue searching for co-factors, or analyze differential protein modifications such as phosphorylation to explain the role of Rfx2.

Concerning the argument of the reviewer, that "*major genes affected in arpha7-KO such as Tnp, Prm genes are not downregulated in Rfx2-KO*": Rfx2 is probably not the only affected upstream regulator, so a down-regulation of prms and tnps in importin $\alpha7^{dlBB/dlBB}$ mice might be attributed to other upstream molecules and does not argue against Rfx2 involvement in the phenotype.

Reviewer 2

This manuscript presents comprehensive and deep phenotyping of testis structure and function in mice lacking importin α7, a protein correctly referred to as KPNA6 by both MGI and HUGO (the human gene is KPNA6, italicized, and the mouse gene is Kpna6, italicized). Overall, the authors show that lack of KPNA6 in testes leads to multiple spermatogenesis defects and abnormalities of the Sertoli cells; excellent figures support these conclusions. Nonetheless, impact is limited because primary and downstream effects cannot be resolved.

To investigate the role of KPNA6, the authors used two mutant mousselines, one with a truncated KPNA6 due to targeted deletion of exon 2 (Kpna6del) leading to testes without KPNA6, and one with a gene trap mutation that leads to absence of full-length KPNA6 (Kpna6-) in most tissues, but presence of the full-length protein in testes due to a testis-specific exon. As shown in Fig. 3, with an informative diagram in Fig. 3B, KPNA6 is expressed in both spermatids and nuclei of Sertoli cells, although in the testis-expressing Kpna6- males, the protein is expressed normally in the germ cells but not in the Sertoli cells. Together these results predict pleiotropic germ-cell and testis effects of absence of the KPNA6 protein. Overall, the Kpna6- mice express a mild Sertoli defect and the Kpna6del males express mixed

© 2021. Published by The Company of Biologists under the terms of the Creative Commons Attribution License (https://creativecommons.org/licenses/by/4.0/).

germ-cell and Sertoli-cell defects, but keeping track of the genotypes, these differences and integrating them in analyses of double mutants is a challenge for the reader that would be helped with more emphasis on the diagrams of Fig. 1A.

References to Fig. 1A were made in the text where we think it is needed to guide the reader better through the complicated situation. The cell-type specific expression of importin α7 or its truncated form is mentioned in Fig. 1A (under "protein", in brackets).

Spermatogenesis was dramatically impaired in mice lacking KPNA6, as shown by the nice histology in Fig. 2; this convincingly demonstrates the direct or indirect importance of the protein for normal spermatogenesis and testis function. In spite of the fact that KPNA6 is not expressed in spermatogonia or early spermatocytes, there is delay in the onset of the first wave of spermatogenesis in the Kpna6del mutant males, but the precise cause or mechanism is not known, implicating complex pathways downstream of KPNA6 expression, likely in both germ cells and Sertoli cells. Indeed there is loss of Sertoli cells in both mutants, with deficiency in nuclear import of the androgen receptor (AR), crucial for normal Sertoli cell function. Here it would be useful for the authors to more fully compare the phenotype of KPNA6 deficiency (seemingly no specific arrest of meiosis) to that of SCARKO males lacking AR specifically in Sertoli cells, where there is abrupt arrest at the end of the first meiotic prophase. In an interesting further complication of the phenotype, the authors find no specific down-regulation of AR-responsive Sertoli cell-expressed genes nor is there severe impairment of the blood-testis barrier, which is more adversely affected in SCARKO males.

We compared the phenotypes of importin α7 mutants with SCARKO mice by analyzing Sertoli cell expressed genes and BTB but did not find big disturbances in alpha7 KO mice and this was included in the discussion. Discussion text was changed putting more emphasis on the phenotype of SCARKO mice and comparing them to KPNA6 KO, although both phenotypes show obviously big differences.

The detailed histological and cytological phenotype analyses were extended into molecular phenotyping. Analysis of overall transcriptome revealed the spermatid cytodifferentiation pathways (e.g., cilia, flagella and other sperm-related GO terms) were affected, as predicted by histological analyses. There are technical concerns about the molecular analyses. First, since the RNA-seq analyses were performed on whole testes, it is important to consider the extent to which genotype differences reflect differences in cellularity; what compensation was made for this? Second, although PCR analysis of spermatids (Fig. 11) is interesting, there is no information on the purity of the isolated cells, particularly from infertile mutants; this information is needed for full interpretation of the results.

We did not make compensations for the differences in cellularity, that is why we discuss the results of this part very cautiously and do not put too much weight on results found in RNAseq that might differ or contradict our other analyses (e.g. analyses of mRNAs in isolated round spermatids). A more critical discussion of the method is included in the discussions part. We performed an additional analysis with xCell (xcell.ucsf.edu) to rule out the presence of other (non testis specific) cell types, like immune cells in one of the groups. Here, we did not find any significant differences between the three groups.
Concerning the purity of isolated round spermatids, these cells were isolated by FACS sorting. Every population was checked by microscopy and Hoechst staining and purity of round spermatids was >90% (Fig. S5). This information is included in the methods part. The round spermatids were then used for quantitative analysis and normalized to *gapdh* to ensure that same amounts were analyzed.

The Discussion is overly long, covering many aspects of spermatogenesis and testis function, but not integrating them into a pathway. This lack reflects the major concern about this work, namely overall impact. There is generally no concern about what was done or how it was done (both excellent). However, in spite of dedicated effort, the considerable pleiotropism of gene effects has frustrated determination of the precise mechanisms and pathways involved. As the authors point out in the final sentence, much remains to be resolved to fully understand the role of KPNA6. For example, given that the mutant males suffer growth restriction, to what extent are the testis phenotypes due to this? Moreover, because effects of lack of KPNA6 are pervasive, affecting gene transcription in both germ

© 2021. Published by The Company of Biologists under the terms of the Creative Commons Attribution License (https://creativecommons.org/licenses/by/4.0/).

cells and Sertoli cells as well as chromatin structure and proteins in germ cells, the routes and mechanisms to the final phenotypes must be diverse. Perhaps it is premature for a diagram of pathways from gene to multiple phenotypes, but the reader is left hungry for the kind of mechanistic understanding that might derive from knowledge of the most elemental step, namely identity of the interacting partners - cargo proteins - of KPNA6 and its testis-specific variant. Minor Comment: In order to have these results incorporated into various curated databases, authors should use correct gene and protein nomenclature.

We have shortened the discussion part.

An additional analysis of body weight was performed for all groups of mice (including heterozygotes of both lines and compound mice having 2 different defective alleles). Comparing 9 weeks old animals, we found a reduced body weight in importin α7$^{dIBB/dIBB}$, as was seen before. Additionally we observed a reduced body weight in importin α7$^{dIBB/-}$, but not in α7$^{dIBB/+}$ mice, ruling out a dominant negative effect of the truncated protein. We conclude, that one fully functional importin α7 allele is enough to prevent the growth restriction. Interestingly, the α7$^{dIBB/-}$ show the same growth restriction as the α7$^{dIBB/dIBB}$ mice but are fertile. Moreover, the growth restriction in importin α7$^{-/-}$ mice at the age of 16 weeks is similar to α7$^{dIBB/dIBB}$ mice (Fig. 1D) while fertility is persistent. Thus growth restriction and infertility are not linked.
As the reviewer says correctly, we believe that the routes leading to the phenotype in mice lacking importin α7 are diverse and therefore it is very difficult at this stage of research to make mechanistic statements. Out of a list of importin α7 binders previously published by us using Co-IP in transfected cultured cells and GST pull-down of mouse tissue (Hügel et al., 2014; DOI: 10.1074/mcp.M112.026856), we selected several candidates and analyzed their level and localization of expression in testis of our knockout mice by immunofluorescence (see Answers to Editor above). Unfortunately, none of them did show differential expression in importin α7-deficient mice.
The testis-specific transcript leads to a normal protein that cannot be distinguished from the importin α7 protein found elsewhere. We made this clearer in the manuscript.
Since the beginning of research on importins/karyopherins there has been a discussion about the names. While database entries of the mouse gene refer to “Kpna6”, the corresponding protein nomenclature is “Importin subunit alpha-7” (IMA7_MOUSE, O35345 Uniprot). As we refer in most of our analyses to the protein, not the gene, we decided to use the term importin α7, especially as it has been used in previous publications of our group. We checked again carefully the manuscript to make sure that the nomenclature of the gene is correctly named “Kpna6”.

Reviewer 3

Importin a family of proteins are involved in the nucleocytoplasmic transport, an essential process in cell physiology. Several importin a are expressed in mammalian testis and differentially expressed as germ cells progress through mitotic, meiotic and haploid maturation. However, little is known about their function. In their manuscript Liu and collaborators analyzed the testicular phenotype of importin a7 knockout taking advantage of two different animal models, previously generated in their laboratory. In a7ΔIBB/ΔIBB mouse line the genomic modification of the Kpna6 gene induces the production of a truncated protein while in the a7−/− knockout mouse line, importin a7 is completely deleted in all tissues. The homozygous females of both mice lines are infertile and this prompt the authors to further scrutinize the reproductive phenotype in males. In this manuscript the authors reported that male a7−/− male were found to be fertile while a7ΔIBB/ΔIBB were sterile. Interestingly, in a7−/− male even though the expression of the full-length importin a7 was abrogated in all tissues, in the testis it was partially restored due to an intronic alternative promoter driving the expression of the full-length importin a7 in germ cells. The authors showed that importin a7 protein is expressed in the nuclei of round spermatids, elongating spermatids and Sertoli cells. Importantly, importin a7 deficiency results in several defects during spermiogenesis including incomplete sperm maturation, germ cell loss and oligozoospermia in both mutant mouse lines. The authors main claim is that importin a7 is essential for Sertoli cell function and regulation of spermatogenesis. This is the first manuscript that reveal an essential function for an importin a member in mammalian

© 2021. Published by The Company of Biologists under the terms of the Creative Commons Attribution License (https://creativecommons.org/licenses/by/4.0/).

spermatogenesis, underscoring the importance of the nucleocytoplasmic transport during germ cell maturation.

While the data described are solid and of high quality, I have some concerns about the animal models employed in this study. In the a7ΔIBB/ΔIBB mouse line some of the observed defects could be due to dominant negative effect of the truncated protein. Additionally, in a7−/− mouse line the expression level from the intronic promoter seems to generate a lower level of the full-length protein resulting in an incomplete rescue of function in germ cells. To further support data obtained, the impact of importin a7 KO in Sertoli or in germ cells can be addressed by germ cell transplantation and the analysis of donor-derived spermatogenesis. To address the cell-autonomous functions of importin a7 in Sertoli cells, germ cells from WT mice can be transplanted into busulfan-treated importin a7−/− recipient testis while the germ cell-autonomous functions can be addressed by transplanting germ cells from a7ΔIBB/ΔIBB mice into busulfan-treated recipient WT testis.

We can rule out a dominant negative effect on fertility, testis organisation, growth/body weight and sperm counts. Analysing heterozygous $\alpha7^{dIBB/+}$ mice, that also express the truncated protein, did not give any hints for dominant negative effects. Testis histology was completely normal in these animals.
Indeed, expression from the alternative promoter seems to be lower in importin $\alpha7^{-/-}$ than in WT testis in the (not quantitative) analysis (see Fig. 1C). However, our sequencing data of whole testis RNA did not show any expression differences. To address this concern further, we carried out a Sashmi plot which showed, that expression from the alternative promoter leads to comparable amounts of *Kpna6* transcripts in WT and importin $\alpha7^{-/-}$ testes (Fig. S2A). On protein levels we find a reduced expression of importin α7 protein in whole testis of importin $\alpha7^{-/-}$ mice (Fig 1B and S1C) representing a cumulative effect of rescued expression in germ cells (from alternative promoter) and not rescued (and therefore missing) expression in all other cells. Western blot signals of importin α7 have been quantified and quantification was included into Fig. S1.
The suggested cell transplantation experiments would make a lot of sense but cannot be performed. We do neither have the expertise for germ cell transplantation in mice nor the allowance to do that.

Sertoli cell population size is set during puberty and, importantly, the number of germ cells and Leydig cells that can be maintained in the adult testis depends on the size of the adult Sertoli cell population (PMID: 3125042, 28911170). The author suggested that in the adult mutant testis, the reduction in Sertoli cell number is caused by their degeneration and shedding in the lumen of seminiferous tubules. However, given the defects found in mutant mice, the reduction in Sertoli cell is an important finding and should be further investigate (see below in comments to figure 5).

Reduced Sertoli cell size is an interesting finding, although it is not the reason for infertility in mice lacking importin α7. As it has not been the main finding of the study, we did not study it in detail, however, we extended our analysis by investigating Sertoli cell numbers in young (prepubertal) mice (Fig. 5A). Our new analysis show, that there is no difference in Sertoli cell numbers in 4 weeks old mice of all three genotypes, excluding a disturbed proliferation phase of Sertoli cells (that takes place before).

Figure 1: The novel finding of the germ cell-specific alternative intronic promoter upstream of E1A should be better illustrate. Are the alternative 5'-end been sequenced and compared to BY353738.1 sequence? Data in C suggest that both Kpna6 promoters are active in WT testis (amplification of both exon1/exon6 and exon1A/exon6 amplicons). Is the promoter upstream of E1A driving importin a7 expression in germ cells also in WT testis? Are the two promoters (upstream of E1 and E1A) both active in WT germ cells? This could be easily assessed using total RNA of FACS-sorted germ cells from WT and importin a7-/- mice. In C exon1/exon6 amplicons from liver and testis show different electrophoretic mobility. Are they different mRNA splicing variants? Figure S1 In B, the amount of importin a7 full length in a7ΔIBB/- testis is very similar to a7 -/- testis. This is surprisingly considering that the levels of importin a7 expression in a7-/- testis should be higher (if not double) respect to a7ΔIBB/- testis.

© 2021. Published by The Company of Biologists under the terms of the Creative Commons Attribution License (https://creativecommons.org/licenses/by/4.0/).

The testis-specific transcript has been sequenced after PCR analysis using a primer at the beginning of exon 1A. The sequence was identical to the database entries BY353738.1 and XM_006502814.4. This will be mentioned in the manuscript. We performed a Sashmi plot of the RNAseq data and confirmed that both promoters are active in the testis of wild-type animals (Fig. S2A). A newly performed RT-PCR of round spermatids showed, that in WT germ cells, both promoters are active, while in round spermatids of importin α7$^{-/-}$ mice, only the intronic promoter is active. Germ cells of importin α7$^{dIBB/dIBB}$ mice show only the amplification from exon1, as the sequence of exon1 has been replaced by the Neo^{R} cassette. The amplicons from liver and testis have been sequenced and are not different.
Fig. S1C is misleading. The GAPDH in α7$^{dIBB/-}$ shows much stronger bands than in α7$^{-/-}$. Moreover, the unspecific band above the specific importin α7 band is also stronger in α7$^{dIBB/-}$ than in α7$^{-/-}$. We performed quantification for the importin α7 signal of multiple Western Blots, which revealed that the amount of the full length importin α7 in α7$^{dIBB/-}$ is about 40% of the signal found in α7$^{-/-}$ (Fig. S1C).

Figure 2: C and D: is not clear why the diameter of the seminiferous tubules and epididymal sperm count are expressed as relative values to WT. The actual numerical values will be more informative for the readers.

Changes have been made in text and figures.

Figure 5: A: how is Sertoli cell population size in mutant vs WT during early testis postnatal development? Is the Sertoli proliferation phase in immature testis affected by importin α7 loss? C: AR is also expressed in peritubular cells. Is the nuclear AR expression also lost in peritubular cells of mutant mice?

A: We compared the number of Sertoli cells in 4 weeks old animals and detected no differences in numbers which argues against a disturbed proliferation phase (Fig. 5).
C: AR expression in peritubular cells is still present in all three analyzed groups and we could not detect clear differences in nuclear presence of AR in Leydig cells and peritubular myoid cells in all three groups. We attempted to repeat immunohistochemistry, but the antibodies that had been used for this study are not available anymore and despite testing several more antibodies we were not able to get a good and specific labelling of AR.

Figure 6: The misalignment of sperm head a7ΔIBB/ΔIBB and a7-/- mice suggests a defect in the ectoplasmic specialization. How is the distribution of F-actin at stage VIII in the three mice lines?

We were not able to get a clear F-actin staining by labelling with Phalloidin. The reason could be that F-actin ultrastructure has not been well preserved in the testis sections we used. Two different fixatives were tested (4% PFA and Buoin's solution). Finally, we labelled F-actin in testis cryosections. Here, the staining worked well, but it is not so easy to discriminate between the tubular stages. In WT and α7$^{-/-}$ testes, we could clearly identify stage VIII tubuli by arrangement of sperms in a line at the tubular lumen. However, in α7$^{dIBB/dIBB}$ this never happens as the tubular structure is disorganized and sperm maturation is retarded. In an attempt to compare the tubuli, we analysed the tubular regions where sperm chromatin is maximal compacted.
Although the typical F-actin ring that can be found in WT and α7$^{-/-}$ (arrows) does not appear in α7$^{dIBB/dIBB}$ (probably due to the fact that there are less sperms and that they are not properly arranged), the higher magnification pictures show F-actin signals in close proximity to sperms (arrowheads), leading to the conclusion, that F-actin staining in ectoplasmic specializations is not disturbed *per se*.

© 2021. Published by The Company of Biologists under the terms of the Creative Commons Attribution License (https://creativecommons.org/licenses/by/4.0/).

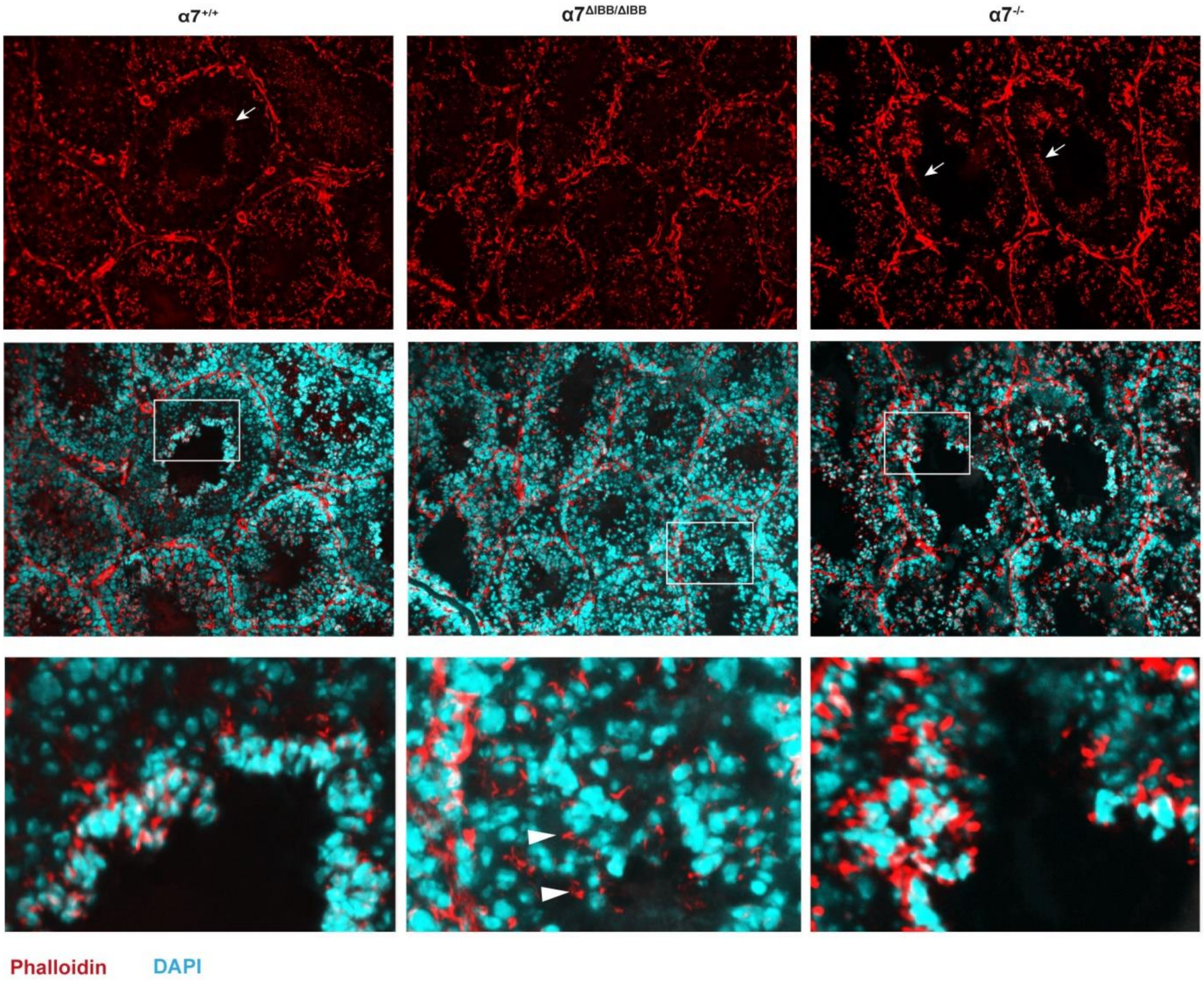

Figure 7: There is no clear indication about germ cells were recognized and counted. As for preleptotene spermatocytes, is the histogram showing the counts for BrdU-labelled S-phase preleptotene? What about BrdU-negative preleptotene? Are the pachytene spermatocytes recognized and counted based solely on γH2AX staining? At page 8, what is "the stage-specific appearance of γH2AX-positive chromatin"? are the authors referring to the γH2AX-staining pattern in the different germ cell types in a given stage? or are they indirectly referring to the absence of γH2AX-positive apoptotic cells? In mutant mice, is the reduction in leptotene/zygotene spermatocytes due to increased apoptosis?

The histogram shows the counts for BrdU-labelled S-phase preleptotene spermatocytes. Only stage VII and VIII tubuli were used for calculation. For counting of leptotene/zygotene spermatocytes their intense expression of γH2AX was used as marker. Pachytene spermatocytes were counted based on their nuclear shape and chromatin (DAPI) staining and their position in the tubuli. The "stage-specific appearance of γH2AX-positive chromatin" refers to the observation, that γH2AX is present in leptotene/zygotene spermatocytes. In addition, localization of γH2AX in sex bodies of pachytene spermatocytes as well in double strand breaks in elongating spermatids could be found in all three groups. Changes in the text have been made to make this clearer. We found increased levels of apoptosis in importin $α7^{dIBB/dIBB}$ by TUNEL staining (Fig. 7A). Interestingly, importin $α7^{-/-}$ testis did not display elevated apoptosis levels. Many of the TUNEL-positive cells localized to the areas of the tubules where pachytene spermatocytes are localized. Therefore we conclude, that reduction in pachytene spermatocytes is accompanied by increased apoptosis.

Figure 8: In the mouse testis γH2AX staining decorates all spermatogonial type not only B spermatogonia (Blanco- Rodriguez, 2009).

Changes in text have been made.

© 2021. Published by The Company of Biologists under the terms of the Creative Commons Attribution License (https://creativecommons.org/licenses/by/4.0/).

Figure 9 and related supplementary tables The transcriptome-wide analysis revealed significant downregulation of Kpna6 expression in a7ΔIBB/ΔIBB compared WT mice, as expected. However, the observation hat in these experiments Kpna6 show no significant differential expression between WT and a7 -/- is puzzling considering the data in Figure 1B, C showing a clear downregulation of Kpn6a at both mRNA and protein level.

Fig. 1B refers to the protein levels in whole testis. Despite the lower expression of GAPDH in importin α7$^{-/-}$ testis in the left panel, the reduction in importin α7 protein levels with both antibodies is very obvious. Quantification of Western Blots revealed a reduction to 57% of WT levels (with C-term. and N-term. antibodies).
Fig. 1C is not quantitative and no normalization to housekeeping genes was made. Comparing mRNA and protein data is challenging, as the importin α7$^{-/-}$ mouse contains a genetrap insertion. Some of the counted RNAs could originate from 5'-*Kpna6* fused with gene-trap. To address this issue further we analyzed the mRNA sequences found in the transcriptome-wide analysis. Here, the Sashmi Plot showed no differential expression of transcription starting with exon1A in importin α7$^{-/-}$ testis compared with WT (769 vs. 606, Fig. S2A). So, we conclude that despite comparable expression of the transcripts, the protein expression of importin α7 is reduced in importin α7$^{-/-}$ testes.

Figure 10 The micrographs do not clearly show the findings described in the text. How the abnormal nuclear condensation was assessed? Is it referred to chromatin condensation, nuclear reshaping or both? I suggest to show less cells along with a clear annotation of the different spermatid steps being compared between different mice lines for each stage of the cycle.

In Fig. 10, we show few cells with annotation of the tubular stage (grouped on the base of DAPI and peanut agglutinin (Acrosome-) staining). However, as the nature of the phenotype in importin α7$^{dIBB/dIBB}$ comes with a delay in spermatid maturation a certain disorder of cells makes the correct classification of the spermatid stages very difficult. We find it absolutely necessary, to show round spermatids together with elongating sperms, because at that stage the development (based on acrosomal shape) is still quite normal. To make the abnormalities more clear, we now labelled the sperm stages and round spermatid steps in the figure. As the reader can see, beginning from stage I tubules, the sperms in the importin α7$^{dIBB/dIBB}$ group are not labelled anymore, because at this time, the delayed maturation becomes very obvious. The abnormalities found in sperm nuclei refer to the nuclear shape.

Figure 11: The data presented are from a7ΔIBB/ΔIBB and WT mice lines. Why the a7 -/- mice line is not included in the analysis? How is the level of Tpn1 and Tpn2 and protamines in round spermatids isolated from a7 -/- testis? is their expression levels restored by importin a7 full-length? B: it is not clear what the histogram stands for. Does it show the results from western blot quantification? why the western blot is not shown? how the levels of chromatin-bound Tnp2 were normalized?

We performed a FACS sorting of testis cells of importin α7$^{-/-}$ mice and complete the quantitative PCRs. As expected, we did not see differences in the expression of protamines and transition nuclear proteins in this group (Fig. S5) compared to WT. The histogram in B stands for the Western blot quantification and we added the original western blot picture to the figure. The levels of chromatin-bound Tnp2 were normalized to total protein levels.

Figure 12: Brwd1 staining pattern in WT testis is not convincing. Published data suggest that Brdwd1 should be detected in spermatocyte and round spermatids (PMID: 25547156). Moreover, I assume, Brdw1 should have a nuclear localization. In the micrographs, it's no clear if Brwd1 is expressed in spermatocytes and round spermatids and whether the dotted Brwd1 immunoreactivity is localized in the nuclei. What about Brdw1 expression in a7 -/- mice?

We tested two different antibodies for Brwd1 labelling. One of them did not work in immunofluorescence, the other one showed repeatedly a very specific pattern in WT and importin importin α7$^{-/-}$ testis sections with no differences between these two groups. However, this staining pattern was completely missing in α7$^{dIBB/dIBB}$. The referred publication (PMID: 25547156) showed expression of Brwd1 in transfected cells in the nucleus which is an artificial expression compared to

© 2021. Published by The Company of Biologists under the terms of the Creative Commons Attribution License (https://creativecommons.org/licenses/by/4.0/).

tissues. Moreover, here Brwd1 was exclusively bound to chromatin. Concerning native expression of the protein, there don't exist many data. The human protein atlas shows expression in round spermatids and earlier spermatocytes, however, the localization is not pronounced and depending of the antibody used, different staining patterns (nuclear, cytoplasmic, both, membranous) can be found. (https://www.proteinatlas.org/ENSG00000185658-BRWD1/tissue/testis#img) So, there is no conclusion from literature about the correct *in vivo* localization of Brwd1. A third Brwd1 antibody has meanwhile been tried out. Unfortunately, it did not work in histology. All available antibodies are generated against the human Brwd1 protein (or peptides) and most of them are only recommended for the use in Western Blot. Western blot with testis extracts showed, that Brwd1 expression is not altered in importin $\alpha7^{dIBB/dIBB}$ mice compared to the two other groups. This analysis was added to the manuscript (Fig. 12C). We conclude that the staining pattern seen in both paraffin-and cryosectioned testis represents rather a differential localization than a difference in nabsolute protein level. The dotted staining pattern (occuring predominantly in stage IV-VI tubules) could represent an accumulation of Brwd1 before it is degraded or shedded within residual bodies.

Second decision letter

MS ID#: DEVELOP/2020/198374

MS TITLE: Importin α7 deficiency causes infertility in male mice by disrupting spermatogenesis

AUTHORS: Na Liu, Fatimunnisa Qadri, Hauke Busch, Stefanie Huegel, Gabin Sihn, Ilya Chuykin, Enno Hartmann, Michael Bader, and Franziska Rother

I have now received all the referees' reports on the above manuscript. The referees' comments are appended below, or you can access them online: please go to BenchPress and click on the 'Manuscripts with Decisions' queue in the Author Area.

As you will see, the referees recognise that the manuscript has been improved and that most of the concerns raised in the previous review were addressed. However, some significant criticisms remained and the referees were not convinced that the revised manuscript warrants publication at this juncture. While normally only one round of major revision of the manuscript is allowed, we are always keen to offer the opportunity for authors to defend and improve the manuscript. If you are able to address the critiques to your best ability and revise the manuscript along the lines suggested, I will consider receiving another revised version of the manuscript. Your revised paper will be re-reviewed by one or more of the original referees, and acceptance of your manuscript will depend on your addressing satisfactorily the reviewers' concerns. Please note that this will be the last round of revision.

We are aware that you may be experiencing disruption to the normal running of your lab that make experimental revisions challenging. If it would be helpful, we encourage you to contact us to discuss your revision in greater detail. Please send us a point-by-point response indicating where you are able to address concerns raised (either experimentally or by changes to the text) and where you will not be able to do so within the normal timeframe of a revision. We will then provide further guidance. Please also note that we are happy to extend revision timeframes as necessary.

Please attend to all of the reviewers' comments and ensure that you clearly highlight all changes made in the revised manuscript. Please avoid using 'Tracked changes' in Word files as these are lost in PDF conversion. I should be grateful if you would also provide a point-by-point response detailing how you have dealt with the points raised by the reviewers in the 'Response to Reviewers' box. If you do not agree with any of their criticisms or suggestions please explain clearly why this is so.

Reviewer 1

Advance summary and potential significance to field

© 2021. Published by The Company of Biologists under the terms of the Creative Commons Attribution License (https://creativecommons.org/licenses/by/4.0/).

I understand that the authors made many efforts to clarify the possible molecular mechanism of how importin a7 contributes to spermatogenesis. Unfortunately, there is no clear answer. Nevertheless, the mouse models are well characterized and the analyses are conducted well, but authors should make a more reasonable description. I still had difficulty reading this manuscript because of some flaws and discrepancy in the interpretation of data presented. I recommend authors to reduce description for negative data. Although those are also important information, it is better to describe them more concisely. Because readers expect a clear answer but it is often betrayed. Please try to use the space to describe more meaningful description and discussion.

Comments for the author

Major comments
1. Up to 4 weeks, there is no reduction of Sertoli number (Fig. 5A), however there are some common defects in germ cell differentiation (Fig. 8B, 8C). I guess that this defect in differentiation observed in both α7ΔIBB/ΔIBB and α7-/- could be due to defects in Sertoli cells. However, the authors seem not to think so.
I could not understand how the authors interpret the cause of the loss of pachytene spermatocytes. Because meiotic germ cells do not express importin a7 yet, suggesting that this defect is caused by defective Sertoli function. Using antibodies, the authors clearly showed that KPNA6 is expressed in Sertoli cells and germ cells after meiosis. However, they refer to previous mRNA data and try to explain the early defect could be due to the function of germ cells. I could not understand this scenario. If the authors want to discuss and interpret phenotype, they should consider protein expression patterns but not mRNA.

2. Fig. 5C. Please show which area is enlarged. According to these pictures, AR expression itself may be reduced. Is there any clear picture showing the defect is nuclear localization? If the authors really want to compare gene expression change of AR and AR-related or Sertoli specific transcripts, they should isolate Sertoli cells to compare transcriptome or conduct in situ hybridization. Analysis using the whole testis is meaningless. To ask for a BTB function may be reasonable, but this section could be shortened by reducing non-informative analyses and descriptions.

3. They found that Tnp2 mRNA and protein expressions are markedly reduced in α7ΔIBB/ΔIBB mice. However, the immunostaining data indicate that the expression is observed in α7ΔIBB/ΔIBB testes and the expression was prolonged. I could not understand how the authors solve this discrepancy.

4. Regarding RFX2 As I mentioned before and reviewer #2 pointed out, transcriptome analyses using whole testis is difficult to compare each genotype since cell population is clearly changed. However, they used the same amount of RNA as the material. Due to the response to reviewer #2, the authors did not make compensations for the difference in cellularity. However, they now isolated round spermatids and compared gene expression, but I could not see the result of Rfx2. In addition they now showed the expression pattern and the localization of RFX2 were not different between three genotypes. I am wondering whether this description for RFX2 is required or not.

5. BRWD1 The authors finally found a clear expression difference. However, the authors ended by showing western blot using whole testis extracts, which is meaningless.
There is no meaningful discussion regarding the loss of BRWD1. Since they have the ability to FACS sort of round spermatid, they could have used the population for quantification although I do not think that it is necessary.

Minor

1. Abstract:
"Furthermore, our data demonstrate devastating defects in spermiogenesis that are accompanied by disturbed histone-protamine-exchange, differential localization of the transcriptional regulator Brwd1 and altered expression of Rfx2 target genes, resulting in incomplete sperm maturation and massive loss of sperms."

The causal relationship is not clearly shown.

© 2021. Published by The Company of Biologists under the terms of the Creative Commons Attribution License (https://creativecommons.org/licenses/by/4.0/).

2. I asked to describe the age of mice used for each experiment in figure (better) or figure legends. The information is still missing for some data.
Which age is for adult mice. Is there any testis size change between young and old mice. Is there any progressive change in testis size or morphology?

Reviewer 2

Advance summary and potential significance to field

This remains as in previous review:
This manuscript presents comprehensive and deep phenotyping of testis structure and function in mice lacking importin a7, a protein correctly referred to as KPNA6 by both MGI and HUGO (the human gene is KPNA6, italicized, and the mouse gene is Kpna6, italicized). Overall, the authors show that lack of KPNA6 in testes leads to multiple spermatogenesis defects and abnormalities of the Sertoli cells; excellent figures support these conclusions. Nonetheless, impact is limited because primary and downstream effects cannot be resolved.

Comments for the author

The manuscript has been moderately and somewhat haphazardly revised in response to the three previous critiques. The Discussion has been minimally shortened, but not restructured to be more direct. Many minor issues raised by the reviewers have been addressed; however, the major issues of confusing models and phenotypes and lack of mechanistic insights have not been resolved. Thus, as the authors state in the last sentence of the Discussion, much work remains to be done to resolve developmental pathways.

Reviewer 3

Advance summary and potential significance to field

Importin α family of proteins are involved in the nucleocytoplasmic transport, an essential process in cell physiology. Several importin α are expressed in mammalian testis and differentially expressed as germ cells progress through mitotic, meiotic and haploid maturation. However, little is known about their function. In their manuscript Liu and collaborators analyzed the testicular phenotype of importin α7 knockout taking advantage of two different animal models, previously generated in their laboratory. In α7ΔIBB/ΔIBB mouse line the genomic modification of the Kpna6 gene induces the production of a truncated protein while in the α7−/− knockout mouse line, importin α7 is completely deleted in all tissues.
The homozygous females of both mice lines are infertile and this prompt the authors to further scrutinize the reproductive phenotype in males. In this manuscript the authors reported that male α7−/− male were found to be fertile while α7ΔIBB/ΔIBB were sterile. Interestingly, in α7−/− male even though the expression of the full-length importin α7 was abrogated in all tissues, in the testis it was partially restored due to an intronic alternative promoter driving the expression of the full-length importin α7 in germ cells. The authors showed that importin α7 protein is expressed in the nuclei of round spermatids, elongating spermatids and Sertoli cells.
Importantly, importin α7 deficiency results in several defects during spermiogenesis including incomplete sperm maturation, germ cell loss and oligozoospermia in both mutant mouse lines. The authors main claim is that importin α7 is essential for Sertoli cell function and regulation of spermatogenesis. This is the first manuscript that reveal an essential function for an importin α member in mammalian spermatogenesis, underscoring the importance of the nucleocytoplasmic transport during germ cell maturation.

Comments for the author

Liu and colleagues have responded carefully ad accurately to all my suggestions for the improvement of their manuscript. Additional experiments were performed and importantly, the authors provided new data in revised manuscript that contributes to clarify the observed phenotypes.

© 2021. Published by The Company of Biologists under the terms of the Creative Commons Attribution License (https://creativecommons.org/licenses/by/4.0/).

Second revision

Author response to reviewers' comments

Dear Dr. Tam,

thank you for considering our manuscript for a second revision thereby giving us the chance to further improve the manuscript. We have carefully worked on the text and added new information. In addition to answering the specific questions of reviewer #1 and #2, we have addressed the general suggestions of both reviewers and restructured major parts of the discussion and shortened the manuscript.

Reviewer 1 Advance Summary and Potential Significance to Field:
I understand that the authors made many efforts to clarify the possible molecular mechanism of how importin α7 contributes to spermatogenesis. Unfortunately, there is no clear answer. Nevertheless, the mouse models are well characterized and the analyses are conducted well, but authors should make a more reasonable description. I still had difficulty reading this manuscript because of some flaws and discrepancy in the interpretation of data presented. I recommend authors to reduce description for negative data. Although those are also important information, it is better to describe them more concisely. Because readers expect a clear answer but it is often betrayed. Please try to use the space to describe more meaningful description and discussion.

We worked carefully on the text and described the data more concisely putting more emphasis on positive and less emphasis on negative data. The discussion was extensively rearranged and shortened. But we decided against leaving out negative data as we believe that those address relevant mechanisms which could have been involved in the observed phenotype.

Comments for the Author:
1. Up to 4 weeks, there is no reduction of Sertoli number (Fig. 5A), however, there are some common defects in germ cell differentiation (Fig. 8B, 8C). I guess that this defect in differentiation observed in both α7ΔIBB/ΔIBB and α7-/- could be due to defects in Sertoli cells. However, the authors seem not to think so. I could not understand how the authors interpret the cause of the loss of pachytene spermatocytes. Because meiotic germ cells do not express importin α7 yet, suggesting that this defect is caused by defective Sertoli function. Using antibodies, the authors clearly showed that KPNA6 is expressed in Sertoli cells and germ cells after meiosis. However, they refer to previous mRNA data and try to explain the early defect could be due to the function of germ cells. I could not understand this scenario. If the authors want to discuss and interpret phenotype, they should consider protein expression patterns but not mRNA.

The defects in germ cell differentiation observed in both mouse lines during the first round of spermatogenesis can be attributed to a dysfunction of Sertoli cells that lack importin α7 in both lines. The manuscript text (results and discussion) was changed to state this more clearly. In contrast, only $α7^{ΔIBB/ΔIBB}$, but not $α7^{-/-}$ mice exhibit a loss of pachytene spermatocytes. Although we were not able to detect a clear importin α7 protein staining in pachytene spermatocytes, previous studies have shown that mRNA expression starts at the level of pachytene spermatocytes, which led us to the conclusion that low levels of protein might be already expressed at this time point and could be crucial for the fate of these cells.

2. Fig. 5C. Please show which area is enlarged. According to these pictures, AR expression itself may be reduced. Is there any clear picture showing the defect is nuclear localization? If the authors really want to compare gene expression change of AR and AR-related or Sertoli specific transcripts, they should isolate Sertoli cells to compare transcriptome or conduct in situ hybridization. Analysis using the whole testis is meaningless. To ask for a BTB function

© 2021. Published by The Company of Biologists under the terms of the Creative Commons Attribution License (https://creativecommons.org/licenses/by/4.0/).

may be reasonable, but this section could be shortened by reducing non-informative analyses and descriptions.

Fig. 5C was improved. Our experiments and Fig. 5C show clearly that the steady-state nuclear localization of AR is disturbed in Sertoli cells of α7$^{ΔIBB/ΔIBB}$ and α7$^{-/-}$ mice. Using immunohistological staining of tissue sections, it is impossible to investigate the dynamics of nuclear translocation. To answer this question, in vitro experiments are required (e.g. nuclear import assays). We did not find gross accumulation of AR in Sertoli cell cytoplasm. There could be different reasons for this: 1) the cytoplasm of Sertoli cells is very large spanning from the basement membrane to the lumen of seminiferous tubuli which could lead to a dilution effect; 2) AR might be degraded, if it does not enter the nucleus.
We agree that the analysis of gene expression related to AR or Sertoli cells would be interesting to conduct on isolated Sertoli cells. However, in the case of importin α7$^{ΔIBB/ΔIBB}$ and α7$^{-/-}$ mice, the Sertoli cell related phenotype is minor compared to the important alterations seen when importin α7 is missing in germ cells, that is why we concentrated on the more prominent and obvious phenotype. Also the paragraph describing BTB function was further shortened and rearranged, putting more emphasis on positive data.

3. They found that Tnp2 mRNA and protein expressions are markedly reduced in a7ΔIBB/ΔIBB mice. However, the immunostaining data indicate that the expression is observed in a7ΔIBB/ΔIBB testes and the expression was prolonged. I could not understand how the authors solve this discrepancy.

Our data show a reduction of Tnp2 mRNA in isolated round spermatids of importin α7$^{ΔIBB/ΔIBB}$ mice. Furthermore, protein expression of Tnp2 was reduced in whole testis of α7$^{ΔIBB/ΔIBB}$ mice. The prolonged expression of Tnp2 protein observed in later stages does not contradict its reduced expression. As the former is a static observation (amount of mRNA or protein calculated to amount of cells) and the latter is a dynamic observation (at which stage Tnp2 protein is expressed), we do not see a discrepancy.
To make this more clear, we include a picture of Tnp2 staining of the testis at low magnification. The picture shows, that in importin α7$^{ΔIBB/ΔIBB}$ testis the signal for Tnp2 is weaker compared to the other two lines. However, while wildtype and importin α7$^{-/-}$ testes display tubuli that are either positive (stage IX-III) or negative (stage IV-VIII, arrows), importin α7$^{ΔIBB/ΔIBB}$ testes show Tnp2 signals in tubuli of all stages.

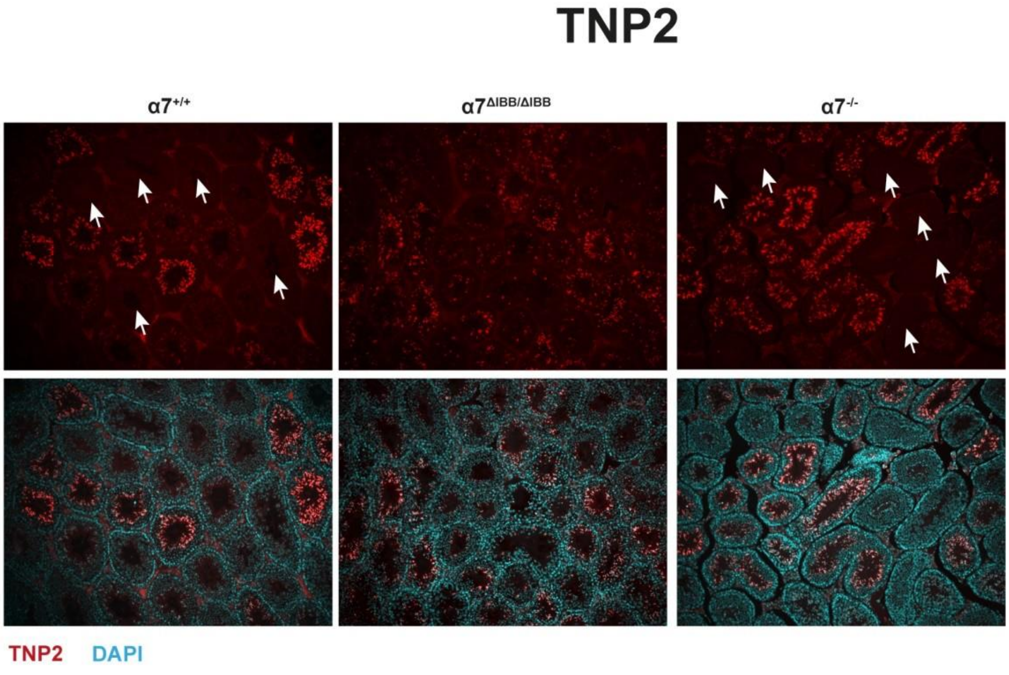

© 2021. Published by The Company of Biologists under the terms of the Creative Commons Attribution License (https://creativecommons.org/licenses/by/4.0/).

4. Regarding RFX2: As I mentioned before and reviewer #2 pointed out, transcriptome analyses using whole testis is difficult to compare each genotype since cell population is clearly changed. However, they used the same amount of RNA as the material. Due to the response to reviewer #2, the authors did not make compensations for the difference in cellularity. However, they now isolated round spermatids and compared gene expression, but I could not see the result of Rfx2. In addition, they now showed the expression pattern and the localization of RFX2 were not different between three genotypes. I am wondering whether this description for RFX2 is required or not.

The RNAseq data showed a disturbed RFX2-related pathway but we could not find a disturbed RFX2 expression or nuclear localization. That is why we moved these data to the supplement and discuss possible ways by which RFX2 could anyhow be involved in the phenotype observed. We suggest, that a cofactor or interacting partner of RFX2 that is needed for correct transcription, might be dysregulated.

5. BRWD1: The authors finally found a clear expression difference. However, the authors ended by showing western blot using whole testis extracts, which is meaningless. There is no meaningful discussion regarding the loss of BRWD1. Since they have the ability to FACS sort of round spermatid, they could have used the population for quantification although I do not think that it is necessary.

We rearranged the data, taking out Fig. 12B and C (immunohistochemistry of BRWD1 on cryosections and Western Blot) and moving them to Supplemental data (Fig. S7A and B). The discussion on the loss of BRWD1 was extended.

Minor

1. Abstract: "Furthermore, our data demonstrate devastating defects in spermiogenesis that are accompanied by disturbed histone-protamine-exchange, differential localization of the transcriptional regulator Brwd1 and altered expression of Rfx2 target genes, resulting in incomplete sperm maturation and massive loss of sperms." The causal relationship is not clearly shown.

The text was changed.

2. I asked to describe the age of mice used for each experiment in figure (better) or figure legends. The information is still missing for some data. Which age is for adult mice. Is there any testis size change between young and old mice. Is there any progressive change in testis size or morphology?

Every figure legend now describes the age of mice we used. We included a graph for progressive change of testis size and weight of importin $\alpha7^{-/-}$ compared to wildtype mice in Figure S1.

Reviewer 2 Advance Summary and Potential Significance to Field:

This remains as in previous review: This manuscript presents comprehensive and deep phenotyping of testis structure and function in mice lacking importin α7, a protein correctly referred to as KPNA6 by both MGI and HUGO (the human gene is KPNA6, italicized, and the mouse gene is Kpna6, italicized). Overall, the authors show that lack of KPNA6 in testes leads to multiple spermatogenesis defects and abnormalities of the Sertoli cells; excellent figures support these conclusions. Nonetheless, impact is limited because primary and downstream effects cannot be resolved.

Reviewer 2 Comments for the Author: The manuscript has been moderately and somewhat haphazardly revised in response to the three previous critiques. The Discussion has been minimally shortened, but not restructured to be more direct. Many minor issues raised by the reviewers have been addressed; however, the major issues of confusing models and phenotypes and lack of mechanistic insights have not been resolved. Thus, as the authors state in the last sentence of the Discussion, much work remains to be done to resolve developmental pathways.

© 2021. Published by The Company of Biologists under the terms of the Creative Commons Attribution License (https://creativecommons.org/licenses/by/4.0/).

We worked again carefully on the text and described the data more concisely putting more emphasis on positive and less emphasis on negative data. The discussion has been extensively restructured and shortened. To avoid the confusion on the two animal models, we now state more clearly their basic characteristics, one lacking importin α7 in all cells and one having it only expressed in germ cells, repeating the information in the revised Fig. 1A.

Reviewer 3 Advance Summary and Potential Significance to Field:
Importin a family of proteins are involved in the nucleocytoplasmic transport, an essential process in cell physiology. Several importin a are expressed in mammalian testis and differentially expressed as germ cells progress through mitotic, meiotic and haploid maturation. However, little is known about their function. In their manuscript Liu and collaborators analyzed the testicular phenotype of importin a7 knockout taking advantage of two different animal models, previously generated in their laboratory. In a7ΔIBB/ΔIBB mouse line the genomic modification of the Kpna6 gene induces the production of a truncated protein while in the a7−/− knockout mouse line, importin a7 is completely deleted in all tissues. The homozygous females of both mice lines are infertile and this prompt the authors to further scrutinize the reproductive phenotype in males. In this manuscript the authors reported that male a7−/− male were found to be fertile while a7ΔIBB/ΔIBB were sterile. Interestingly, in a7−/− male even though the expression of the full-length importin a7 was abrogated in all tissues, in the testis it was partially restored due to an intronic alternative promoter driving the expression of the full-length importin a7 in germ cells. The authors showed that importin a7 protein is expressed in the nuclei of round spermatids, elongating spermatids and Sertoli cells. Importantly, importin a7 deficiency results in several defects during spermiogenesis including incomplete sperm maturation, germ cell loss and oligozoospermia in both mutant mouse lines. The authors main claim is that importin a7 is essential for Sertoli cell function and regulation of spermatogenesis. This is the first manuscript that reveal an essential function for an importin a member in mammalian spermatogenesis, underscoring the importance of the nucleocytoplasmic transport during germ cell maturation.
Reviewer 3 Comments for the Author: Liu and colleagues have responded carefully ad accurately to all my suggestions for the improvement of their manuscript. Additional experiments were performed and importantly, the authors provided new data in revised manuscript that contributes to clarify the observed phenotypes.

Thank you for this encouraging comment.

Third decision letter

MS ID#: DEVELOP/2020/198374

MS TITLE: Importin α7 deficiency causes infertility in male mice by disrupting spermatogenesis

AUTHORS: Na Liu, Fatimunnisa Qadri, Hauke Busch, Stefanie Huegel, Gabin Sihn, Ilya Chuykin, Enno Hartmann, Michael Bader, and Franziska Rother
ARTICLE TYPE: Research Article

I am satisfied with your response to review and the revision of the manuscript. This manuscript has been accepted for publication in Development, pending our standard ethics checks.

© 2021. Published by The Company of Biologists under the terms of the Creative Commons Attribution License (https://creativecommons.org/licenses/by/4.0/).
